# Chasing Self‐Assembly of Thioether‐Substituted Flavylium Salts in Solution and Bulk State

**DOI:** 10.1002/cphc.202200154

**Published:** 2022-05-17

**Authors:** Julius A. Knöller, Robert Forschner, Wolfgang Frey, Johannes Lang, Angelika Baro, Anna Zens, Yann Molard, Frank Giesselmann, Birgit Claasen, Sabine Laschat

**Affiliations:** ^1^ Institut für Organische Chemie Universität Stuttgart Germany; ^2^ University of Rennes CNRS, ISCR, UMR 6226, ScanMAT – UMS 2001 Rennes France; ^3^ Institut für Physikalische Chemie Universität Stuttgart Germany

**Keywords:** DOSY, flavylium salts, ionic liquid crystals, liquid crystals, NMR methods

## Abstract

Two series of flavylium triflates carrying alkoxy side chains in the A‐ring (benzo unit of chromylium salt) and thioethers in the B ring (phenyl unit) (**O_n_**‐**Fla**‐**S_m_**) as well as thioethers at both A and B ring (**S_n_**‐**Fla**‐**S_m_**) were synthesized in order to understand the effect of thioether functionalization on their self‐assembly and electronic properties. Concentration‐dependent and diffusion ordered (DOSY) NMR experiments of **O_1_**‐**iV**‐**Fla**‐**S_3_** indicate the formation of columnar H‐aggregates in solution with antiparallel intracolumnar stacking of the AC unit (chromylium) of the flavylium triflate, in agreement with the solid state structure of **O_1_**‐**V**‐**Fla**‐**S_1_**. Thioether substitution on the B ring changes the linear optical properties in solution, whereas it has no effect on the A ring. According to differential scanning calorimetry, polarizing optical microscopy and X‐ray diffraction bulk self‐assembly of these ionic liquid crystals (ILCs) depends on the total number of side chains, yielding SmA and Lam_Col_ phases for ILCs with 2–3 chains and Col_ro_, Col_h_ phases for ILCs with 3–6 chains. Thus, we demonstrated that thioethers are a useful design tool for ILCs with tailored properties.

## Graphical abstract

**Sulfur for stability**! The more sulfur the better columnar phases are stabilized in flavylium ILCs. In solution thioethers particularly at the B ring exert an auxochromic effect on the absorption spectra and lead to antiparallel columnar stacking.

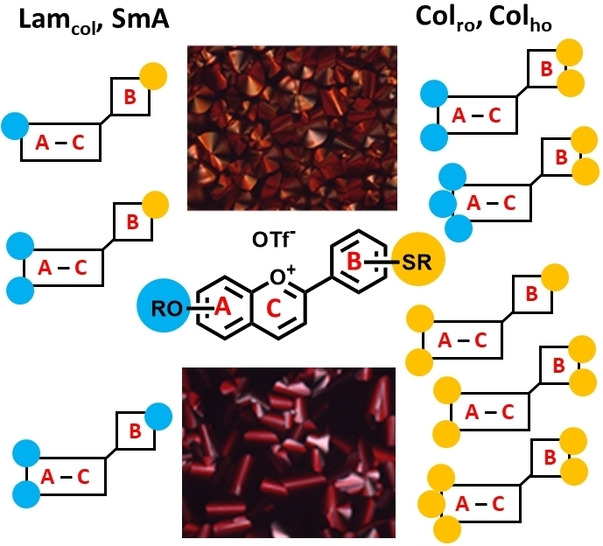

## Introduction

The combination of liquid crystalline materials with dye properties leads to highly attractive hybrid materials with unique self‐assembly behavior and physical properties, which are suitable for a broad variety of applications ranging from non‐linear optics,^[1]^ photoresponsive and photoalignable materials,^[2]^ dichroic, chromonic and laser dyes,^[3]^ to electrochromic materials^[4]^ and organic solar cells.^[5]^ For many of these applications, solution processing and proper alignment of the liquid crystalline dyes are critical issues. In most cases, long alkoxy side chains attached to the mesogenic core increase the solubility in organic solvents and promote mesophase formation through van der Waals interactions. Otherwise, there is increasing experimental evidence from comparative studies that the grafting of alkylsulfanyl (thioether) side chains to the aromatic dye cores instead of alkoxy chains can have manifold beneficial effects on the performance of these liquid crystalline materials. For example, it was reported that the replacement of alkoxy by alkylsulfanyl chains in non‐mesomorphic benzo[1,2‐*b*:4,5‐*b’*]bis[*b*]benzothiophenes led to increased melting points, antiperiplanar conformations and tighter packing in the solid state due to S^**…**^S interactions.^[6]^ For H‐bonded, mesomorphic benzoic acids phase transition temperatures of thioether‐substituted derivatives were lower than the alkoxy analogues due to S^**…**^S interactions favoring smectic clusters in the nematic phase.^[7]^ Rich polymorphism was observed for asymmetrically end‐capped oligothiophenes upon attachment of thioethers.^[8]^ Upon S/O replacement in the side chains of calamitic imidazolium ILCs, a pronounced decrease of the melting temperatures was detected, while clearing transitions remained constant or even increased, resulting in significantly broadened mesophase ranges.^[9]^ For methylimidazolium ILCs the emergence of mesomorphic properties via the sulfur motif was reported.^[10]^ Stabilization of mesophases was also found for columnar palladium complexes with dipicolinic acid‐derived pincer ligands^[11]^ and columnar crown ethers carrying thioethers instead of alkoxy side chains.^[12]^ Furthermore, the S/O replacement led to a higher degree of order in columnar mesophases of 6‐oxoverdazyl radicals.^[13]^ In calamitic tolane derivatives, the S/O replacement resulted in a strong increase of the birefringence.^[14]^

**Figure 1:**
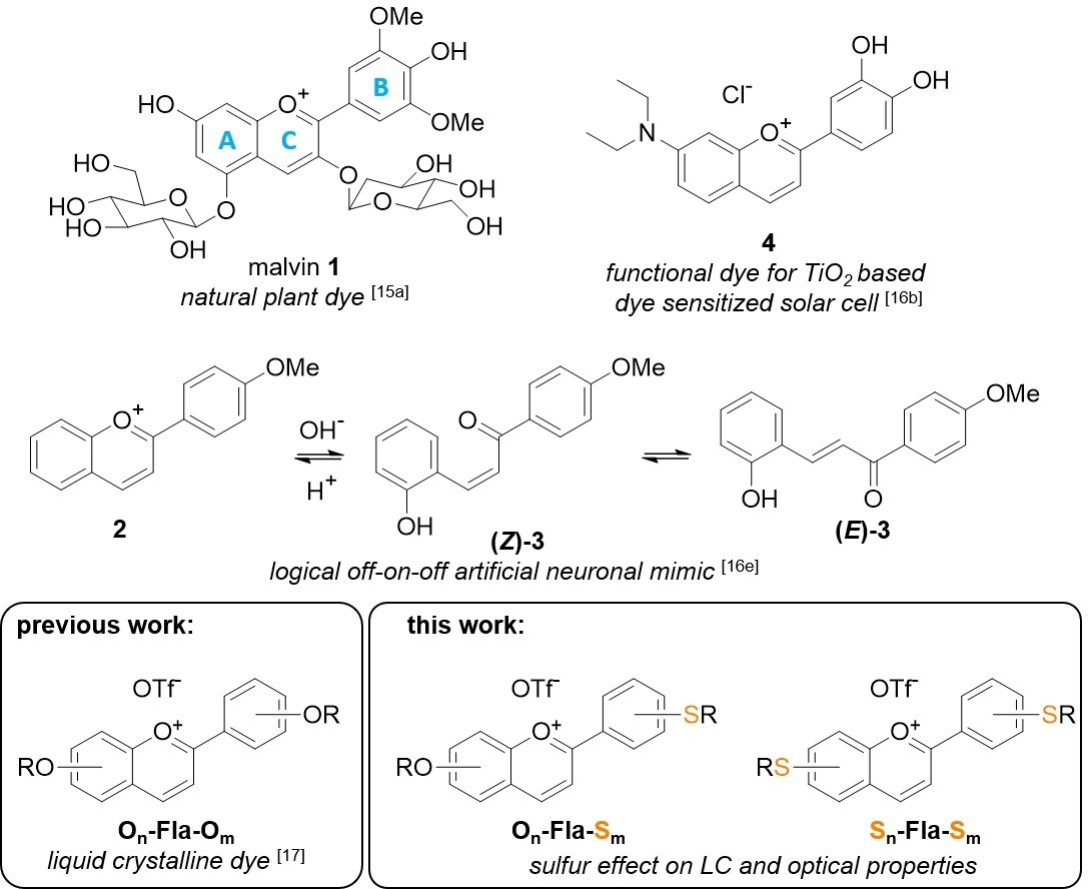
Flavylium salts found in nature (**1**),^[15a]^ as sensitizers for organic solar cells (**4**)^[16b]^ and as neuronal mimics (**2**,**3**)^[16e]^ as well as liquid crystalline flavylium salts **O_n_**‐**Fla**‐**O_m_** ^[17]^ and new sulfur analogues **O_n_**‐**Fla**‐**S_m_**, **S_n_**‐**Fla**‐**S_m_**_._

Regarding the chromophoric part in liquid crystalline dyes, flavylium salts are very attractive. Flavylium salts, i. e. benzo‐anellated 2‐phenyloxonium salts belong to a large class of plant dyes (Figure 1).^[15]^ Typical examples are malvin **1** and its aglycon malvidin.^[15a]^ The presence of hydroxy groups in natural flavylium salts enables pH‐dependent color shifts. The manifold substitution patterns found in nature lead to a large library of dyes^[15]^ with many applications.^[16]^ Besides their use as food colorants,^[16d]^ synthetic flavylium salts have also been employed in more technical applications, such as logical “off‐on‐off” artificial neuronal mimics **2**, **3** ^[16e]^ and photosensitizers **4** for Grätzel‐type solar cells.[^16a^, ^16b^, ^16c^, ^16f^]

We have recently developed flavylium‐based^[17]^ ionic liquid crystals^[18]^ (ILCs) **O_n_**‐**Fla**‐**O_m_** (R=H, alkyl), which combine the birefringence and self‐assembly of thermotropic neutral liquid crystals with the electrostatic interactions and tunability of polarity and solubility characteristic of ionic liquids as well as the absorption and emission characteristics of the flavylium chromophore (Figure 1). As our previous work emphasized the vital role of the substitution pattern on the mesomorphic properties of the flavylium ILCs, we were curious to highlight to what extent grafting of alkylsulfanyl side chains to the flavylium A and B rings (Figure 1) might affect the polymorphism, as well as the optical properties. Kaszynski^[19]^ and our group^[12]^ reported independently, that the attachment of thioethers to columnar mesogens increased the temperature range and enabled room temperature mesomorphism. These results motivated us to explore both thioether‐substituted flavylium salts **O_n_**‐**Fla**‐**S_m_** carrying thioethers only at the B‐ring and flavylium salts **S_n_**‐**Fla**‐**S_m_** carrying thioethers at both A and B ring. Solution NMR and X‐ray diffraction studies were performed to gain insight into their self‐assembly. As detailed below in the current manuscript, we report that the position of the thioether, i. e. A vs. B ring has a major impact on the self‐assembly in solution and bulk state.

## Results and Discussion

### Synthesis of Thioether‐Substituted Flavylium ILCs

**Scheme 1:**
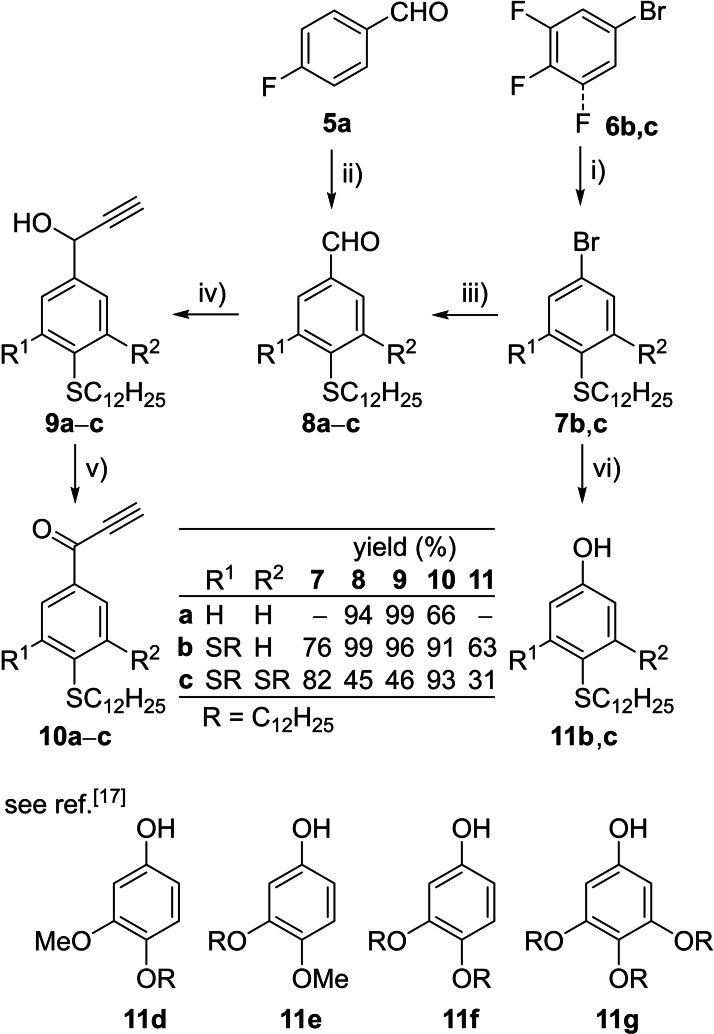
i) NaH, C_12_H_25_SH, DMSO, THF, 20 °C, 4 h, 60 °C, 6 h; ii) Na_2_CO_3_, C_12_H_25_SH, abs. DMSO, 160 °C, 1 d; iii) 1) *n*‐BuLi, abs. THF, −78 °C, 2 h, 2) DMF, 1 h; iv) HCCMgBr, abs. THF, rt, 4 h; v) IBX, EtOAc, 80 °C, 16 h; vi) H_2_O_2_, H_2_SO_4_, CHCl_3_, MeOH, 16 h; vii) Cu(acac)_2_ (10 mol%), BHMPO (10 mol%), LiOH•H_2_O, DMSO/H_2_O (4 : 1), 120 °C, 6 d; * The alkoxy derivatives **10 d**–**g** were prepared according to literature procedures.^[17]^

In order to access the two libraries of flavylium ILCs **O_n_**‐**Fla**‐**S_m_** carrying alkoxy chains on the A ring and alkylsulfanyl chains on the B ring and flavylium ILCs **S_n_**‐**Fla**‐**S_m_** carrying alkylsulfanyl side chains on both A and B ring, the previously described strategy by Chassaing was employed, where the flavylium core is generated by acid‐mediated cyclocondensation of phenols and propargylic ketones.[^17^, ^20^] As shown in Scheme 1, 4‐fluorobenzaldehyde **5 a** was treated with dodecanethiol in the presence of Na_2_CO_3_ adapting a procedure of Prasad^[21]^ yielding **8 a** in 94 %. The corresponding aldehydes **8 b**,**c** with two and three alkylsulfanyl chains were prepared according to Jankowiak^[19]^ from known bromides **7 b**,**c** ^[19]^ by formylation with *n*BuLi, DMF to give **8 b** in 76 % and **8 c** in 82 % respectively.

By following the protocol by Chassaing^[20]^, the aldehydes **8 a**–**c** were reacted with ethynyl magnesium bromide in THF to give the secondary propargylic alcohols **9 a**–**c**, which were submitted to IBX oxidation to the ketones **10 a**–**c** in 46–99 % (over 2 steps). In order to obtain the required phenols **11 b**,**c**, bis‐ and trisdodecyloxysulfanylphenylbromides **7 b**,**c** ^[19]^ were treated with LiOH in DMSO / H_2_O (4 : 1) in the presence of 10 mol% of Cu(acac)_2_ and 10 mol% of BHMPO using the method by Ma^[22]^ to yield **11 b**,**c** in 63 % and 31 % respectively.

**Scheme 2:**
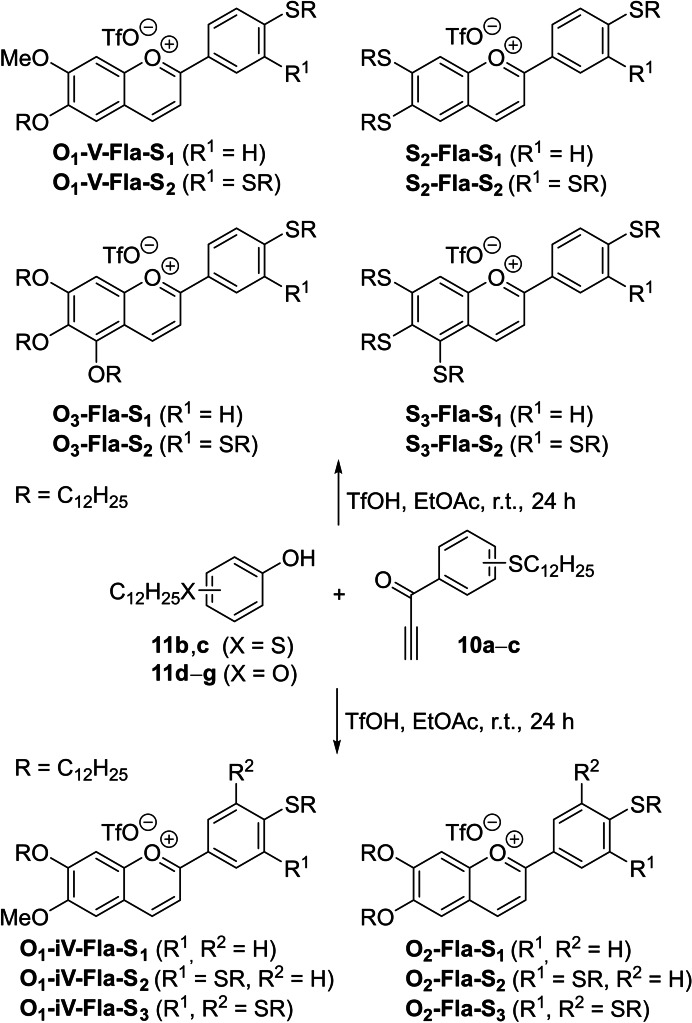
Synthesis of the flavylium salts **O_n_**‐**Fla**‐**S_m_** and **S_n_**‐**Fla**‐**S_m_** from the ketones **10 a**‐**c** and phenols **11 a**‐**g** as well as substrate scope.

With the desired phenols **11 b**–**g** including the known bis‐ or trisalkoxy‐substituted phenols **11 d**–**g** and the propargylic ketones **10 a**–**c** at hand, the flavylium salts **O_n_**‐**Fla**‐**S_m_**, **S_n_**‐**Fla**‐**S_m_** were obtained by stirring a solution of the former in EtOAc in the presence of 2–4 equiv. of trifluoromethansulfonic acid for 24 h at room temperature and twofold recrystallization from EtOAc, which provided the mixed flavylium triflates **O_n_**‐**Fla**‐**S_m_** in 47–83 %, except for compounds **O_3_**‐**Fla**‐**S_1_**, **O_3_**‐**Fla**‐**S_3_**, which were isolated in lower yields of 19 % and 13 % respectively (Scheme 2). In general, yields of thioether‐substituted flavylium triflates **S_n_**‐**Fla**‐**S_m_** were lower (11 – 32 %) as compared to flavylium triflates **O_n_**‐**Fla**‐**S_m_** with mixed alkoxy and thioether chains due to tedious purification requiring multiple recrystallization.

Fortunately, we were able to confirm the structure of **O_1_**‐**V**‐**Fla**‐**S_1_** via single crystal Xray diffraction, revealing the absence of any S,S interaction in the solid state (for details see Supporting Information, Figure S1).^[23]^ Noteworthy, **O_1_**‐**V**‐**Fla**‐**S_1_** formed columnar stacks with antiparallel orientation of neighboring flavylium ions and resulting in π‐π stacking of the pyrylium cores with a distance of 3.50 Å, which is similar to the solid‐state structure of the known alkoxy derivative **O_1_**‐**V**‐**Fla**‐**O_1_**.^[17]^

### NMR Spectroscopic Studies on O_1_‐iV‐Fla‐S_3_

**Figure 2:**
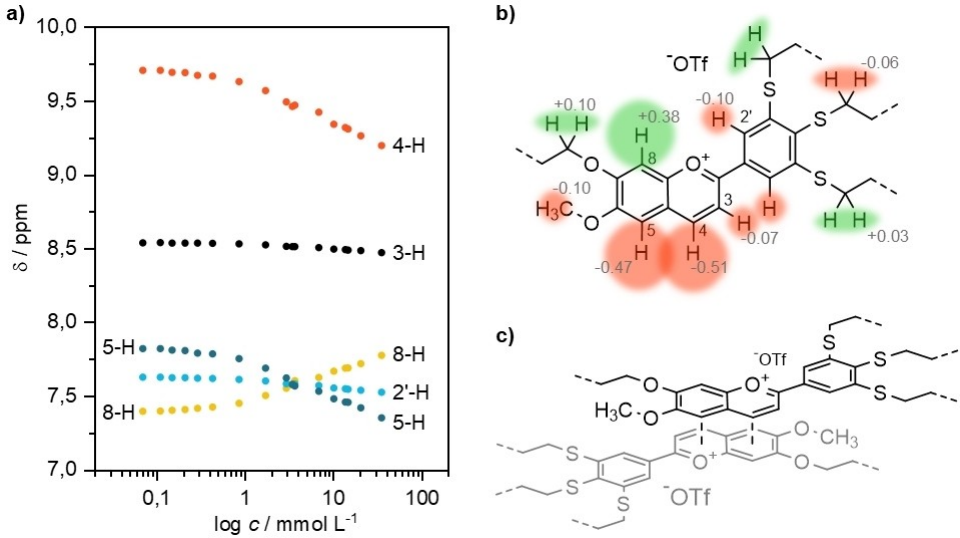
a) Chemical shift δ of the aromatic protons depending on the concentration. b) Change of the chemical shift Δδ from the lowest to the highest measured concentration (grey numbers, in ppm). c) Proposed packing model of a **O_1_**‐**iV**‐**Fla**‐**S_3_** dimer (alkyl chains are abbreviated by dashed lines).

Based on the known propensity of flavylium salts to form dimers and higher aggregates in solution resulting from strong intermolecular H‐bonds (in the presence of free OH groups), anion‐π, π‐π and electrostatic interactions^[15]^ standard 1D ^1^H NMR experiments for characterization of flavylium salts **O_n_**‐**Fla**‐**S_m_**, **S_n_**‐**Fla**‐**S_m_** were performed with dilute solutions (concentration≤3.4 mM) in CDCl_3_. All spectra were well resolved and showed sharp NMR signals except for the ^1^H NMR spectrum of compound **O_1_**‐**iV**‐**Fla**‐**S_3_**_,_ which exhibited significant line‐broadening effects, even at low concentrations. To understand the observed behavior of this amphiphilic thioether‐substituted flavylium salt, the aggregation of **O_1_**‐**iV**‐**Fla**‐**S_3_** in solution was studied by acquisition of concentration‐dependent ^1^H NMR spectra. With increasing concentration (concentration range: 0.07–14.7 mM) both up‐ and downfield shifts and broadening of some signals were observed (for details, see the Supporting Information, Figure S2), while other signals remained unaffected. The chemical shifts δ of diagnostic protons were plotted versus the concentration (Figure 2a), which revealed that 4‐H, 5‐H and 8‐H of the benzopyrylium scaffold, i. e. the AC rings showed strong shifts (Δδ>0.38 ppm) (Figure 2b), whereas only minor effects (Δδ<0.10 ppm) were observed for 3‐H, 2’‐H, OCH_2_, SCH_2_. From these differences in Δδ values and their relative signs (i. e. upfield or downfield shift) and the fact that an upfield (downfield) shift is caused by increased shielding (deshielding) of a proton, a packing model can be proposed. As outlined in Figure 2c benzopyrylium AC units form the center of vertical aggregates, so called H‐aggregates^[24]^ with an antiparallel orientation of two neighboring AC rings within the columnar aggregate. Thus, 5‐H and 4‐H experience an upfield shift when oligomerization takes place, since both come to rest over the partially, negatively charged oxonium ion and the C‐8 atom respectively (for charge distribution calculated via DFT, see Supporting Information, Figure S4). Additionally, by interacting with each other the 4‐H and 8‐H level out their strong differences in chemical shifts with increasing concentration. This packing model is supported by the X‐ray crystallographic data of **O_1_**‐**V**‐**Fla**‐**S_1_** (Figure S1) and agrees well with experimental data for the oxygen‐analogue **O_1_**‐**V**‐**Fla**‐**O_1_** ^[17]^ and other flavylium salts in solid state and solution.^[15]^

To get a more detailed insight into the aggregation of **O_1_**‐**iV**‐**Fla**‐**S_3_**_,_ diffusion ordered NMR spectra (DOSY) were carried out.^[25]^ Here, molecules are spatially labeled by use of field gradients. If they move during a following diffusion time, their position can be decoded by a second gradient. The measured signal is the integral over the whole sample volume, and it is attenuated depending on the diffusion coefficients of the analyte, the diffusion time and the gradient strength and length. The result is a “two‐dimensional” NMR spectrum in which ^1^H NMR spectrum of the sample is represented in F2 and the components are ordered in F1 with respect to their diffusion coefficients.

The experiments were performed with different concentrations of **O_1_**‐**iV**‐**Fla**‐**S_3_** (0.15–35.16 mM). The studies supported the presence of different oligomers in the given concentration range, since the signals of 4‐H showed four different diffusion coefficients in the processed DOSY spectrum: 0.15 mM (D=7.41 ⋅ 10^−10^ m^2^ s^−1^), 0.15–1.47 mM (D=6.46 ⋅ 10^−10^ m^2^ s^−1^), 0.29–2.94 mM (D=5.62 ⋅ 10^−10^ m^2^ s^−1^) and 3.67–14.7 mM (D=4.90 ⋅ 10^−10^ m^2^ s^−1^) presumably caused by aggregates with different sizes (Figure S3).

It should be noted that Freitas reported diffusion and self‐association constants of anthocyanins and flavylium/β‐cyclodextrin inclusion complexes obtained by DOSY,^[26]^ where the diffusion constants decrease with increasing aggregate size due to increasing hydrodynamic diameter. Although the experimentally determined diffusion constants were in a similar order of magnitude, direct comparison with flavylium salt **O_1_**‐**iV**‐**Fla**‐**S_3_** is not possible, because the applied solvents (D_2_O/DMSO‐D_6_ vs. CDCl_3_) and the investigated flavylium salts differ strongly in their polarity. Nevertheless, the NMR data suggests that the antiparallel columnar packing in the solid state is preserved to some extent in solution.

### Mesomorphic Properties of Thioether‐Substituted Flavylium ILCs

The mesomorphic properties were studied by optical polarizing microscopy (POM), differential scanning calorimetry (DSC) and X‐ray diffraction (XRD). The results of the DSC measurements are summarized in Table S2. All thioether‐substituted flavylium salts **O_n_**‐**Fla**‐**S_m_**, **S_n_**‐**Fla**‐**S_m_** were liquid crystalline except **O_1_**‐**iV**‐**Fla**‐**S_1_**, **S_3_**‐**Fla**‐**S_1_** and **O_3_**‐**Fla**‐**S_1_**. Furthermore, all liquid crystalline derivatives except **O_2_**‐**Fla**‐**S_2_** and **O_3_**‐**Fla**‐**S_3_** showed no signs of crystallization upon cooling but vitrified in a (metastable) glassy state preserving the mesophase textures.

According to our investigations, the thioether‐substituted flavylium ILCs could be grouped into two categories: (a) rod‐shaped (calamitic) ILCs with up to 2 alkoxy chains on the A ring and only one thioether on the B ring showing lamellar mesophases only, and (b) disk‐shaped (discotic) ILCs with 3–6 side chains in total and a minimum of 2 thioethers on the B ring displaying columnar mesophases.

**Figure 3:**
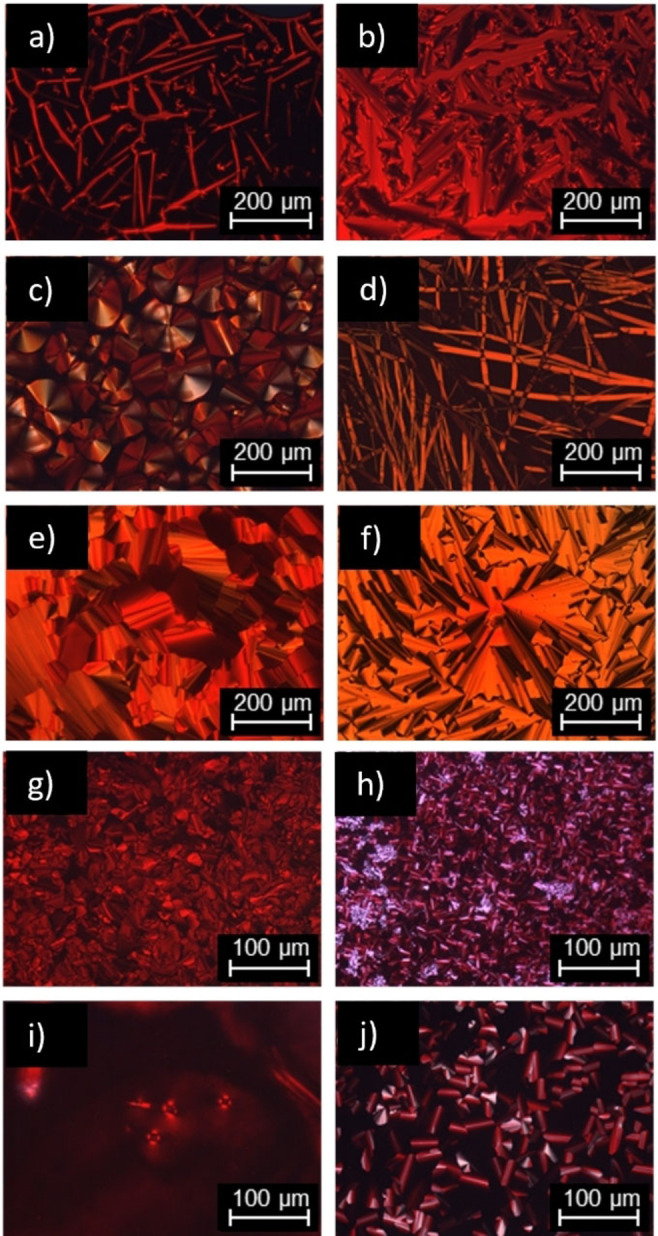
POM micrographs of **O_1_**‐**V**‐**Fla**‐**S_1_** a) at 193 °C and b) at 178 °C. POM micrographs of c) **O_1_**‐**iV**‐**Fla**‐**S_2_** at 147 °C, d) **O_1_**‐**iV**‐**Fla**‐**S_3_** at 150 °C, e) **O_2_**‐**Fla**‐**S_3_** at 44 °C and f) **O_3_**‐**Fla**‐**S_3_** at 146 °C. POM micrographs of g) **S_2_**‐**Fla**‐**S_1_** at 46 °C, h) **S_2_**‐**Fla**‐**S_2_** at 72 °C, i) **S_3_**‐**Fla**‐**S_1_** at −2 °C and j) **S_3_**‐**Fla**‐**S_3_** at 22 °C. All pictures were taken between crossed polarizers upon cooling from the isotropic phase with a cooling rate of 5 K min^−1^.

ILCs **O_1_**‐**V**‐**Fla**‐**S_1_** and **O_2_**‐**Fla**‐**S_1_** belonged to category (a). For example, upon cooling **O_1_**‐**V**‐**Fla**‐**S_1_** from the isotropic phase *Bâttonet* textures and large homeotropic areas were visible at 193 °C (Figure 3a), typical of a SmA phase. Upon further cooling, these textures changed into fan‐like textures indicating a columnar phase (Figure 3b). Similar behavior was observed for **O_2_**‐**Fla**‐**S_1_** (Figure S14a,b).

On the other hand, members of category (b) behaved differently under the POM. For example, **O_1_**‐**iV**‐**Fla**‐**S_2_** displayed pseudo‐focal conic textures at 147 °C upon cooling (Figure 3c), while **O_1_**‐**iV**‐**Fla**‐**S_3_** showed filament‐like textures at 150 °C (Figure 3d). Dendritic growth was detected for **O_2_**‐**Fla**‐**S_2_** at 142 °C (Figure S14c) and fan‐like textures with line defects and large homeotropic areas at 140 °C (Figure S14d). Fan‐like textures were also observed for **O_2_**‐**Fla**‐**S_3_** at 44 °C (Figure 3e) and **O_3_**‐**Fla**‐**S_3_** at 146 °C (Figure 3f). According to the POM investigations, the fully thioether‐substituted flavylium salts also belonged to category (b), showing pseudo‐focal conic textures, e. g. for **S_2_**‐**Fla**‐**S_2_** at 72 °C (Figure 3h) and **S_3_**‐**Fla**‐**S_2_** at 22 °C (Figure 3j) with or without line defects and homeotropic regions upon cooling. The violet appearance of the textures is a result of the strong intrinsic color of the flavylium salts. Fan‐shaped textures are common for smectic and columnar liquid crystals and further investigation via XRD was necessary to understand the mesomorphic behavior.

**Figure 4:**
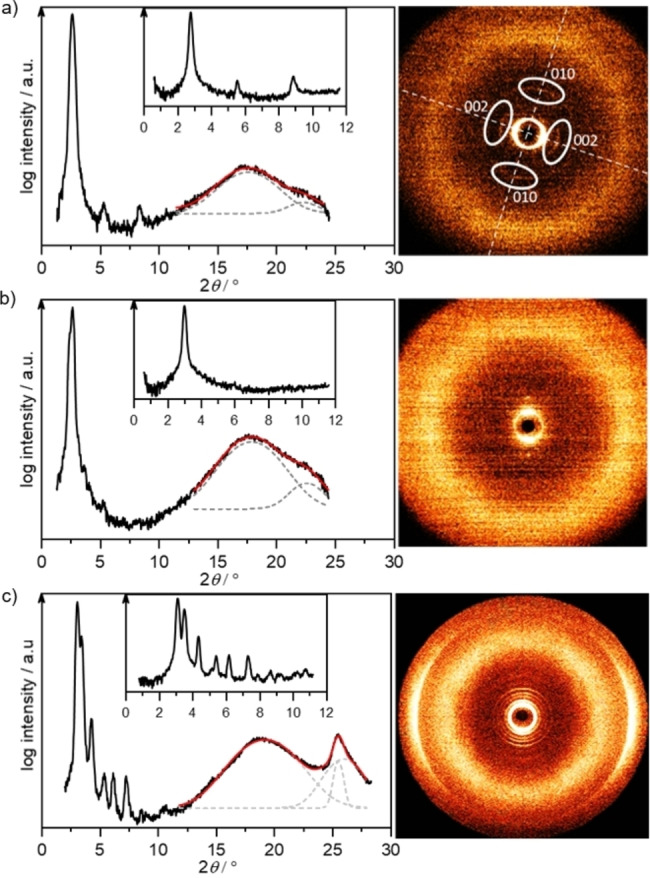
WAXS pattern (left) and diffractogram (right) and SAXS pattern (inset) of **O_1_**‐**V**‐**Fla**‐**S_1_** in a) the Lam_Col_ phase at 130 °C. b) the SmA phase at 190 °C. c) **O_1_**‐**V**‐**Fla**‐**S_2_** at 120 °C.

To fully assign the mesophase geometries, XRD studies were performed. All XRD data are summarized in Table 1. First, members of category (a) were examined. For **O_1_**‐**V**‐**Fla**‐**S_1_** (Figure 4a, at 130 °C) a sharp reflection in the small angle region at 31.65 Å and three reflections at 15.89 Å, 9.98 Å and 8.07 Å with smaller intensities were detected. These were assigned as (001), (002), (010) and (003) reflections of a lamellar columnar phase (Lam_Col_) due to the perpendicular orientation of the (010) reflection regarding the other small angle reflections (Figure 4a). In the wide‐angle section, a broad halo centered around 5.03 Å and an additional peak at 3.99 Å due to the π‐π interactions were detected in the WAXS (Figure 4a). Upon increasing the temperature to 190 °C a distinct sharp reflection in the small angle region (Figure 4b), i. e. the (001) reflection of SmA layers together with a broad halo around 4.91 Å and a small π‐π reflection peak at 3.94 Å were detected in the WAXS section. In a similar fashion, SAXS and WAXS data of **O_2_**‐**Fla**‐**S_1_** indicated a Lam_Col_ phase at lower temperatures and a SmA phase at higher temperatures. The layer spacing in both compounds is significantly smaller than the molecular length of about 42 Å, derived from the solid‐state structure. This is due to a partial interdigitation of the alkyl chains, caused by increased effective cross sections by the anion of the charged ionic core, as previously reported.^[17]^ The calamitic structure of the molecules belonging to category (a) is likely responsible for the formation of lamellar mesophases although a strong tendency for columnar stacking is already evident in the Lam_Col_ phases.

**Table 1:** XRD data of flavylium salts including the tilt angles α_alkyl_ and α_core_ for Col_ro_ phases.

Compound	Mesophase	Lattice parameters [Å]	*d* [Å]^[a]^	Miller indices
**O_1_‐V‐Fla‐S_1_**	SmA at 190 °C	–	29.53 4.91 3.94	(001) (halo) (π‐π)
	Lam_Col_ at 130 °C	–	31.65 15.89 (15.83) 9.98 8.07 (7.91) 5.03 3.99	(001) (002) (010) (003) (halo) (π‐π)
**O_2_‐Fla‐S_1_**	SmA at 120 °C	–	29.33 4.40	(001) (halo)
	Lam_Col_ at 90 °C	–	30.69 15.42 (15.34) 9.99 (10.23) 9.56 4.65	(001) (002) (003) (010) (halo)
**O_1_‐V‐Fla‐S_2_**	Col_ro_ at 120 °C *p2gg* α_alkyl_=36° α_core_=(12°)	*a*=50.3 *b*=34.3 *Z*=4	28.37 25.17 20.31 (20.30) 16.34 (16.25) 14.31 (14.19) 12.06 (12.00) 4.62 3.49 3.43	(11) (20) (21) (12) (22) (32) (halo) (anions) (π‐π)
**O_1_‐iV‐Fla‐S_2_**	Col_ro_ at 100 °C *p2gg* α_alkyl_=37° α_core_=(13°)	*a*=51.4 *b*=33.9 *Z*=4	28.31 25.71 20.46 (20.49) 16.21 (16.10) 14.29 (14.15) 12.12 (12.05) 11.04 (11.10) 10.28 (10.29) 4.58 3.49 3.42	(11) (20) (21) (12) (22) (32) (13) (42) (halo) (anions) (π‐π)
**O_1_‐iV‐Fla‐S_3_**	Col_ro_ at 100 °C *p2gg* α_alkyl_=37° α_core_=34° (37°)	*a*=53.3 *b*=33.4 *Z*=4	28.31 20.84 16.64 (16.70) 15.62 (15.69) 14.33 (14.16) 13.00 (13.33) 12.35 (12.38) 6.93 (6.98) 4.57 4.20 3.49	(11) (21) (02) (31) (22) (40) (41) (π‐π′) (halo) (anions) (π‐π)
**O_2_‐Fla‐S_2_**	Col_ho_ at 135 °C *p6 mm*	*a*=30.4 *Z*=2	26.35 4.66 3.56	(10) (halo) (π‐π)
	Col_ro_ at 100 °C *p2gg* α_alkyl_=45° α_core_=(10°)	*a*=48.6 *b*=37.6 *Z*=4	29.74 24.31 17.31 (17.53) 15.31 (14.88) 11.49 (11.57) 4.61 3.62 3.56	(11) (20) (12) (31) (41) (halo) (anions) (π‐π)
**O_2_‐Fla‐S_3_**	Col_ho_ at 100 °C	*a*=26.6 *Z*=1	23.03 13.51 (13.30) 4.46 3.62	(10) (11) (halo) (π‐π)
**O_3_‐Fla‐S_3_**	Col_ho_ at 100 °C	*a*=25.6 *Z*=1	22.04 12.90 (12.81) 11.07 (11.02) 4.51 3.90	(10) (11) (20) (halo) (π‐π)
**S_2_‐Fla‐S_1_**	Col_ho_ at 60 °C *p6 mm*	*a*=31.4 Z=2	27.15 15.82 (15.68) 13.71 (13.57) 4.62 3.62	(10) (11) (20) (halo) (π‐π)
**S_2_‐Fla‐S_2_**	Col_ho_ at 90 °C *p6 mm*	*a*=27.1 *Z*=1	23.48 13.70 (13.56) 4.51 3.55	(10) (11) (halo) (π‐π)
**S_3_‐Fla‐S_2_**	Col_ho_ at 100 °C *p6 mm*	*a*=26.9 *Z*=1	23.49 13.56 (13.47) 4.40 3.49	(10) (11) (halo) (π‐π)

[a] Calculated *d* values are given in parentheses.

As mentioned above, members of category (b) exhibit different textures compared to category (a) under the POM. A typical diffraction pattern of **O_1_**‐**V**‐**Fla**‐**S_2_** is shown in Figure 4c. In the small angle regime at 120 °C a sharp intense and 5 weaker reflections were observed, which were indexed as (11) (20) (21) (12) (22) (32) reflections of a Col_ro_ phase. In the wide‐angle regime, a broad halo centered around 4.62 Å and a second reflection at around 4.45 Å were detected. Fitting the wide angle regime (Figure 4c, dashed grey traces) revealed the later reflection consisting of the superposition of two reflections at 3.49 Å and 3.43 Å related to the anion distances (d_anion_) and π‐π interactions of the cationic cores (d_core_) respectively.^[17]^ In agreement with our previous report, alkyl chains and the cationic cores tilt out of the columnar plane in the Col_ro_ phase. While the alkyl tilt angle α_alkyl_ could be directly determined from the azimuthal angle of the halo of an oriented sample, the tilt angle of the core α_core_ was determined from the relation of d_anion_ and d_core_: α_core_=cos^−1^(d_core_/ d_anion_).^[17]^ For the Col_ro_ phase lattice parameters of *a*=50.3 Å, *b*=34.3 Å, *Z*=4 and tilt angles of α_alkyl_=36 ° and α_core_=12 ° were calculated. The corresponding isovanilin‐derived ILC **O_1_**‐**iV**‐**Fla**‐**S_2_** possessed similar phases and lattice parameters. For **O_1_**‐**iV**‐**Fla**‐**S_3_** the unit cell is slightly enlarged, despite the additional thioether side chain. However, the sterically more demanding B ring (as compared to **O_1_**‐**iV**‐**Fla**‐**S_2_**) is compensated by a significant increase in the tilt of the aromatic core of 34°.

For **O_2_**‐**Fla**‐**S_2_** a high temperature Col_ho_ phase with lattice parameter *a*=30.4 Å and *Z*=2 was deduced from the distinct (10) reflection in the SAXS, the broad halo around 4.66 Å and the small π‐π reflection at 3.56 Å. At lower temperatures a Col_ro_ phase was found again. For the higher homologues **O_2_**‐**Fla**‐**S_3_** and **O_3_**‐**Fla**‐**S_3_** only Col_ho_ phases with lattice parameters of *a*=26.6 Å and *a*=25.6 Å respectively and *Z*=1 were detected. It should be noted that lattice parameters of the hexagonal columnar mesophase decreased for these three ILCs despite the increased number of side chains. This can be rationalized as follows: In **O_2_**‐**Fla**‐**S_2_**, presumably two molecules form a discoid dimer whereas the increased B ring substitution in **O_2_**‐**Fla**‐**S_3_**, **O_3_**‐**Fla**‐**S_3_** suppresses dimer formation. Consequently, when comparing **O_2_**‐**Fla**‐**S_2_** and **O_2_**‐**Fla**‐**S_3_**, the lattice parameter decreases from a=30.4 Å to a=26.6 Å as the number of molecules in a unit cell decreases from 2 to 1. At the same time, the density decreases from ρ=1.31 g ⋅ cm^3^ to ρ=0.99 g ⋅ cm^3^ presumably due to a decreased space filling of the monomeric discoid **O_2_**‐**Fla**‐**S_3_** compared to the dimeric discoid in **O_3_**‐**Fla**‐**S_3_**. Compared to **O_2_**‐**Fla**‐**S_3_**, the additional alkoxy chain in **O_3_**‐**Fla**‐**S_3_** likely results in an increased space filling and thus in a smaller lattice parameter and an increased density of a=25.6 Å and ρ=1.13 g ⋅ cm^3^. Similar behavior was found for the series **S_2_**‐**Fla**‐**S_1_**, **S_2_**‐**Fla**‐**S_2_** and **S_3_**‐**Fla**‐**S_2_** (see Supporting Information, Table S3).

A similar packing behavior was found for fully thioether‐substituted ILCs **S_2_**‐**Fla**‐**S_1_**, **S_2_**‐**Fla**‐**S_2_**, **S_3_**‐**Fla**‐**S_2_**. XRD results of **S_2_**‐**Fla**‐**S_1_** confirmed a monotropic Col_ho_ phase. The lattice parameter of a=31.4 Å indicated, that two flavylium ions form a discoid. Assuming an anti‐parallel stacking of the flavylium cation in the Lam_Col_ phase, the increased steric demand of the sulfur atoms should disfavor this packing pattern, leading to reduced intermolecular interactions. As a result, rotation of the mesogens along the short molecular axis is less hindered, which leads to a more disc‐like appearance, thus promoting a columnar mesophase. Since 3 side chains are not sufficient to stabilize this phase, the mesogenic building block is a disk‐like dimer with 6 side chains in total. According to the XRD data of **S_2_**‐**Fla**‐**S_2_** and **S_3_**‐**Fla**‐**S_2_** a Col_ho_ phase containing one flavylium salt per discoid was assigned in both cases (Table 1). The decreased intermolecular interactions of **S_2_**‐**Fla**‐**S_2_** resulted in the Col_ho_ mesophase rather than a Col_ro_ mesophase as seen in **O_2_**‐**Fla**‐**O_2_** ^[17]^ and **O_2_**‐**Fla**‐**S_2_**, requiring stronger interactions to propagate the tilt of the columns. Evidently, molecules belonging to category exhibit columnar mesophases, which can be rationalized by the discotic geometry of derivatives with 2 or 3 thioethers tethered to the B ring.

**Figure 5:**
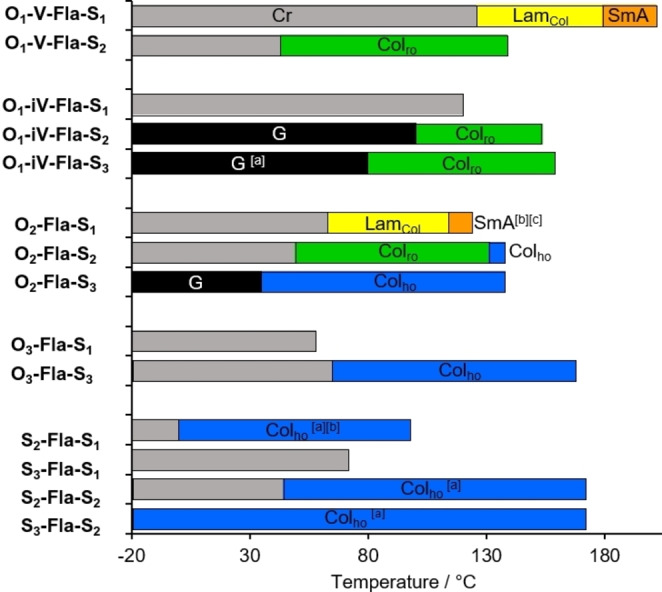
(Meso)phases and corresponding temperature ranges of the flavylium salts (temperatures determined from 1^st^ heating cycles, rate=10 K min^−1^): Cr (crystalline), G (glass), SmA (smectic A), Lam_Col_ (lamello‐columnar), Col_ro_ (ordered columnar rectangular), Col_ho_ (ordered columnar hexagonal);^[a]^ T_c_/ T_G_ determined by slow cooling via POM;^[b]^ monotropic behavior;^[c]^ SmA – Lam_Col_ transition determined from 1^st^ cooling cycle.

The XRD results revealed the influence of the molecular structure on the mesophase geometry. Whereas calamitic ILCs (type A) such as **O_1_**‐**V**‐**Fla**‐**S_1_**, **O_2_**‐**Fla**‐**S_1_** consisting of a linear structure formed SmA or Lam_Col_ phases, even a slightly bend structure as in **O_1_**‐**iV**‐**Fla**‐**S_1_** or unsymmetrical compounds such as **O_3_**‐**Fla**‐**S_1_** with large voids around the thioether side chain in the B ring supressed mesophase formation. On the other hand, with 2 thioethers at the B ring sufficient space filling could be achieved and Col_ro_ phases are favored for ILCs with up to 2 alkoxy chains at the A ring, e. g. **O_2_**‐**Fla**‐**S_2_**. The preference for Col_ro_ was also found, when the A ring carried only one alkoxy chain, as in **O_1_**‐**V**‐**Fla**‐**S_2_**, **O_1_**‐**iV**‐**Fla**‐**S_2_** and **O_1_**‐**iV**‐**Fla**‐**S_3_** respectively. In contrast, ILCs with either 3 thioethers at the B ring or ≥ 2 thioethers at the A ring displayed Col_h_ phases. Thus, the increased steric bulk of the thioethers as compared to alkoxy chains minimized the free volume and resulted in improved temperature range and stability of the columnar mesophase as evident by comparison of **O_2_**‐**Fla**‐**S_2_**, **O_2_**‐**Fla**‐**S_3_**, **S_2_**‐**Fla**‐**S_2_** in Figure 5. Members of discotic ILCs (type B) showed Col_ro_ phases mostly when alkoxy groups are grafted to the A ring, while Col_h_ phases were observed for derivatives with thioethers at the B ring.

The results of our DSC measurements and phase assignments were summarized in Figure 5. We want to point out that the investigated flavylium salts often vitrify in a (metastable) glass in agreement with our POM measurements and no crystallization or glass transition was detected in the cooling runs (see Supporting Information, Figure S5–S7 and Table S2). However, most derivatives exhibit a melting transition in the 1^st^ heating cycles indicating a crystalline phase at room temperature. Furthermore, derivatives with melting or clearing points above 150 °C gave no reproducible DSC data indicating thermal decomposition. We therefore decided to investigate transition temperatures from the 1^st^ heating cycles. The results of our DSC measurements and phase assignments were summarized in Figure 5 and allowed us to investigate the influence of substitution patterns on the mesomorphic properties.

Depending on the substitution pattern of the A ring, the following trends were observed. For flavylium salts **O_1_**‐**V**‐**Fla**‐**S_m_** with one alkoxy chain at the A ring melting and clearing temperatures decreased upon increasing the number of thioethers m at the B ring. Furthermore, the phase type changed from Lam_Col_, SmA for **O_1_**‐**V**‐**Fla**‐**S_1_** to Col_ro_ for **O_1_**‐**V**‐**Fla**‐**S_2_**.

In contrast, in the series of regioisomeric flavylium salts **O_1_**‐**iV**‐**Fla**‐**S_m_** clearing temperatures increased with larger m. Moreover, grafting of additional thioethers to the B ring induced a Col_ro_ phase in **O_1_**‐**iV**‐**Fla**‐**S_2_** and increased phase widths and stability in **O_1_**‐**V**‐**Fla**‐**S_3_**.

Flavylium salts **O_2_**‐**Fla**‐**S_m_** with two alkoxy groups at the A ring displayed higher clearing temperatures respectively, upon increasing m. Within the series phase types changed from Lam_Col_, SmA (for **O_2_**‐**Fla**‐**S_1_**) to Col_ro_, Col_ho_ (for **O_2_**‐**Fla**‐**S_2_**) and Col_ho_ (for **O_2_**‐**Fla**‐**S_3_**), while simultaneously temperature range and phase stability increased.

On the other hand, flavylium salts **O_3_**‐**Fla**‐**S_m_** carrying three alkoxy chains at the A ring showed a slight increase of melting transitions but a very pronounced increase of clearing temperatures upon increasing m, resulting in a broad Col_ho_ phase for **O_3_**‐**Fla**‐**S_3_** in contrast to the non‐mesomorphic **O_3_**‐**Fla**‐**S_1_**.

When the A ring carried thioether chains, e.g in flavylium salts **S_2_**‐**Fla**‐**S_m_**, increased melting and clearing temperatures were observed with larger m. Upon comparison of **S_2_**‐**Fla**‐**S_1_** with **S_2_**‐**Fla**‐**S_2_**, the two thioethers at both A and B ring in the latter case improved the overall molecular symmetry and thus increased the stability of the Col_ho_ phase. The beneficial effect of molecular symmetry and improved space filling on the mesophase stability is also visible for **S_3_**‐**Fla**‐**S_2_** with a broad Col_ho_ phase and its non‐mesomorphic counterpart **S_3_**‐**Fla**‐**S_1._**

Comparison of thioether‐substituted flavylium salts **O_n_**‐**Fla**‐**S_m_**, **S_n_**‐**Fla**‐**S_m_** with the corresponding alkoxy‐substituted analogues **O_n_**‐**Fla**‐**O_m_** ^[17]^ revealed only a small influence of the thioether/ether replacement on the phase types, but a more pronounced effect on the phase stability. Two extreme cases are **O_2_**‐**Fla**‐**O_1_**, **O_2_**‐**Fla**‐**S_1_** showing both lamellar phases and **O_3_**‐**Fla**‐**O_1_**, **O_3_**‐**Fla**‐**S_1_** where the columnar mesomorphism is completely lost in the sulfur case (for further details, see Supporting Information, Figures S12, S13). The number of side chains and substitution pattern determines the overall molecular geometry and thus the phase type (vide infra). With 2–3 side chains at the B ring columnar phases are exclusively formed. Unsymmetrical members with alkoxy chains at the A ring and thioethers at the B ring displayed Col_ro_ phases, while the correponding ILCs with thioethers at both A and B ring displayed Col_h_ phases. Thioether/ether replacement decreased the columnar phase stability, presumably due to less efficient packing of the bulkier thioethers and the absence of S−S interactions. However, for symmetrical derivatives **S_2_**‐**Fla**‐**S_2_**, **S_3_**‐**Fla**‐**S_2_** Col_h_ phases were much broader and more stable as compared to the O‐analogues.

### Linear Optical Properties of Thioether‐Substituted Flavylium ILCs

**Figure 6:**
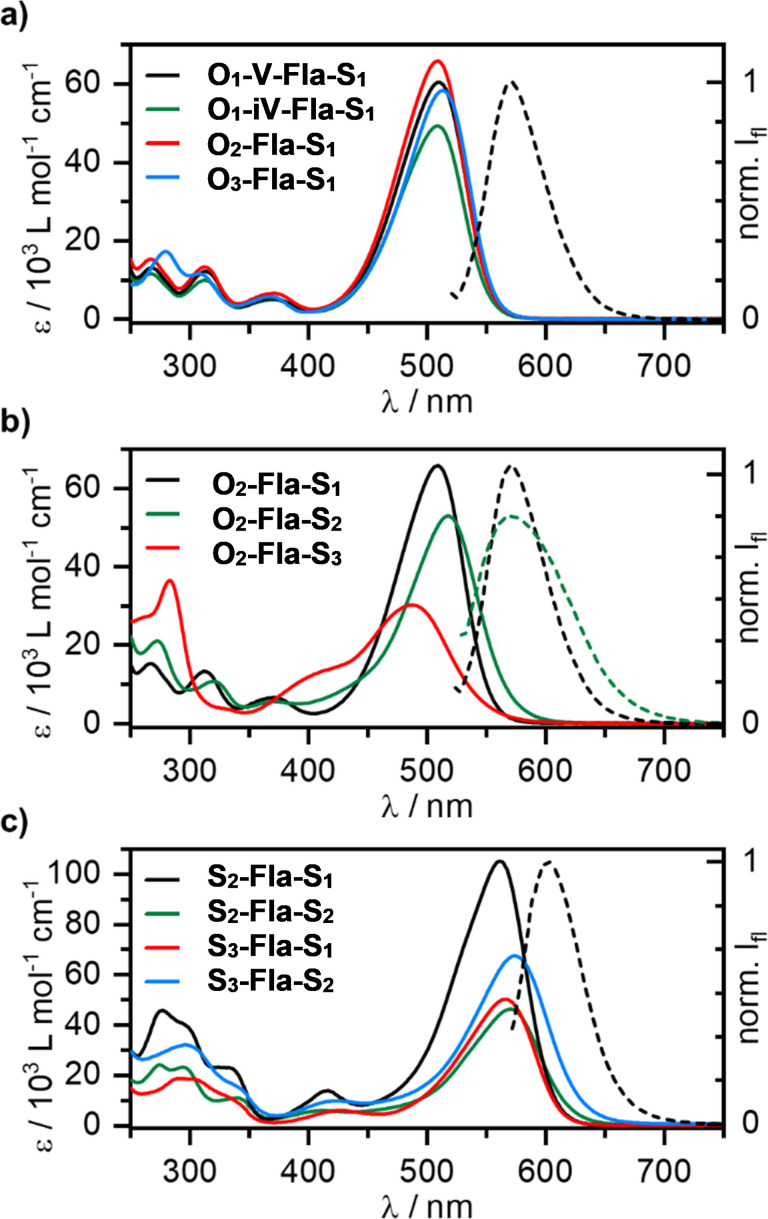
Absorption (solid lines) and, if applicable, emission (dashed lines) spectra of a) the series **O_n_**‐**Fla**‐**S_1_**, b) the series **O_2_**‐**Fla**‐**S_m_** and c) the series **S_n_**‐**Fla**‐**S_m_** recorded in CHCl_3_ (c=2 ⋅ 10^−5^ mol L^−1^, λ_ex_=510 nm).

All flavylium salts showed bright orange to purple colors in both solid state and solution and were thus investigated via UVVis and fluorescence spectroscopy in CHCl_3_ solution. The lowest energy absorption bands (λ_max_ and ϵ) are summarized in Table 2 and selected spectra are shown in Figure 6. For example, vanillin‐derivative **O_1_**‐**V**‐**Fla**‐**S_1_** decorated with one alkoxy chain at the A ring and one alkylsulfanyl chain at the B ring displayed three weak absorptions in the UV‐range (268 nm, 313 nm, 371 nm) and a strong absorption band in the visible region at 510 nm. Absorption maxima and extinction coefficients changed only marginally when the number of alkoxy chains n in the A ring increased in the series **O_1_**‐**V**‐**Fla**‐**S_1_**, **O_2_**‐**Fla**‐**S_1_** and **O_3_**‐**Fla**‐**S_1_** while keeping the B ring pattern constant (Figure 6a). The same trend was observed within the series of **O_n_**‐**Fla**‐**S_2_** and **O_n_**‐**Fla**‐**S_3_** (Figure S15).

**Table 2:** Photophysical data of thioether‐substituted flavylium salts **O_n_**‐**Fla**‐**S_m_** and **S_n_**‐**Fla**‐**S_m_**.

Compound	λ_max_ (ϵ) [nm (10^4^ L mol^−1^ cm^−1^)]^[a]^	λ_em_ (Φ) [nm (a.u.)]^[b]^
**O_1_‐V‐Fla‐S_1_**	510 (6.0)	571 (0.84)
**O_1_‐V‐Fla‐S_2_**	518 (5.0)	–
**O_1_‐iV‐Fla‐S_1_**	509 (4.9)	571 (‐)
**O_1_‐iV‐Fla‐S_2_**	518 (4.7)	–
**O_1_‐iV‐Fla‐S_3_**	487 (2.8)	–
**O_2_‐Fla‐S_1_**	510 (6.5)	571 (0.86)
**O_2_‐Fla‐S_2_**	518 (5.3)	–
**O_2_‐Fla‐S_3_**	487 (3.0)	–
**O_3_‐Fla‐S_1_**	513 (5.8)	–
**O_3_‐Fla‐S_3_**	488 (2.8)	–
**S_2_‐Fla‐S_1_**	552 (9.8)	602 (<0.01)
**S_2_‐Fla‐S_2_**	571 (4.6)	–
**S_3_‐Fla‐S_1_**	567 (5.0)	–
**S_3_‐Fla‐S_2_**	575 (6.7)	–

[a] measured in CHCl_3_ (c=2 ⋅ 10^−5^ L^−1^), ϵ determined from 5 different concentrations; [b] measured in degassed CHCl_3_ (c=2 ⋅ 10^−5^ L^−1^), λ_ex_=510 nm, Φ measured with a calibrated integration sphere.

In contrast, when the A ring substitution was kept constant and the number of thioethers at the B ring increased, a distinct change of the absorption properties was detected. Within the series **O_2_**‐**Fla**‐**S_1_**, **O_2_**‐**Fla**‐**S_2_**, **O_2_**‐**Fla**‐**S_3_** ϵ values decreased. A bathochromic shift of 34 cm^−1^ was observed for **O_2_**‐**Fla**‐**S_2_** as compared to **O_2_**‐**Fla**‐**S_1_** (λ_max_=509 nm), while **O_2_**‐**Fla**‐**S_3_** displayed a hypsochromic shift of 89 cm^−1^. The auxochromic effect of the thioethers was even more pronounced for the fully thioether‐functionalized compounds **S_n_**‐**Fla**‐**S_m_** resulting in bathochromically shifted absorption bands (552–575 nm) (Figure 6c) compared to the analogues **O_n_**‐**Fla**‐**S_m_**.

Albeit most flavylium salts prepared in this work being non‐emissive, the three derivatives **O_1_**‐**V**‐**Fla**‐**S_1_**, **O_1_**‐**iV**‐**Fla**‐**S_1_** (Figure 6a) and **O_2_**‐**Fla**‐**S_1_** (Figure 6b) exhibited bright emission (λ_em_=571 nm) upon irradiation in CHCl_3_ solution. This is in accordance with the related alkoxy flavylium salts **O_1_**‐**Fla**‐**O_1_**, where decoration of the B ring with more than one substituent increased the rotational freedom of the peripheral phenyl ring and thereby partially or fully inhibited emission.^[17]^

The slightly lower quantum yields of **O_1_**‐**V**‐**Fla**‐**S_1_**, **O_1_**‐**iV**‐**Fla**‐**S_1_** and **O_2_**‐**Fla**‐**S_1_** compared to their alkoxy analogues^[17]^ might be due to the decreased contribution of the flat Lewis structure with C=C double bond between A and C ring, resulting from the smaller electron‐donating +M effect and less efficient orbital interaction of the larger thioethers as compared to the alkoxy chains.^[17]^ It should be noted, that no evidence for side chains intercalating between the π‐systems, as recently reported by Douce for luminescent naphthalene imidazolium ILCs,^[27]^ could be found in our case. Additionally, the heavy atom effect^[28]^ of the sulfur might be responsible for the experimental results. The unfavourable influence of sulfur‐containing side chains on the luminescence was even more pronounced, when both A and B rings carried thioethers. For example, compound **S_2_**‐**Fla**‐**S_1_** (λ_em_=602 nm, Figure 10c) was the only emitting compound of the series **S_n_**‐**Fla**‐**S_m_** with a very low quantum yield <0.01. The rate of nonradiative relaxation usually increases with decreasing energy difference between S_1_ and S_0_ simply owing an improved overlap of higher vibrationally excited states in S_0_ and the zeroth vibrational state in S_1_ facilitating vibrational decay. This effect is commonly referred to as energy gap law^[29]^ and might, in addition to the even more pronounced heavy atom effect, be responsible for the (almost) non‐emissive behavior of the series **S_n_**‐**Fla**‐**S_m_**.

## Conclusions

In this work we have probed the self‐assembly of ILCs based on flavylium salts with alkoxy side chains on the A ring and thioethers on the B ring (**O_n_**‐**Fla**‐**S_m_** series), as well as thioethers on both A and B ring (**S_n_**‐**Fla**‐**S_m_** series) and compared them with the corresponding known O‐analogues **O_n_**‐**Fla**‐**O_m_**,^[17]^ carrying alkoxy chains on both A and B ring respectively.

In solution isovanillin‐derived ILC **O_1_**‐**iV**‐**Fla**‐**S_3_** with one alkoxy chain on the A ring and three thioethers on the B ring showed dynamic behaviour on the NMR time scale. DOSY experiments revealed four different diffusion constants (4.9–7.4 ⋅ 10^−10^ m^2^ s^−1^) caused by columnar aggregates of different sizes.

UV/Vis spectra of the flavylium salts in solution showed an auxochromic effect of thioethers in **O_n_**‐**Fla**‐**S_m_**, **S_n_**‐**Fla**‐**S_m_** as compared to O‐analogues **O_n_**‐**Fla**‐**O_m_**. Among the studied flavylium salts only **O_1_**‐**V**‐**Fla**‐**S_1_**, **O_1_**‐**iV**‐**Fla**‐**S_1_** and **O_2_**‐**Fla**‐**S_1_** with one thioether in the B ring displayed a bright green emission with high quantum yields (Φ=84 %), while **S_2_**‐**Fla**‐**S_1_** showed only a weak yellow emission (Φ<1 %). All other derivatives seem to favor conformations with a high degree of rotational freedom and thus a strong tendency for thermal deactivation. Presumably, formation of H‐aggregates also contributes to fluorescence quenching.

In the bulk state, POM, DSC and XRD provided insight into the liquid crystalline self‐assembly. In the **O_n_**‐**Fla**‐**S_m_** series the presence of thioethers in the B ring led to lower clearing points except for **O_1_**‐**iV**‐**Fla**‐**S_3_**, which has a higher clearing temperature as compared to the O‐analogue **O_1_**‐**iV**‐**Fla**‐**O_1_**.

For mixed O/S‐substituted ILCs, the mesophase type changed upon increasing the number of thioethers on the B ring from SmA, Lam_Col_ for calamitic ILCs carrying one thioether at the B ring and 1–2 alkoxy chains at the A ring, via Col_ro_ for discotic ILCs carrying 2–3 thioethers at the B ring and 1–2 alkoxy chains at the A ring to Col_ho_ for discotic ILCs carring up to 3 thioethers at both A and B rings. The replacement of O by S in the side chain resulted in most cases in decreased mesophase stability and temperature range without affecting the phase type, in particular for ILCs carrying alkoxy chains at the A ring and thioethers at the B ring. However, for fully thioether‐substituted derivatives **S_2_**‐**Fla**‐**S_1_**, **S_2_**‐**Fla**‐**S_2_**, **S_3_**‐**Fla**‐**S_2_** a significant stabilization of the Col_ho_ phase was observed. Moreover, for **S_2_**‐**Fla**‐**S_1_** the bulkiness of the thioethers induced a switch of the phase type from Lam_Col_ / SmA found for the alkoxy analogues **O_2_**‐**Fla**‐**S_1_**, **O_2_**‐**Fla**‐**O_1_** to Col_ho_.

Thus, the attachment of thioethers rather than alkoxy side chains to flavylium ILCs strongly favors columnar aggregates both in solution as well as in the bulk state. Future work must demonstrate whether this O/S replacement‐induced change of mesophase type can be applied to other classes of ILCs as well.

## Conflict of interest

There are no conflicts to declare.

1

## Supporting information

As a service to our authors and readers, this journal provides supporting information supplied by the authors. Such materials are peer reviewed and may be re‐organized for online delivery, but are not copy‐edited or typeset. Technical support issues arising from supporting information (other than missing files) should be addressed to the authors.

Supporting Information

## Data Availability

The data that supports the findings of this study are available in the supplementary material of this article.

## References

[1] N. V. Tabiryan , I.-C. Khoo , Handbook of Liquid Crystals, J. W. Goodby, P. J. Collings, T. Kato, C. Tschierske, H. Gleeson, P. Raynes (eds.), 2nd ed., Wiley-VCH, Weinheim, 2014, *vol. 8*, 453–473.

[2] T. Seki , N. Kawatsuki , M. Kondo , Handbook of Liquid Crystals, J. W. Goodby, P. J. Collings, T. Kato, C. Tschierske, H. Gleeson, P. Raynes (eds.), 2nd ed., Wiley-VCH, Weinheim, 2014, *vol. 8*, 539–579.

[3] S. J. Cowling , Handbook of Liquid Crystals, J. W. Goodby, P. J. Collings, T. Kato, C. Tschierske, H. Gleeson, P. Raynes (eds.), 2nd ed., Wiley-VCH, Weinheim, 2014, *vol. 8*, 581–625.

[4] K. Isoda , T. Yasuda , M. Funahashi , T. Kato , Handbook of Liquid Crystals, J. W. Goodby, P. J. Collings, T. Kato, C. Tschierske, H. Gleeson, P. Raynes (eds.), 2nd ed., Wiley-VCH, Weinheim, 2014, *vol. 8*, 709–725.

[5a] F. Würthner , K. Meerholz , Chem. A Eur. J. 2010, 16, 9366–9373; 10.1002/chem.201001153 20645353

[5b] F. Würthner , Acc. Chem. Res. 2016, 49, 868–876. 27064423 10.1021/acs.accounts.6b00042

[6] S. Riaz , M. Edgar , M. R. J. Elsegood , L. Horsburgh , S. J. Teat , T. G. Warwick , G. W. Weaver , Cryst. Growth Des. 2019, 19, 5237–5248.

[7] Y. Arakawa , Y. Sasaki , K. Igawa , H. Tsuji , New J. Chem. 2017, 41, 6514–6522.

[8] Q. Meng , X. H. Sun , Z. Lu , P. F. Xia , Z. Shi , D. Chen , M. S. Wong , S. Wakim , J. Lu , J. M. Baribeau , Y. Tao , Chem. A Eur. J. 2009, 15, 3474–3487. 10.1002/chem.200802470 19219863

[9] M. Mansueto , K. C. Kreß , S. Laschat , Tetrahedron 2014, 70, 6258–6264.

[10] S. T. Nestor , B. Heinrich , R. A. Sykora , X. Zhang , G. J. McManus , L. Douce , A. Mirjafari , Tetrahedron 2017, 73, 5456–5460.

[11] P. Espinet , E. García-Orodea , J. A. Miguel , Inorg. Chem. 2000, 39, 3645–3651. 11196828 10.1021/ic0001457

[12] J. Kirres , K. Schmitt , I. Wurzbach , F. Giesselmann , S. Ludwigs , M. Ringenberg , A. Ruff , A. Baro , S. Laschat , Org. Chem. Front. 2017, 4, 790–803.

[13] A. Jankowiak , D. Pociecha , J. Szczytko , H. Monobe , P. Kaszyński , Liq. Cryst. 2014, 41, 385–392.

[14a] N. J. Chothani , V. K. Akbari , P. S. Patel , K. C. Patel , Mol. Cryst. Liq. Cryst. 2016, 631, 31–46;

[14b] Y. Arakawa , S. Kang , H. Tsuji , J. Watanabe , G. I. Konishi , RSC Adv. 2016, 6, 16568–16574;

[14c] D. Węgłowska , P. Kula , J. Herman , RSC Adv. 2015, 6, 403–408;

[14d] B. He , J. Dai , D. Zherebetskyy , T. L. Chen , B. A. Zhang , S. J. Teat , Q. Zhang , L. Wang , Y. Liu , Chem. Sci. 2015, 6, 3180–3186; 29142688 10.1039/c5sc00304k PMC5657404

[14e] J. Mack , N. Kobayashi , M. J. Stillman , J. Inorg. Biochem. 2010, 104, 310–317; 19932508 10.1016/j.jinorgbio.2009.09.018

[14f] Y. Morita , R. Ono , H. Okamoto , K. Kasatani , Trans. Mater. Res. Soc. Japan 2009, 34, 455–458;

[14g] W. Su , Y. Zhang , C. Zhao , X. Li , J. Jiang , ChemPhysChem 2007, 8, 1857–1862; 17615615 10.1002/cphc.200700246

[14h] M. Hird , A. J. Seed , K. J. Toyne , J. W. Goodby , G. W. Gray , D. G. McDonnell , J. Mater. Chem. 1993, 3, 851–859;

[14i] M. Charton , J. Org. Chem. 1978, 43, 3995–4001.

[15a] F. Pina , M. J. Melo , C. A. T. Laia , A. J. Parola , J. C. Lima , Chem. Soc. Rev. 2012, 41, 869–908; 21842035 10.1039/c1cs15126f

[15b] F. Pina , V. Petrov , C. A. T. Laia , Dyes Pigm. 2012, 92, 877–889;

[15c] T. Goto , T. Kondo , Angew. Chem. Int. Ed. Engl. 1991, 30, 17–33.

[16a] B. A. Aguilar-Castillo , N. A. Sánchez-Bojorge , D. Chávez-Flores , A. A. Camacho-Dávila , E. Pasillas-Ornelas , L.-M. Rodríguez-Valdez , G. Zaragoza-Galán , J. Mol. Struct. 2018, 1155, 414–423;

[16b] G. Calogero , I. Citro , G. Di Marco , S. Caramori , L. Casarin , C. A. Bignozzi , J. Avó , A. Jorge Parola , F. Pina , Photochem. Photobiol. Sci. 2017, 16, 1400–1414; 28730204 10.1039/c7pp00039a

[16c] G. Calogero , A. Sinopoli , I. Citro , G. Di Marco , V. Petrov , A. M. Diniz , A. J. Parola , F. Pina , Photochem. Photobiol. Sci. 2013, 12, 883–894; 23467482 10.1039/c3pp25347c

[16d] F. J. Francis, P. C. Markakis, *Critical Reviews in Food Science and Nutrition Food Colorants: Anthocyanins*, **2009**; 10.1080/10408398909527503 2690857

[16e] F. Pina , M. J. Melo , M. Maestri , P. Passaniti , V. Balzani , J. Am. Chem. Soc. 2000, 122, 4496–4498;

[16f] N. J. Cherepy , G. P. Smestad , M. Grätzel , J. Z. Zhang , J. Phys. Chem. B 1997, 101, 9342–9351.

[17] R. Forschner , J. Knelles , K. Bader , W. Frey , C. Müller , A. Köhn , Y. Molard , F. Giesselmann , S. Laschat , Chem. Eur. J. 2019, 12966–12980. 31418972 10.1002/chem.201901975 PMC6856849

[18a] K. Goossens , K. Lava , C. W. Bielawski , K. Binnemans , Chem. Rev. 2016, 116, 4643–4807; 27088310 10.1021/cr400334b

[18b] Y. Ji , R. Shi , Y. Wang , G. Saielli , J. Phys. Chem. B 2013, 117, 1104–1109; 23305509 10.1021/jp310231f

[18c] S. Chen , S. H. Eichhorn , Isr. J. Chem. 2012, 52, 830–843;

[18d] L. Douce , J. M. Suisse , D. Guillon , A. Taubert , Liq. Cryst. 2011, 38, 1653–1661;

[18e] K. V. Axenov , S. Laschat , Materials (Basel). 2011, 4, 206–259; 28879986 10.3390/ma4010206 PMC5448481

[18f] K. Binnemans , Chem. Rev. 2005, 105, 4148–4204; 16277373 10.1021/cr0400919

[18g] S. K. Pal, S. Kumar, in *Biosensors Nanotechnology*, ed. A. Tiwani, A. P. F. Turner, Scrivener Publishing, Berkeley, MA, 2014, pp. 267–314;

[18h] N. Kapernaum , A. Lange , M. Ebert , M. A. Grunwald , C. Haege , S. Marino , A. Zens , A. Taubert , F. Giesselmann , S. Laschat , ChemPlusChem 2022, 87, e202100397. 10.1002/cplu.202100397 34931472

[19] A. Jankowiak , Z. Débska , J. Romański , P. Kaszyński , J. Sulfur Chem. 2012, 33, 1–7.

[20] S. Chassaing , M. Kueny-Stotz , G. Isorez , R. Brouillard , Eur. J. Org. Chem. 2007, 2007, 2438–2448.

[21] Q. Zheng , G. S. He , P. N. Prasad , J. Mater. Chem. 2005, 15, 579–587.

[22] S. Xia , L. Gan , K. Wang , Z. Li , D. Ma , J. Am. Chem. Soc. 2016, 138, 13493–13496. 27682010 10.1021/jacs.6b08114

[23] CCDC 2155920 (**O_1_ **-**V**-**Fla**-**S_1_ **) contains the supplementary crystallographic data for this paper. The data is provided free of charge by The Cambridge Crystallographic Data Centre. For comparison see CCDC 1913060 (**O_1_ **-**V**-**Fla**-**O_1_ **) reported in ref. [17].

[24] N. C. Maiti , S. Mazumdar , N. Periasamy , J. Phys. Chem. B 1998, 102, 1528–1538.

[25] D. H. Wu , A. Chen , C. S. Johnson , J. Magn. Reson. Ser. A 1995, 115, 260–264.

[26a] A. Fernandes , N. F. Brás , N. Mateus , V. De Freitas , New J. Chem. 2015, 39, 2602–2611;

[26b] A. Fernandes , N. F. Brás , N. Mateus , V. De Freitas , Langmuir 2014, 30, 8516–8527. 24991843 10.1021/la501879w

[27a] N. del Giudice , M. L'Her , E. Scrafton , Y. Atoini , G. Gentile , B. Heinrich , R. Berthiot , A. Aliprandi , L. Douce , Eur. J. Org. Chem. 2021, 2091–2098;

[27b] M. L'Her , Y. Atoini , J. Fouchet , B. Heinrich , N. Del-Gioudice , E. Scrafton , E. Bordes , L. Karmazin , L. Charbonniere , L. De Cola , L. Douce , New J. Chem. 2020, 44, 2669–2669;

[27c] J. Fouchet , B. Heinrich , M. L'Her , E. Voirin , L. Karmazin , C. Bailly , R. Welter , A. Mirjafari , L. Douce , New J. Chem. 2018, 42, 10421–10431.

[28] K. Michael , Discuss. Faraday Soc. 1950, 9, 14–19.

[29a] Y.-C. Wei , S. F. Wang , Y. Hu , L.-S. Liao , D.-G. Chen , K.-H. Chang , C.-W. Wang , S.-H. Liu , W.-H. Chan , J.-L. Liao , et al., Nat. Photonics 2020, 14, 570–577;

[29b] S. H. Lin , J. Chem. Phys. 1970, 53, 3766–3767.

