# Supplementary material for: Chasing Self‐Assembly of Thioether‐Substituted Flavylium Salts in Solution and Bulk State
Source: Chemphyschem. 2022 May 17;23(13):e202200154. doi: 10.1002/cphc.202200154 (PMC9400860; doi:10.1002/cphc.202200154)
Supplement: Supplementary file 1 — Supporting Information [file CPHC-23-0-s001.pdf]

ChemPhysChem

Supporting Information

Chasing Self-Assembly of Thioether-Substituted Flavylium Salts in Solution and Bulk State

Julius A. Knöller, Robert Forschner, Wolfgang Frey, Johannes Lang, Angelika Baro, Anna Zens, Yann Molard, Frank Giesselmann, Birgit Claasen, and Sabine Laschat*

Supporting Information

Table of contents:

1 General Information 2

2 Synthetic Procedures 4

3 Solid State Structure of **O_1-V-Fla-S_1** 22

4 NMR Experiments (**O_1-iV-Fla-S_3**) 25

5 DSC, X-Ray Scattering and UV-VIS Experiments 28

6 References 39

7 ^{1}H and ^{13}C NMR spectra 41

1 General Information

All chemicals were, unless otherwise stated, provided by Sigma Aldrich and used without further purification. Trifluormethanesulfonic acid (HOTf) was used in the *ReagentPlus*® (≥99%) grade from Sigma Aldrich and the acid was exclusively handled in glassware to avoid contamination by metals. Dry THF was obtained by distillation over potassium. DMSO was dried over activated 4Å molecular sieve and degassed prior to use. The eluents for chromatography, petroleum ether (PE, low-boiling point) and ethyl acetate (EtOAc) were distilled prior to use. ^{1}H NMR/ ^{13}C NMR spectra were measured in $CDCl_3$ on Bruker Avance 300, Avance 400, Avance 500, and Avance 700 spectrometers at 300/ 76 MHz, 400 MHz/ 101 MHz, 500 MHz/ 126 MHz and 700 MHz/ 176 MHz, respectively. Chemical shifts δ were given in parts per million (ppm) and were referenced to tetramethylsilane (TMS, δ_{TMS} = 0 ppm). Atom labelling can differ from the IUPAC nomenclature to facilitate better comparability. Assignment of the ^{1}H and ^{13}C NMR signals was done via COSY, HSQC, HMBC, and NOESY measurements. FT-IR spectra were measured on a Bruker Vektor 22 with a MKII Golden Gate Single Reflection Diamond ATR. Absorption bands were rounded to integer wavenumbers / cm^{-1} and the absorption intensities were classified as follows: w (weak), m (medium), s (strong), vs (very strong). Mass spectra (MS) and high-resolution mass spectra (HRMS) were measured by electrospray ionisation (ESI) on a Bruker MicrOTOF-Q spectrometer. CHN Analysis was performed on an Elemental Analyzer Model 1106 from Carlo Erba Strumentazione. For thin layer chromatography, silica gel 60 F254 glass plates (layer thickness of 0.25 mm) on aluminium (pore size 60 Å) from Merck were used. Column chromatography was performed with the indicated solvent as mobile phase on silica gel (Fluka, particle diameter of 40 – 60 µm) as stationary phase.Polarizing optical microscopy (POM) was performed on a polarising optical microscope Olympus BX 50, equipped with a Linkam LTS heating stage. Temperature regulation was carried out with the control units TP93 and LNP from Linkam (ΔT = ± 1 K). Photographs were recorded with a digital camera ColorView from Soft Imaging System using the software analySIS. [1] For differential scanning calorimetry, a DSC822e from Mettler Toledo was employed. The substances were analysed in 40 µL sealed aluminium pans. Heating and cooling rates of 5 K min^{-1} were employed. Phase transition temperatures and enthalpies were determined by onset values using the software STARe 7.01. [2] Temperature dependent wide angle x-ray diffraction (WAXS) and small angle x-ray diffraction (SAXS) was performed on a Bruker AXS Nanostar C with a ceramic tube generator (1500 W) having cross-coupled Goebel mirrors providing monochromatic Cu K_α radiation (1.5405 Å). Diffraction patterns were recorded with Bruker HI-STAR or VÅNTEC 500 detectors. Calibration was carried out using the diffraction pattern of silver behenate at room temperature. The compounds were examined in sealed glass capillaries from Hilgenberg

GmbH (external diameter of 0.7 mm, wall thickness 0.01 mm). Fibre samples were obtained by extrusion of the material at room temperature. Measured values were analysed with the software SAXS[3] from Bruker. The diffraction patterns were further processed using the software Datasqueeze[4], Origin[5] and LCDiXray[6]. UV-Vis and emission measurements were performed in $CHCl_3$ solution (spectrophotometric grade, $c = 2 \cdot 10^{-5}$ mol L^{-1}, $\lambda_{ex} = 510$ nm) on Perkin Elmer Lambda 35 and Perkin Elmer LS 55 spectrometers, respectively. Quantum Yields were determined on a Hamamatsu C9920-03 absolute quantum yield spectrometer equipped with a 150 W xenon lamp, monochromator and PMA-12 detector. Computation was performed using the Gaussian16[7] suite of programs. Geometries were optimized on B3LYP[8]/AUG-cc-pVTZ[9] level of theory and charge distributions were determined employing natural population analysis (NAO)[10].

2 Synthetic Procedures

The thioether substituted aryl bromides **7b,c** and corresponding arylaldehydes **8b,c** were prepared according to Jankowiak.[11] BHMPO was prepared according to Ma.[12] The phenols **11d-e** were obtained as previously described from the aldehydes **8d-g**.[13]

General procedure for the syntheses of ethynylalcohols (9a-f) by Grignard reaction (GP 1)

In a dried Schlenk flask the arylaldehyde **8a** (16.6 mmol) was dissolved in abs. THF (30 mL) and cooled to 0 °C. To the solution was added ethynylmagnesium bromide solution (20.1 mmol, 0.5 M in THF) slowly and the reaction was stirred for additional 3 h at room temperature. After the addition of saturated NH_4Cl solution THF was removed under reduced pressure and the aqueous phase was extracted with EtOAc (3 x 50 mL). After drying over $MgSO_4$ and the solvent was removed under reduced pressure and the product was used without further purification.[14]

General procedure for the syntheses of ethynylketones (10a-c) by oxidation of ethylnylalcohols (9a-c) (GP 2)

The ethynylalcohol **9a** (4.64 g, 14.0 mmol) was dissolved in EtOAc (120 mL) and IBX [15] (7.82 g, 27.9 mmol) was added at room temperature. The suspension was stirred at 80 °C for 18 h. After cooling to room temperature, the mixture was filtrated over Celite® and the filter cake was washed with two additional portions of EtOAc. After evaporation of the solvent the crude product was purified by column chromatography (PE/EE = 20 : 1), yielding the product as yellow solid (3.02 g, 9.13 mmol, 66 %).[14]

General procedure for the syntheses of flavylium salts (O_n-Fla-S_m) (GP 3)

To a solution of phenol **11b-g** (98 mg, 318 µmol for **O_1-V-Fla-S_1**) and ethynylketone **10a-c** (105 mg, 318 µmol for **O_1-V-Fla-S_1**) in EtOAc (10 mL) an excess of triflic acid (600 µmol) was added and stirred for 18 h at room temperature. The product precipitated and was recrystallized directly from the reaction solution. For products with high solubility at room temperature the reaction mixture was stored at 4 °C or -18 °C until the product precipitated. Then 2 mL of absolute EtOH (*p.a.* grade) was added, the mixture was filtered and the obtained solid was washed with EtOH. The obtained product was dried in a desiccator over phosphorous pentoxide for 3 days.[14]

4-(Dodecylthio)benzaldehyde (8a)

According to Prasad[16] *p*-fluorobenzaldehyde **5a** (3.00 g, 24.2 mmol) and 1-dodecanethiol (4.89 g, 24.2 mmol) were dissolved in dry DMSO (45 mL). Na_2CO_3 (7.69 g, 72.5 mmol, dried under vacuum) was added to the solution and stirred at 160 °C for one day. After addition of demin. Water (200 mL)

the aqueous phase was extracted with CH_2Cl_2 (3 x 100 mL). The combined organic phases were dried over $MgSO_4$ and the solvent was removed under reduced pressure. The residue was purified by column chromatography (PE / EtOAc = 75 : 1 to 50 : 1) giving the product **4a** as a yellow solid (6.94 g, 22.6 mmol, 94 %).

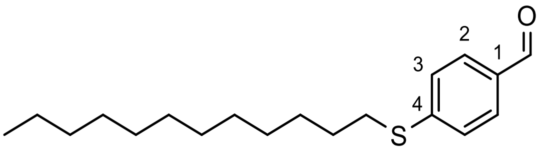

8a
$C_{19}H_{30}OS$
(306.51)

^{1}H NMR (500 MHz, $CDCl_3$): δ = 0.88 (t, J = 6.9 Hz, 3H, CH_3), 1.17–1.39 (m, 16H, CH_2), 1.66–1.76 (m, 4H, $SCH_2CH_2CH_2$), 2.96–3.04 (m, 2H SCH_2), 7.32–7.37 (m, 2H, 3-H), 7.68–7.82 (m, 2H, 2-H), 9.92 (s, 1H, CHO) ppm. ^{13}C NMR (126 MHz, $CDCl_3$) δ = 14.12 (CH_3), 22.69, 28.64, 28.91, 29.14, 29.34, 29.47, 29.56, 29.62, 29.64, 31.81 (CH_2), 31.91 (SCH_2), 126.27 (C-3**)**, 129.99 (C-2**)**, 133.09 (C-1**)**, 147.16 (C-4**)**, 191.18 (CHO) ppm. FT-IR (ATR): $\tilde{\nu}$ = 2921 (s), 2851 (m), 2730 (w), 1696 (s), 1672 (m), 1590 (s), 1561 (m), 1489 (w), 1465 (w), 1386 (w), 1304 (w), 1214 (m), 1169 (m), 1087 (s), 1011 (w), 836 (s), 810 (s), 721 (w), 698 (w), 629 (w), 541 (w), 484 (m) cm^{-1}. MS (ESI): *m/z* = 329.19 [M + Na$^+$], 361.22 [M + 2O + Na$^+$], 479.28, 697.53 [2M + K$^+$]. HRMS (ESI): for $C_{19}H_{30}OSNa$ calc.: 329.1910 found: 329.1900.

1-(4-(Dodecylthio)phenyl)prop-2-yn-1-ol (9a)

Synthesis according to GP 1: **8a** (5.08 g, 16.6 mmol), ethynylmagnesium bromide (0.5 M in THF, 40.2 mL, 20.1 mmol), yield: 5.46 g, 16.4 mmol, 99%, yellow solid.

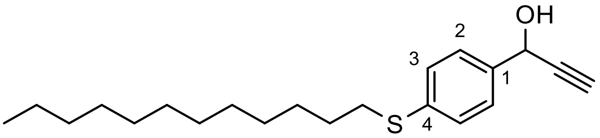

9a
$C_{21}H_{32}OS$
(332.55)

^{1}H NMR (500 MHz, $CDCl_3$): δ = 0.88 (t, J = 6.9 Hz, 3H, CH_3), 1.20–1.45 (m, 18H, CH_2), 1.60–1.68 (m, 2H, SCH_2CH_2), 2.21 (d, J = 5.9 Hz, 1H, OH), 2.66 (d, J = 2.2 Hz, 1H, CCH), 2.88–2.95 (m, 2H, SCH_2), 5.40–5.45 (m, 1H, C*H*OH), 7.31 (d, J = 8.3 Hz, 2H, 3-H), 7.45 (d, J = 8.3 Hz, 2H), 2-H) ppm. ^{13}C NMR (126 MHz, $CDCl_3$) δ = 14.13 (CH_3), 22.69, 28.86, 29.07, 29.17, 29.35, 29.51, 29.58, 29.63, 29.64, 31.91 (CH_2), 33.34 (SCH_2), 64.05 (CHOH), 74.89 (C*C*H), 83.32 (*C*CH), 127.12 (C-2), 128.62 (C-3) , 137.28 (C-1), 137.86 (C-4) ppm. FT-IR (ATR): $\tilde{\nu}$ = 3404 (w), 3286 (m), 2954 (w), 2916 (s), 2850 (s), 1597 (w), 1493 (w), 1463 (w), 1402 (w), 1289 (w), 1265 (w), 1093 (w),

1022 (m), 950 (w), 907 (w), 845 (w), 786 (m), 727 (m), 676 (m), 586 (w), 519 (w), 498 (w), 433 (w) cm^{-1}. MS (EI): *m/z* = 332.2 [M^+]. HRMS (ESI): for $[C_{21}H_{32}OS]^+$ calc.: 332.2174 found: 332.2158.

1-(3,4-Bis(dodecylthio)phenyl)prop-2-yn-1-ol (9b)

Synthesis according to GP 1: **8b** (2.52 g, 4.97 mmol), ethynylmagnesium bromide (0.5 M in THF, 12.1 mL, 6.05 mmol), yield: 2.54 g, 4.77 mmol, 96 %, yellow solid.

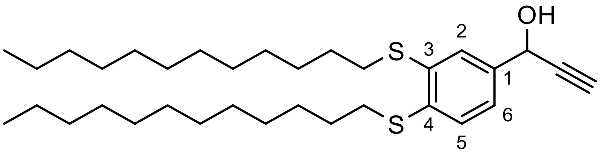

9b
$C_{33}H_{56}OS_2$
(532.93)

^{1}H NMR (500 MHz, $CDCl_3$): δ = 0.81–0.97 (m, 6H, CH_3), 1.17–1.37 (m, 32H), 1.39–1.51 (m, 4H, $SCH_2CH_2CH_2$), 1.62–1.74 (m, 4H, SCH_2CH_2), 2.21 (m, 1H, OH), 2.68 (d, *J* = 1.6 Hz, 1H, CCH), 2.85–3.00 (m, 4H, SCH_2), 5.43 (d, *J* = 1.6 Hz, 1H, CHOH), 7.25 (d, *J* = 7.9 Hz, 1H, 5-H), 7.28–7.32 (m, 1H, 6-H), 7.44 (s, 1H, 2-H) ppm. ^{13}C NMR (126 MHz, $CDCl_3$) δ = 14.13 (CH_3), 22.70, 28.79, 28.82, 29.00, 29.22, 29.24, 29.36, 29.52, 29.53, 29.61, 29.64, 29.66, 31.92 (CH_2), 33.22, 33.24 (SCH_2), 64.03 (CHOH), 75.01 (C*C*H), 83.20 (*C*CH), 124.10 (C-6), 126.45 (C-2), 128.35 (C-5), 137.68, 137.70 (C-3, C-4, C-1) ppm. FT-IR (ATR): $\tilde{\nu}$ = 3308 (s), 2920 (s), 2851 (w), 1586 (w), 1456 (m), 1378 (w), 1257 (w), 1193 (w), 1113 (w), 1039 (w), 960 (w), 851 (w), 722 (w), 656 (w), 631 (w), 518 (w), 448 cm^{-1}. MS (ESI): *m/z* = 555.36 [M + Na^+], 1087.74 [2M + Na^+]. HRMS (ESI): for $C_{33}H_{56}OS_2Na$ calc.: 555.3665 found: 555.3647.

1-(3,4,5-Tris(dodecylthio)phenyl)prop-2-yn-1-ol (9c)

Synthesis according to GP 1: **8c** (1.56 g, 2.21 mmol), ethynylmagnesium bromide (0.5 M in THF, 5.4 mL, 2.7 mmol), purification: column chromatography (PE / EtOAc = 15 : 1), yield: 0.75 g, 1.02 mmol, 46%, yellow solid.

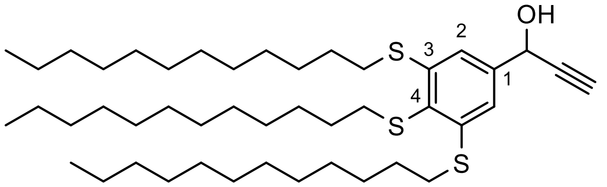

9c
$C_{45}H_{80}OS_3$
(733.31)

^{1}H NMR (500 MHz, $CDCl_3$): δ = 0.80–0.94 (m, 9H, CH_3), 1.17–1.53 (m, 58H, CH_2), 1.53–1.65 (m, 2H, 4-SCH_2CH_2), 1.67–1.80 (m, 4H, 3-SCH_2CH_2), 2.22 (m, 1H, OH), 2.69 (d, *J* = 2.22 Hz,

1H, C*H*OH), 2.83 (t, *J* = 7.41 Hz, 2H, 4-SCH_2), 2.89 (t, *J* = 7.4 Hz, 4H, 3-SCH_2), 5.43 (s, 1H, CCH), 7.07 (s, 2H, 2-H) ppm. ^{13}C NMR (126 MHz, $CDCl_3$) δ = 14.13 (CH_3), 22.70, 28.28, 28.99, 29.21, 29.27, 29.29, 29.36, 29.38, 29.55, 29.59, 29.63, 29.65, 29.68, 29.71, 31.93, 31.94 (CH_2), 32.26 (3-SCH_2), 34.99 (4-SCH_2), 64.32 (CHOH), 75.22 (C*C*H), 83.02 (*C*CH), 118.26 (C-2), 128.24 (C-4), 140.77 (C-1), 146.98 (C-3) ppm. FT-IR (ATR): $\tilde{\nu}$ = 3310 (s), 2954 (s), 2920 (w), 2851 (w), 2119 (w), 2018 (w), 1981 (w), 1570 (w), 1540 (w), 1465 (w), 1393 (w), 1298 (w), 1265 (w), 1194 (w), 1042 (w), 1022 (w), 981 (w), 838 (w), 795 (w), 721 (w), 656 (w), 630 (w) cm^{-1}. MS (ESI): *m/z* = 731.53 $[M–H]^-$, HRMS (ESI): for $C_{45}H_{79}OS_3$ calc: 731.5288 found: 731.5382.

1-(4-(Dodecylthio)phenyl)prop-2-yn-1-one (10a)

Synthesis according to GP 2: **9a** (4.64 g, 14.0 mmol), IBX (7.82 g, 27.9 mmol), yield: 3.02 g, 9.13 mmol, 66%, yellow solid.

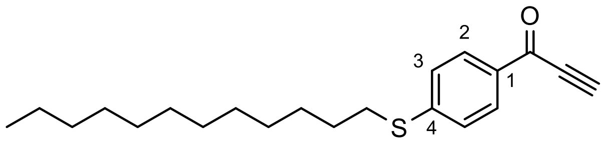

10a
$C_{21}H_{30}OS$
(330.53)

^{1}H NMR (500 MHz, $CDCl_3$): δ = 0.88 (t, *J* = 7.0 Hz, 3H, CH_3), 1.18–1.38 (m, 16H CH_2), 1.41–1.50 (m, 2H, $SCH_2CH_2CH_2$), 1.66–1.75 (m, 2H, SCH_2CH_2), 3.00 (t, *J* = 7.8 Hz, 2H, SCH_2), 3.39 (s, 1H, CCH), 7.30 (d, *J* = 8.5 Hz, 2H, 2-H), 8.04 (d, *J* = 8.5 Hz, 2H, 3-H) ppm. ^{13}C NMR (126 MHz, $CDCl_3$) δ = 14.13 (CH_3), 22.69, 28.62, 28.91, 29.14, 29.34, 29.47, 29.56, 29.62, 29,63 (CH_2), 31.73 (SCH_2), 31.91 (CH_2), 80.31 (C*C*H), 125.80 (C-2), 130.01 (C-1), 132.79, 147.58 (C-3, C-4), 176.27 (CO) ppm. FT-IR (ATR): $\tilde{\nu}$ = 3277 (m), 2954 (w), 2915 (s), 2871 (w), 2848 (s), 2103 (s), 1704 (w), 1648 (s), 1589 (s), 1558 (w), 1488 (w), 1472 (w), 1462 (m), 1399 (w), 1383 (w), 1365 (w), 1344 (w), 1316 (w), 1284 (w), 1261 (s), 1183 (m), 1116 (w), 1092 (s), 1023 (m), 1005 (s), 970 (w), 909 (w), 834 (m), 743 (s), 730 (m), 718 (m), 677 (s), 531 (m), 479 (m), 442 (w), 415 (w) cm^{-1}. MS (EI): *m/z* = 330.2 $[M^+]$. HRMS (EI): for $C_{21}H_{30}OS$ calc.: 330.2017 found: 330.2016.

1-(3,4-Bis(dodecylthio)phenyl)prop-2-in-1-one (10b)

Synthesis according to GP 2: **9b** (2.39 g, 4.48 mmol), IBX (2.51 g, 8.97 mmol), yield: 2.16 g, 4.07 mmol, 91%, yellow solid.

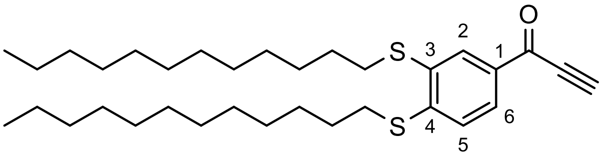

10b
$C_{33}H_{54}OS_2$
(530.91)

^{1}H NMR (700 MHz, $CDCl_3$): δ = 0.85–0.90 (m, 6H, CH_3), 1.21–1.37 (m, 32H, CH_2), 1.41–1.51 (m, 4H, $SCH_2CH_2CH_2$), 1.64–1.71 (m, 2H, SCH_2CH_2), 1.71–1.78 (m, 2H, SCH_2CH_2), 2.95–3.02 (m, 4H, SCH_2), 3.41 (s, 1H, CCH), 7.24 (d, J = 8.3 Hz, 1H, 5-H), 7.92 (dd, J = 8.3 Hz, 1.9 Hz, 1H, 6-H), 8.02 (d, J = 1.9 Hz, 1H, 2-H) ppm. ^{13}C NMR (176 MHz, $CDCl_3$) δ = 14.13 (CH_3), 22.70, 28.31, 28.81, 28.91, 29.06, 29.18, 29.20, 29.35, 29.48, 29.51, 29.58, 29.59, 29.63, 29.66, 31.92 (CH_2), 32.33, 33.54 (SCH_2), 80.27 (*C*CH), 80.45 (C*C*H), 124.43 (C-1), 127.59 (C-5), 129.19 (C-6), 132.82 (C-2), 135.56, 148.06 (C-4, C-3), 176.11 (CHO) ppm. FT-IR (ATR): $\tilde{\nu}$ = 3251 (s), 2921 (s), 2851 (s), 2097 (m), 2027 (w), 2011 (w), 1964 (w), 1645 (s), 1573 (s), 1546 (w), 1457 (m), 1377 (w), 1272 (m), 1221 (s), 1107 (w), 1039 (m), 1021 (w), 890 (w), 805 (w), 744 (m), 721 (w), 686 (w), 652 (w), 556 (w), 509 (w), 447 (w) cm^{-1}. MS (ESI): *m/z* = 553.35 [M + Na^{+}], 1083.71 [2M + Na^{+}]. HRMS (ESI): for $C_{33}H_{54}OS_2Na$ calc.: 553.3508 found: 553.3510.

1-(3,4,5-Tris(dodecylthio)phenyl)prop-2-yn-1-one (10c)

Synthesis according to GP 2: **9c** (0.75 g, 1.02 mmol), IBX (0.57 g, 2.03 mmol), yield: 0.69 g, 0.94 mmol, 93%, yellow solid.

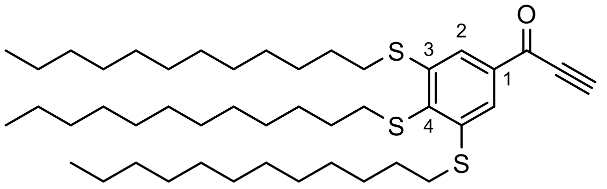

10c
$C_{45}H_{78}OS_3$
(731.30)

^{1}H NMR (700 MHz, $CDCl_3$): δ = 0.85–0.91 (m, 9H, CH_3), 1.20–1.52 (m, 54H, CH_2), 1.57–1.62 (m, 2H, 4-SCH_2CH_2), 1.72–1.78 (m, 4H, 3-SCH_2CH_2), 2.90 (t, J = 7.4 Hz, 2H, 4-SCH_2), 2.94 (t, J = 7.4 Hz, 4H, 3-SCH_2), 3.46 (s, 1H, CCH), 7.64 (s, 2H, 2-H) ppm. ^{13}C NMR (176 MHz, $CDCl_3$) δ = 14.13 (CH_3), 22.70 (CH_2), 28.18 , 29.19, 29.21, 29.27, 29.36, 29.38, 29.53, 29.55, 29.61, 29.64, 29.67, 29.69, 31.92, 31.94 (CH_2), 32.33 (3-SCH_2), 35.02 (4-SCH_2), 80.20 (*C*CH), 81.03 (C*C*H), 120.55 (C-2), 135.35 (C-4), 136.02 (C-1), 147.60 (C-3), 176.60 (CO) ppm. FT-IR (ATR): $\tilde{\nu}$ = 3301 (w), 2921 (s), 2851 (s), 2159 (w), 2095 (w), 1654 (m), 1562 (w), 1534 (m), 1464 (w), 1374 (w), 1239 (s), 1188 (w),

1060 (w), 876 (w), 833 (w), 795 (w), 744 (w), 721 (w), 683 (w), 655 (w) cm^{-1}. MS (ESI): *m/z* = 753.51 [M + Na$^+$]. HRMS (ESI): for $C_{45}H_{78}OS_3Na$ clac.: 753.5107 found: 753.5094.

3,4-Bis(dodecylthio)phenol (11b)

2,3-Dodecylthio-1-bromobenzene **7b** (1.00 g, 1.79 mmol), Cu(acac)$_2$ (47 mg, 179 µmol), BHMPO (59 mg, 179 µmol) and LiOH·H$_2$O (188 mg, 4.48 mmol) was given in a degassed mixture of DMSO (8 mL) and demin. water (2 mL). The suspension was stirred for 6 days at 120 °C. After cooling to room temperature, the mixture was acidified by addition of 2 M HCl (50 mL) and subsequently extracted with EtOAc (3 × 50 mL). The combined organic Phases were dried over MgSO$_4$ and the solvent was removed under reduced pressure. After purification by coulumn chromatrography the phenol **9b** (PE / EtOAc = 20 : 1) was obtained as beige solid (0.56 g, 1.08 mmol, 63 %).

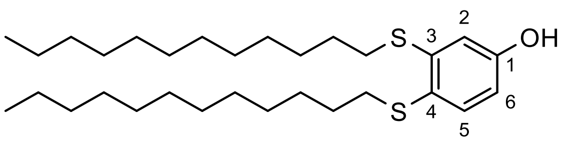

11b
$C_{30}H_{54}OS_2$
(494.88)

^{1}H NMR (500 MHz, CDCl$_3$): δ = 0.88 (t, *J* = 6.8 Hz, 6H, CH$_3$), 1.15–1.75 (m, 36H, CH$_2$), 2.79 (t, *J* = 7.8 Hz, 2H, 4-SCH$_2$), 2.87 (t, *J* = 7.5 Hz, 2H, 3-SCH$_2$), 5.16 (s, 1H, OH), 6.55 (dd, *J* = 8.3 Hz, 2.7 Hz, 1H, 6-H), 6.70 (d, *J* = 2.7 Hz, 1H, 2-H), 7.26 (d, *J* = 8.3 Hz, 1H, 5-H) ppm. ^{13}C NMR (126 MHz, CDCl$_3$) δ = 14.13 (CH$_3$), 22.70, 28.44, 28.81, 29.14, 29.23, 29.25, 29.36, 29.53, 29.54, 29.62, 29.65, 29.67, 31.93 (CH$_2$), 32.47 (3-SCH$_2$), 35.09 (4-SCH$_2$), 112.12 (C-4), 113.16 (C-6), 124.79 (C-2), 134.73 (C-5), 143.34 (C-3), 155.50 (C-1) ppm. FT-IR (ATR): $\tilde{\nu}$ = 3385 (w), 2920 (s), 2851 (s), 1584 (w), 1564 (w), 1460 (m), 1419 (m), 1377 (w), 1268 (w), 1215 (w), 1109 (w), 1036 (w), 901 (m), 840 (w), 805 (w), 721 (w), 642 (w), 593 (w), 447 (w) cm^{-1}. MS (ESI): *m/z* = 493.36 [M – H]$^-$, 529.33 [M + Cl]$^-$, 607. 35, 987.72. HRMS (ESI): for $C_{30}H_{54}OS_2Cl$ calc.: 529.3299 found: 529.3294.

3,4,5-Tris(dodecylthio)phenol (11c)

2,3,4-Dodecylthio-1-bromobenzene **7c** (1.50 g, 1.98 mmol), Cu(acac)$_2$ (52 mg, 198 µmol), BHMPO (65 mg, 198 µmol) and LiOH·H$_2$O (208 mg, 4.95 mmol) was given in a degassed mixture of DMSO (8 mL) and demin. water (2 mL). The suspension was stirred for 6 days at 120 °C. After cooling to room temperature, the mixture was acidified by addition of 2 M HCl (50 mL) and subsequently extracted with EtOAc (3 × 50 mL). The combined organic Phases were dried over MgSO$_4$ and the

solvent was removed under reduced pressure. After purification by coulumn chromatrography the phenol **9b** (PE / EtOAc = 20 : 1) was obtained as yellow solid (0.44 g, 0.63 mmol, 31 %).

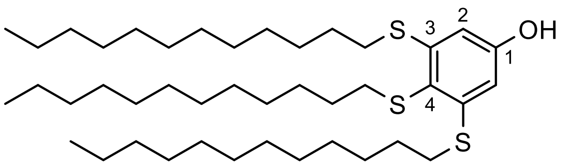

11c
$C_{42}H_{78}OS_3$
(695.27)

^{1}H NMR (500 MHz, $CDCl_3$): δ = 0.82–0.93 (m, 9H, CH_3), 1.20–1.52 (m, 54H, CH_2), 1.52–1.64 (m, 2H, 4-SCH_2CH_2), 1.64–1.77 (m, 4H, 3-SCH_2CH_2), 2.77 (t, J = 7.4 Hz, 2H, 4-SCH_2), 2.81 (t, J = 7.4 Hz, 4H, 3-SCH_2), 4.86 (s, 1H, OH), 6.37 (s, 2H, 2-H) ppm. ^{13}C NMR (126 MHz, $CDCl_3$) δ = 14.13 (CH_3), 22.70, 28.20, 29.03, 29.23, 29.27, 29.30, 29.37, 29.39, 29.51, 29.54, 29.60, 29.63, 29.65, 29.68, 29.72, 31.93, 31.95 (CH_2), 32.30 (2-SCH_2), 35.24 (4-SCH_2), 107.52 (C-2), 119.40 (C-4), 148.11 (C-3), 156.32 (C-1) ppm. FT-IR (ATR): $\tilde{\nu}$ = 3374 (w), 2956 (m), 2915 (s), 2874 (m), 2847 (s), 2170 (w), 1574 (m), 1557 (m), 1466 (m), 1427 (w), 1394 (m), 1268 (w), 1180 (m), 1125 (w), 1041 (w), 951 (w), 889 (w), 826 (m), 803 (m), 721 (w), 604 (w), 541 (w), 498 (w) cm^{-1}. MS (ESI): *m/z* =693.51 $[M–H]^-$, 1388.03 $[2M – H]^-$. HRMS (ESI): for $C_{42}H_{77}OS_3$ calc.: 693.5142 found: 693.5135.

6-(Dodecyloxy)-2-[4-(dodecylthio)phenyl]-7-methoxychromenium triflate (O_1-V-Fla-S_1)

Synthesis according to GP 3: phenol **11d** (98 mg, 318 µmol), ethynylketone **10a** (105 mg, 318 µmol), EtOAc (10 mL), yield: 195 mg, 253 µmol, 80 %, red needles.

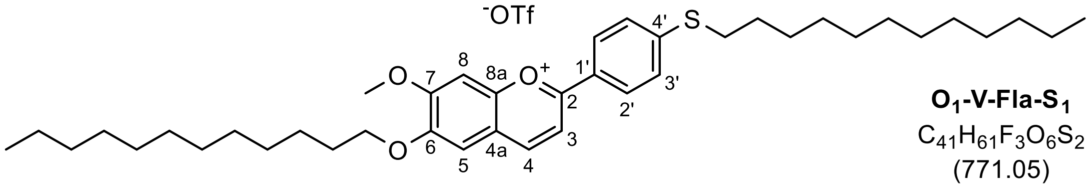

O_1-V-Fla-S_1
$C_{41}H_{61}F_3O_6S_2$
(771.05)

^{1}H NMR (700 MHz, $CDCl_3$): δ = 0.85–0.90 (m, 6H, CH_3), 1.19–1.54 (m, 36H, CH_2), 1.68–1.75 (m, 2H, SCH_2CH_2), 1.86–1.94 (m, 2H, OCH_2CH_2), 2.96 (t, J = 7.5 Hz, 2H, SCH_2), 4.11 (t, J = 6.7 Hz, 2H, OCH_2), 4.24 (s, 3H, OCH_3), 7.22 (d, J = 8.5 Hz, 2H, 3'-H), 7.25 (s, 1H, 5-H), 7.90 (s, 1H, 8-H), 8.14 (d, J = 8.5 Hz, 2H, 2'-H), 8.22 (d, J = 8.7 Hz, 1H, 3-H), 8.95 (d, J = 8.7 Hz, 1H, 4-H) ppm. ^{13}C NMR (176 MHz, $CDCl_3$) δ = 14.13 (CH_3), 22.70, 25.93, 28.35, 28.80, 29.03, 29.23, 29.36, 29.38, 29.43, 29.53, 29.59, 29.61, 29.65, 29.68, 29.71 (CH_2), 31.50 (SCH_2), 31.92, 31.94 (CH_2), 58.36 (OCH_3), 70.23 (OCH_2), 100.91 (C-8), 107.03 (C-5), 113.59 (C-3), 120.45 (C-4a), 120.99 (q,

J = 320.4 Hz, CF_3), 123.62 (C-1'), 126.25 (C-3'), 129.06 (C-2'), 151.32 (C-6), 151.60 (C-4'), 151.72 (C-4), 154.81 (C-8a), 161.44 (C-7), 168.44 (C-2) ppm. FT-IR (ATR): $\tilde{\nu}$ = 3511 (w), 3092 (w), 2955 (s), 2921 (s), 2852 (w), 2183 (w), 2162 (w), 1970 (w), 1624 (w), 1591 (w), 1562 (s), 1543 (m), 1509 (s), 1488 (m), 1466 (m), 1411 (m), 1394 (s), 1344 (s), 1315 (m), 1242 (s), 1209 (s), 1158 (s), 1093 (s), 1048 (s), 1029 (w), 1006 (m), 938 (w), 908 (w), 871 (m), 824 (w), 733 (m), 637 (s), 604 (w), 574 (w), 518 (m), 496 (w), 484 (w), 450 (w) cm^{-1}. MS (ESI): m/z = 621.43 [M^+], 675.44 [M + OMe + Na^+]. HRMS (ESI): for $C_{40}H_{61}O_3S^+$ calc.: 621.4336 found: 621.4328. CHN: calc. for $C_{41}H_{61}F_3O_6S_2$ (771.05): C 63.87, H 7.97, S 8.32; found: C 63.89, H 7.92, S 8.22.

6-(Dodecyloxy)-2-[3,4-bis(dodecylthio)phenyl]-7-methoxychromenium triflate (O_1-V-Fla-S_2)

Synthesis according to GP 3: phenol **11d** (107 mg, 347 µmol), ethynylketone **10b** (181 mg, 341 µmol), EtOAc (10 mL), yield: 128 mg, 132 µmol, 39 %, brown solid.

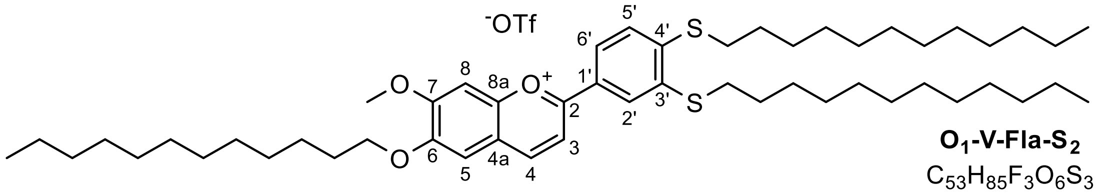

O_1-V-Fla-S_2
$C_{53}H_{85}F_3O_6S_3$
(971.43)

^1H NMR (500 MHz, $CDCl_3$): δ = 0.83–0.92 (m, 9H, CH_3), 1.20–1.57 (m, 54H), 1.62–1.84 (m, 4H, SCH_2CH_2), 1.87–1.97 (m, 2H, OCH_2CH_2), 2.94 (t, J = 7.5 Hz, 2H, 4´-SCH_2), 3.11 (t, J = 7.2 Hz, 2H, 3´-SCH_2), 4.15 (t, J = 6.7 Hz, 2H, OCH_2), 4.28 (s, 3H, OCH_3), 7.11 (s, 1H, 5-H), 7.13 (d, J = 8.7 Hz, 1H, 5´-H), 7.78 (d, J = 2.1 Hz, 1H, 2´-H), 7.95 (s, 1H, 8-H), 8.18 (dd, J = 8.7 Hz, 2.1 Hz, 1H, 6´-H), 8.41 (d, J = 8.7 Hz, 1H, 3-H), 8.83 (d, J = 8.7 Hz, 1H 4-H) ppm. ^{13}C NMR (126 MHz, $CDCl_3$) δ = 14.13 (CH_3), 22.69, 22.71, 25.98, 28.08, 28.48, 28.81, 28.82, 29.20, 29.30, 29.37, 29.39, 29.45, 29.53, 29.55, 29.62, 29.65, 29.67, 29.69, 29.70, 29.73, 31.92 (CH_2), 31.94, 32.36 (4'-SCH_2), 33.39 (3'-SCH_2), 58.42 (OCH_3), 70.14 (OCH_2), 101.12 (C-8), 106.60 (C-5), 114.00 (C-3), 120.48 (C-4a), 121.00 (q, J = 321.1 Hz, CF_3), 123.71 (C-1´), 124.79 (C-5'), 125.66 (C-2'), 126.56 (C-6'), 137.18 (C-3'), 150.61 (C-4'), 151.34 , 151.40 (C-6, C-4), 154.88 (C-8a), 161.52 (C-7), 167.85 (C-2) ppm. FT-IR (ATR): $\tilde{\nu}$ = 3095 (w), 2921 (s), 2852 (s), 2071 (w), 1968 (w), 1956 (w), 1625 (m), 1580 (w), 1557 (m), 1519 (s), 1466 (m), 1407 (m), 1388 (m), 1343 (m), 1260 (s), 1244 (s), 1158 (m), 1111 (m), 1065 (w), 1030 (m), 999 (w), 949 (w), 870 (w), 815 (w), 755 (w), 741 (w), 722 (w), 638 (m), 607 (w), 573 (w), 517 (w), 466 (w), 425 (w) cm^{-1}. MS (ESI): m/z = 821.59 [M^+].

HRMS (ESI): for $C_{52}H_{85}O_3S_2^+$ calc.: 821.5935 found: 821.5919. CHN: calc. for $C_{53}H_{85}F_3O_6S_3$ (971.43): C 65.53, H 8.82, S 9.90; found.: C 65.54, H 8.99, S 9.42.

7-(Dodecyloxy)-2-[4-(dodecylthio)phenyl]-6-methoxychromenium triflate (O_1-iV-Fla-S_1)

Synthesis according to GP 3: phenol **11e** (97 mg, 315 µmol), ethynylketone **10a** (104 mg, 315 µmol), EtOAc (10 mL), yield: 179 mg, 232 µmol, 74 %, orange solid.

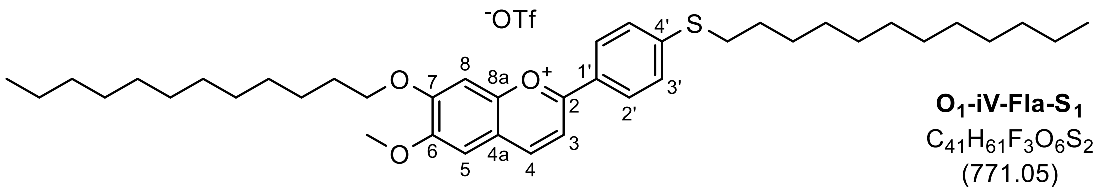

O_1-iV-Fla-S_1
$C_{41}H_{61}F_3O_6S_2$
(771.05)

^{1}H NMR (700 MHz, $CDCl_3$): δ = 0.83–0.91 (m, 6H, CH_3), 1.19–1.57 (m, 36H, CH_2), 1.69–1.75 (m, 2H, SCH_2CH_2), 1.91–2.01 (m, 2H, OCH_2CH_2), 2.96 (t, *J* = 7.4 Hz, 2H, SCH_2), 4.03 (s, 3H, OCH_3), 4.38 (t, *J* = 6.7 Hz, 2H, OCH_2), 7.22 (d, *J* = 8.7 Hz, 2H, 3'-H), 7.33 (s, 1H, 5-H), 7.83 (s, 1H, 8-H), 8.14 (d, *J* = 8.7 Hz, 2H, 2'-H), 8.22 (d, *J* = 8.6 Hz, 1H, 3-H), 8.99 (d, *J* = 8.6 Hz, 1H, 4-H) ppm. ^{13}C NMR (176 MHz, $CDCl_3$) δ = 14.13 (CH_3), 22.70, 25.79, 28.33, 28.66, 29.02, 29.23, 29.36, 29.38, 29.39, 29.53, 29.55, 29.60, 29.63, 29.65, 29.67, 29.70 (CH_2), 31.48 (SCH_2), 31.93 (CH_2), 56.96 (OCH_3), 71.85 (OCH_2), 101.18 (C-8), 106.72 (C-5), 113.44 (C-3), 120.33 (C-4a), 120.98 (q, *J* = 320.4 Hz, CF_3), 123.65 (C-1'), 126.26 (C-3'), 129.02 (C-2'), 151.50 (C-4'), 151.81, 151.87 (C-4, C-6), 155.03 (C-8a), 160.88 (C-7), 168.41 (C-2) ppm. FT-IR (ATR): $\tilde{\nu}$ = 3503 (w), 3060 (w), 2922 (s), 2852 (m), 2130 (w), 2052 (w), 2028 (w), 1994 (w), 1626 (w), 1592 (w), 1564 (w), 1545 (w), 1526 (m), 1512 (m), 1487 (w), 1466 (w), 1441 (w), 1407 (w), 1389 (w), 1348 (m), 1314 (w), 1261 (s), 1245 (s), 1208 (m), 1180 (w), 1158 (m), 1095 (m), 1030 (m), 1012 (w), 955 (w), 872 (w), 823 (w), 742 (w), 638 (m), 605 (w), 574 (w), 518 (w), 497 (w), 452 (w), 412 (w) cm^{-1}. MS (ESI): *m/z* = 621.43 [M^+], 675.44 [M + OMe + Na^+]. HRMS (ESI): for $C_{40}H_{61}O_3S^+$ calc: 621.4336 found: 621.4298. CHN: calc. for $C_{41}H_{61}F_3O_6S_2$ (771.04): C 63.87, H 7.97, S 8.32; found: C 63.12, H 7.99, S 8.07.

7-(Dodecyloxy)-2-[3,4-bis(dodecylthio)phenyl]-6-methoxychromenium triflate (O_1-iV-Fla-S_2)

Synthesis according to GP 3: phenol **11e** (104 mg, 334 µmol), ethynylketone **10b** (177 mg, 334 µmol), EtOAc (10 mL), yield: 261 mg, 269 µmol, 81 %, brown waxy solid.

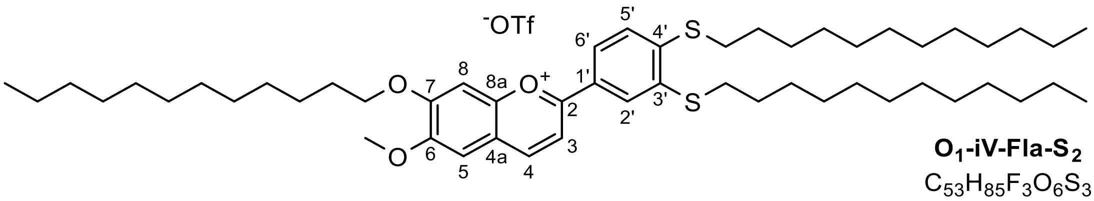

^{1}H NMR (500 MHz, $CDCl_3$): δ = 0.81–0.92 (m, 9H, CH_3), 1.17–1.59 (m, 54H, CH_2), 1.65–1.73 (m, 2H, 3'-SCH_2CH_2), 1.73–1.81 (m, 2H, 4'-SCH_2CH_2), 1.92–2.02 (m, 2H, OCH_2CH_2), 2.96 (t, J = 7.4 Hz, 2H, 4'-SCH_2), 3.10 (t, J = 7.21 Hz, 2H, 3'-SCH_2), 4.06 (s, 3H, OCH_3), 4.43 (t, J = 6.62 Hz, 2H, OCH_2), 7.15 (d, J = 8.5 Hz, 1H, 5'-H), 7.23 (s, 1H, 5-H), 7.81–7.84 (m, 1H, 2'-H), 7.86 (s, 1H, 8-H), 8.16 (dd, J = 8.5 Hz, 2.1 Hz, 1H, 6´-H), 8.41 (d, J = 8.7 Hz, 1H, 3-H), 8.88–8.97 (m, 1H, 4-H) ppm. ^{13}C NMR (126 MHz, $CDCl_3$) δ = 14.13 (CH_3), 22.70, 25.84, 28.05, 28.58, 28.73, 28.82, 29.17, 29.29, 29.36, 29.38, 29.41, 29.54, 29.57, 29.63, 29.66, 29.68, 29.70, 29.71, 31.94, 32.33 (4'-SCH_2), 33.52 (3'-SCH_2), 56.91 (OCH_3), 71.87 (OCH_2), 101.27 (C-8), 106.37 (C-5), 113.92 (C-3), 120.42 (C-4a), 120.89 (q, J = 320.3 Hz, CF_3), 123.82 (C-1'), 124.85 (C-5'), 126.17 (C-2´), 126.64 (C-6'), 136.95 (C-3'), 150.89 (C-4'), 151.62 (C-4), 151.96 (C-6), 155.16 (C-8a), 161.02 (C-7), 167.91 (C-2) ppm. FT-IR (ATR): $\tilde{\nu}$ = 2921 (s), 2852 (s), 2154 (w), 1626 (m), 1579 (w), 1557 (m), 1520 (s), 1458 (m), 1497 (m), 1386 (m), 1342 (m), 1260 (s), 1243 (s), 1208 (m), 1158 (m), 1110 (m), 1030 (m), 1013 (w), 975 (w), 951 (w), 867 (w), 818 (w), 756 (w), 740 (w), 723 (w), 638 (m), 608 (w), 573 (w), 517 (w), 476 (w), 450 (w) cm^{-1}. MS (ESI): m/z = 821.59 [M^+], 875.60 [M + OMe + Na^+], 1021.80 [(M + 2 OMe +Na^+]. HRMS (ESI): for $C_{52}H_{85}O_3S_2^+$ calc.: 821.5935 found: 821.5909. CHN: calc. for $C_{53}H_{85}F_3O_6S_3$ (971.43): C 65.53, H 7.97, S 9.90; found.: C 69.29, H 8.93, S 9.91.

7-(Dodecyloxy)-2-[3,4,5-tris(dodecylthio)phenyl]-6-methoxychromenium triflate (O$_1$-iV-Fla-S$_3$)

Synthesis according to GP 3: phenol **11e** (50 mg, 161 µmol), ethynylketone **10c** (118 mg, 161 µmol), EtOAc (10 mL), yield: 106 mg, 90 µmol, 56 %, red-brown solid.

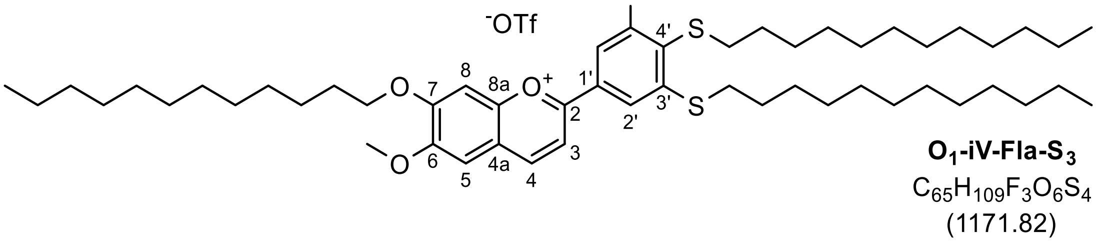

^{1}H NMR (500 MHz, $CDCl_3$): δ = 0.83–0.91 (m, 12H, CH_3), 1.19–1.46 (m, 66H, CH_2), 1.50–1.65 (m, 8H, CH_2), 1.67–1.81 (m, 4H, 3'-SCH_2CH_2), 1.95–2.04 (m, 2H, OCH_2CH_2), 2.93 (t, *J* = 7.4 Hz, 2H, 4'-SCH_2), 3.08 (t, *J* = 7.1 Hz, 4H, 3'-SCH_2), 4.06 (s, 3H, OCH_3), 4.46 (t, *J* = 6.5 Hz, 2H, OCH_2), 7.41 (d, *J* = 7.35 Hz, 1H, 5-H), 7.54 (s, 2H, 2'-H), 7.73 (d, *J* = 5.6 Hz, 1H, 8-H), 8.49 (d, *J* = 8.7 Hz, 1H, 3-H), 9.26 (d, *J* = 8.7 Hz, 1H, 4-H) ppm. ^{13}C NMR (126 MHz, $CDCl_3$) δ = 14.12 (CH_3), 22.70, 25.87, 27.99, 28.66, 28.96, 29.04, 29.27, 29.39, 29.48, 29.55, 29.60, 29.62, 29.64, 29.68, 29.70, 29.73, 29.80, 31.94 (CH_2), 32.56 (3'-SCH_2), 35.22 (4'-SCH_2), 57.21 (OCH_3), 72.04 (OCH_2), 100.63 (C-8), 106.77 (C-5), 114.83 (C-3), 116.99 (C-2'), 120.81 (q, *J* = 320.4 Hz, CF_3), 121.72 (C-4a), 128.91 (C-1'), 136.81 (C-4'), 149.53 (C-3'), 152.55 (C-6), 153.21 (C-4), 156.25 (C-8a), 162.31 (C-7), 167.62 (C-2) ppm. FT-IR (ATR): $\tilde{\nu}$ = 2955 (m), 2921 (s), 2852 (s), 2199 (w), 2151 (w), 2025 (w), 1624 (w), 1550 (w), 1512 (m), 1466 (m), 1412 (m), 1377 (m), 1325 (s), 1283 (s), 1257 (s), 1244 (s), 1208 (m), 1182 (w), 1160 (m), 1124 (m), 1031 (m), 1008 (w), 955 (w), 866 (w), 804 (w), 722 (w), 638 (m), 573 (w), 518 (w), 486 (w) cm^{-1}. MS (ESI): *m/z* = 1021.75 [M^+], 1075.80 [M + OMe + Na^+]. HRMS (ESI): Für $C_{64}H_{109}O_2S_3^+$ calc.: 1021.7533 found: 1021.7520. CHN: calc. for $C_{65}H_{109}F_3O_6S_4$ (971.43): C 66.62, H 9.38, S 10.94; found.: C 67.10, H 9.52, S 10.58.

6,7-Bis(dodecyloxy)-2-[4-(dodecylthio)phenyl]chromenium triflate (O_2-Fla-S_1)

Synthesis according to GP 3: phenol **11f** (156 mg, 338 µmol), ethynylketone **10a** (101 mg, 306 µmol), EtOAc (10 mL), yield: 133 mg, 144 µmol, 47 %, red solid.

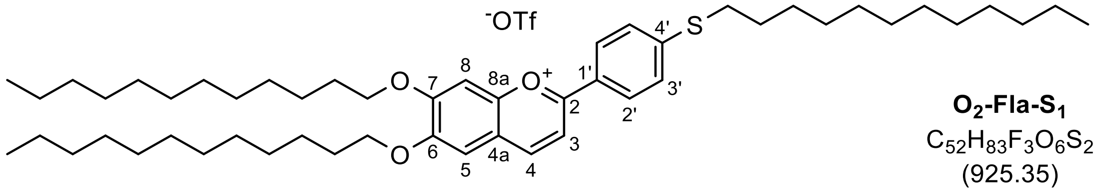

O_2-Fla-S_1
$C_{52}H_{83}F_3O_6S_2$
(925.35)

^{1}H NMR (700 MHz, $CDCl_3$): *δ* = 0.84–0.91 (m, 9H, CH_3), 1.19–1.58 (m, 54H, CH_2), 1.63–1.75 (m, 2H, SCH_2CH_2), 1.86–1.98 (m, 4H, OCH_2CH_2), 2.96 (t, *J* = 7.5 Hz, 2H, SCH_2), 4.12 (t, *J* = 6.5 Hz, 2H, 7-OCH_2), 4.38 (t, *J* = 6.5 Hz, 2H, 6-OCH_2), 7.21–7.23 (m, 2H, 3'-H), 7.23 (s, 1H, 5-H), 7.80 (s, 1H, 8-H), 8.13 (d, *J* = 8.5 Hz, 2H, 2´-H), 8.23 (d, *J* = 8.7 Hz, 1H, 3-H), 8.96 (d, *J* = 8.7 Hz, 1H, 4-H) ppm. ^{13}C NMR (176 MHz, $CDCl_3$) δ = 14.13 (CH_3), 22.71, 25.86, 26.00, 28.36, 28.65, 28.81, 29.05, 29.25, 29.37, 29.38, 29.40, 29.44, 29.54, 29.62, 29.63, 29.66, 29.68, 29.71, 29.75 (CH_2), 31.50 (SCH_2), 31.93, 31.95 (CH_2), 70.02 (6-OCH_2), 71.61 (7-OCH_2), 101.03 (C-8), 107.00 (C-5), 113.44 (C-3), 120.42 (C-4a), 120.99 (q, *J* = 320.4 Hz, CF_3), 123.75 (C-1'), 126.29 (C-3'), 128.93 (C-2'), 151.24 (C-4'), 151.52 (C-4), 151.63 (C-6), 154.99 (C-8a), 161.21 (C-7), 168.18 (C-2) ppm. FT-

IR (ATR): $\tilde{\nu}$ = 3499 (w), 3060 (w), 2956 (w), 2919 (s), 2851 (s), 2163 (w), 2002 (w), 1624 (m), 1592 (m), 1563 (m), 1523 (s), 1511 (s), 1487 (m), 1467 (m), 1431 (w), 1406 (m), 1390 (m), 1346 (s), 1315 (m), 1243 (s), 1196 (m), 1156 (s), 1095 (s), 1030 (s), 1007 (w), 952 (w), 911 (w), 871 (w), 823 (w), 756 (w), 742 (w), 721 (w), 637 (m), 605 (w), 574 (w), 554 (w), 518 (m), 496 (w), 451 (w) cm^{-1}. MS (ESI): *m/z* = 775.60 [M^+], 829.61 [M + OMe + Na^+], 1637.23 [(M + OMe + Na^+]. HRMS (ESI): for $C_{52}H_{83}O_3S^+$ calc: 775.6057 found: 775.6007. CHN: calc. for $C_{52}H_{83}F_3O_6S_2$ (925.35): C 67.50, H 9.04, S 6.93; found: C 67.62, H 9.09, S 6.46.

6,7-Bis(dodecyloxy)-2-[3,4-bis(dodecylthio)phenyl]chromenium triflate (O_2-Fla-S_2)

Synthesis according to GP 3: phenol **11f** (130 mg, 218 µmol), ethynylketone **10b** (149 mg, 281 µmol), EtOAc (10 mL), yield: 270 mg, 240 µmol, 85 %, brown solid.

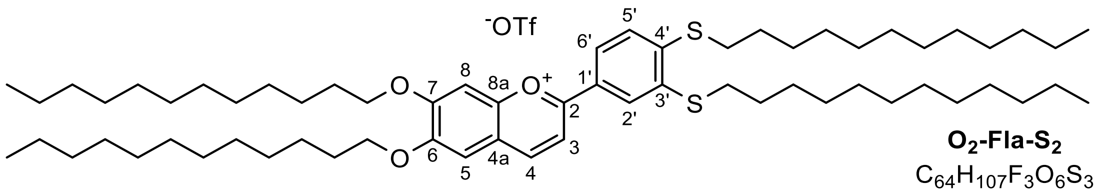

O_2-Fla-S_2
$C_{64}H_{107}F_3O_6S_3$
(1125.73)

^{1}H NMR (300 MHz, $CDCl_3$): δ = 0.77–0.95 (m, 12H, CH_3), 1.10–2.04 (m, 72H, CH_2), 2.95 (t, *J* = 7.4 Hz, 2H, 4'-SCH_2), 3.09 (t, *J* = 7.2 Hz, 2H, 3´-SCH_2), 4.15 (t, *J* = 6.4 Hz, 2H, 6-OCH_2), 4.42 (t, *J* = 6.4 Hz, 2H, 7-OCH_2), 7.12-7.18 (m, 2H, 5´-H, 5-H), 7.79 (d, *J* = 2.1 Hz, 1H, 2´-H), 7.83 (s, 1H, 8-H), 8.15 (dd, *J* = 8.9 Hz, 2.1 Hz, 1H, 6´-H), 8.39 (d, *J* = 8.7 Hz, 1H, 3-H), 8.87 (d, *J* = 8.7 Hz, 1H, 4-H) ppm. ^{13}C NMR (176 MHz, $CDCl_3$) δ = 14.13 (CH_3), 22.70, 22.71, 25.91, 26.03, 28.08, 28.55, 28.74, 28.84, 29.22, 29.35, 29.38, 29.39, 29.42, 29.44, 29.47, 29.55, 29.57, 29.66, 29.68, 29.72, 29.77, 31.93, 31.94, 31.96, 31.96 (CH_2), 32.32 (4'-SCH_2), 33.45 (3'-SCH_2), 69.91 (6-OCH_2), 71.64 (7-OCH_2), 101.13 (C-8), 106.67 (C-5), 113.74 (C-3), 120.49 (C-4a), 120.88 (q, *J* = 320.3 Hz, CF_3), 123.84 (C-1'), 124.83 (C-5'), 125.85 (C-2'), 126.47 (C-6'), 136.94 (C-3'), 150.48 (C-4'), 151.27 (C-4), 151.56 (C-6), 155.05 (C-8a), 161.29 (C-7), 167.57 (C-2) ppm. FT-IR (ATR): $\tilde{\nu}$ = 3085 (w), 2954 (w) 2918 (s), 2851 (s), 2180 (w), 2163 (w), 2044 (w), 1977 (w), 1625 (w), 1581 (w), 1558 (w), 1519 (s), 1466 (m), 1408 (m), 1384 (m), 1342 (m), 1261 (s), 1245 (s), 1196 (w), 1155 (m), 1111 (m), 1068 (w), 1031 (m), 986 (w), 961 (w), 909 (w), 872 (w), 722 (w), 638 (m), 573 (w), 557 (w), 517 (w), 452 (w) cm^{-1}. MS (ESI): *m/z* = 975.76 [M^+]. HRMS (ESI): for $C_{63}H_{107}O_3S_2^+$ calc.: 975.7656 found: 975.7637. CHN: calc. for $C_{64}H_{107}F_3O_6S_3$ (1125.73): C 68.28, H 8.53, S 8.54; found.: C 68.20, H 9.72, S 8.41.

6,7-Bis(dodecyloxy)-2-[3,4,5-bis(dodecylthio)phenyl]chromenium triflate (O_2-Fla-S_3)

Synthesis according to GP 3: phenol **11f** (85 mg, 185 µmol), ethynylketone **10c** (122 mg, 168 µmol), EtOAc (10 mL), yield: 184 mg, 139 µmol, 83 %, brown solid.

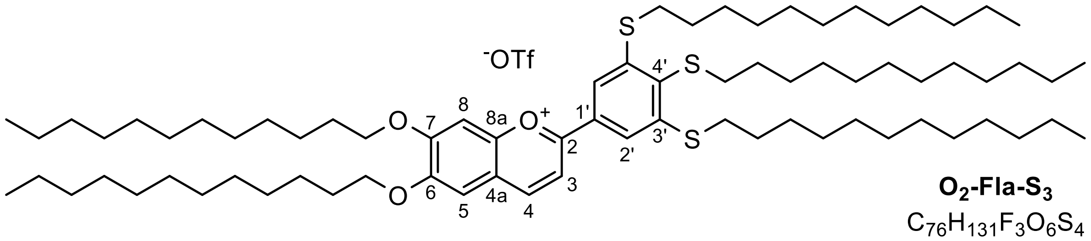

O_2-Fla-S_3
$C_{76}H_{131}F_3O_6S_4$
(1326.11)

^{1}H NMR (500 MHz, $CDCl_3$): δ = 0.84–0.91 (m, 15H, CH_3), 1.19–1.69 (m, 92H, CH_2), 1.73–1.81 (m, 4H, 3'-$SCH_2C\mathit{H}_2$), 1.86–1.93 (m, 2H, 6-$OCH_2C\mathit{H}_2$), 1.94–2.03 (m, 2H, 7-$OCH_2C\mathit{H}_2$), 2.92 (t, J = 7.4 Hz, 2H, 4'-SCH_2), 3.07 (t, J = 7.1 Hz, 4H, 3'-SCH_2), 4.15 (t, J = 6.5 Hz, 2H, 6-OCH_2), 4.45 (t, J = 6.3 Hz, 2H, 7-OCH_2), 7.34 (s, 1H, 5-H), 7.53 (s, 2H, 2'-H), 7.69 (s, 1H, 8-H), 8.47 (d, J = 8.6 Hz, 1H, 3-H), 9.24 (d, J = 8.6 Hz, 1H, 4-H) ppm. ^{13}C NMR (126 MHz, $CDCl_3$) δ = 14.12 (CH_3), 22.70, 22.72, 25.93, 25.97, 28.00, 28.65, 28.73, 28.97, 29.05, 29.29, 29.39, 29.41, 29.50, 29.61, 29.64, 29.69, 29.71, 29.74, 29.76, 29.77, 29.81, 31.94, 31.96 (CH_2), 32.56 (4'-SCH_2), 35.19 (3'-SCH_2), 70.25 (6-OCH_2), 71.79 (7-OCH_2), 100.49 (C-8), 107.02 (C-5), 114.71 (C-3), 118.64 (C-2'), 120.83 (d, J = 320.5 Hz, CF_3), 121.81 (C-4a), 128.93 (C-1'), 136.69 (C-4'), 149.53 (C-3'), 152.14 (C-6), 152.98 (C-4), 156.11 (C-8a), 162.53 (C-7), 167.30 (C-2) ppm. FT-IR (ATR): $\tilde{\nu}$ = 2921 (s), 2852 (s), 2175 (w), 1625 (w), 1507 (s), 1466 (m), 1413 (m), 1375 (w), 1325 (s), 1245 (s), 1225 (m), 1200 (w), 1166 (m), 1227 (w), 1029 (m), 858 (w), 803 (w), 757 (w), 639 (m), 595 (w), 573 (w), 517 (w) cm^{-1}. MS (ESI): m/z = 1175.92 [M$^+$]. HRMS (ESI): for $C_{75}H_{131}O_3S_3^+$ calc: 1175.9255 found: 1175.9240. CHN: calc. for $C_{76}H_{131}F_3O_6S_4$ (1326.11): C 68.84, H 9.96, S 9.67; found: C 63.81, H 10.07, S 9.00.

5,6,7-Tris(dodecyloxy)-2-[4- (dodecylthio)phenyl]chromenium triflat (O_3-Fla-S_1)

Synthesis according to GP 3: phenole **11g** (200 mg, 308 µmol), ethynylketone **10a** (101 mg, 306 µmol), EtOAc (5 mL), yield: 63 mg, 57 µmol, 19 %, red solid.

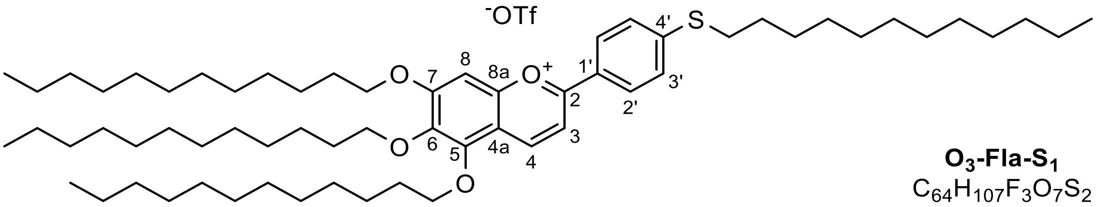

O_3-Fla-S_1
$C_{64}H_{107}F_3O_7S_2$
(1109.67)

^{1}H NMR (700 MHz, $CDCl_3$): δ = 0.81–0.96 (m, 12H, CH_3), 1.20–1.60 (m, 72H, CH_2), 1.66–1.73 (m, 2H, $SCH_2C\mathit{H}_2$), 1.78–1.90 (m, 4H, 5,6-$OCH_2C\mathit{H}_2$), 1.93–2.01 (m, 2H, 7- $OCH_2C\mathit{H}_2$), 2.94 (t, *J* = 7.5 Hz, 2H, SCH_2), 4.07 (t, *J* = 6.6 Hz, 2H, 6-OCH_2), 4.38 (t, *J* = 6.8 Hz, 2H, 5-OCH_2), 4.42 (t, *J* = 6.4 Hz, 2H, 7-OCH_2), 7.23 (d, *J* = 8.4 Hz, 2H, 3'-H), 7.76 (s, 1H, 8-H), 8.23–8.35 (m, 3H, 2'-H, 3-H), 8.98 (d, *J* = 8.73 Hz, 1H, 4-H) ppm. ^{13}C NMR (176 MHz, $CDCl_3$) δ = 14.13 (CH_3), 22.71, 25.94, 26.03, 26.05, 28.31, 28.89, 28.99, 29.03, 29.26, 29.37, 29.39, 29.45, 29.54, 29.55, 29.62, 29.66, 29.69, 29.72, 29.73, 29.76, 30.23 (CH_2), 31.52 (SCH_2), 31.94, 31.95 (CH_2), 71.85 (7-OCH_2), 74.70 (6-OCH_2), 75.45 (5-OCH_2), 97.38 (C-8), 112.57 (C-3), 116.42 (C-6), 121.02 (q, *J* = 320.14 Hz, CF_3), 123.71 (C-4a), 126.30 (C-3'), 129.85 (C-2'), 140.97 (C-4a), 148.27, 148.31 (C-4, C-5), 152.22 (C-4'), 154.64 (C-8a), 165.45 (C-7), 170.27 (C-2) ppm. FT-IR (ATR): $\tilde{\nu}$ = 2921 (s), 2852 (s), 1626 (m), 1590 (m), 1555 (m), 1521 (s), 1493 (s), 1467 (m), 1430 (m), 1380 (s), 1339 (s), 1257 (s), 1238 (s), 1156 (m), 1104 (m), 1088 (m), 1030 (s), 108 (w), 827 (w), 721 (w), 639 (m), 572 (w), 517 (w) cm^{-1}. MS (ESI): *m/z* = 959.78 [M^+], 999.77 [M + $OMeNa^+$]. HRMS (ESI): for $C_{63}H_{107}O_4S^+$ calc: 959.7885 found: 959.7817. CHN: calc. for $C_{64}H_{107}F_3O_7S_2$ (1109.67): C 69.27, H 9.72, S 5.78; found.: C 69.48, H 9.81, S 5.18.

5,6,7-Tris(dodecyloxy)-2-[3,4,5-tris(dodecylthio)phenyl]chromenium triflate (O_3-Fla-S_3)

Synthesis according to GP 3: phenol **11g** (133 mg, 207 µmol), ethynylketon **10c** (151 mg, 207 µmol), EtOAc (10 mL), yield: 46 mg, 26 µmol, 13 %, brown solid.

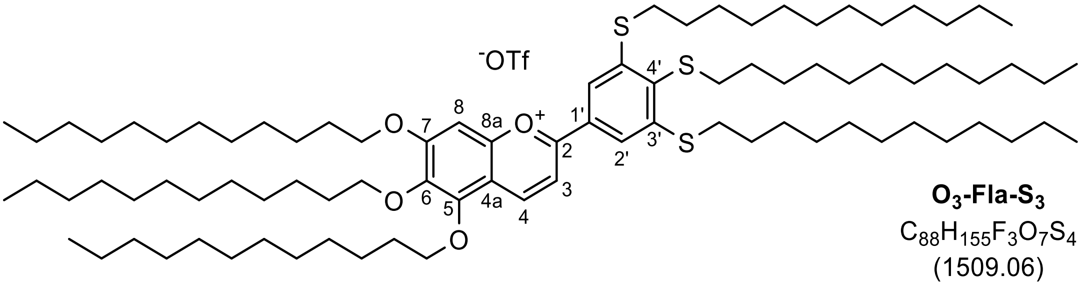

O_3-Fla-S_3
$C_{88}H_{155}F_3O_7S_4$
(1509.06)

^{1}H NMR (500 MHz, $CDCl_3$): δ = 0.85–0.92 (m, 18H), 1.18–2.03 (m, 120H, CH_2), 2.89 (t, J = 7.4 Hz, 2H, 4'-SCH_2), 3.08 (t, J = 7.1 Hz, 4H, 3'-SCH_2), 4.11 (t, J = 6.5 Hz, 2H, OCH_2), 4.37–4.50 (m, 4H, OCH_2), 7.48 (s, 1H, 8-H), 7.60 (s, 2H, 2'-H), 8.56 (d, J = 8.7 Hz, 1H, 3-H), 9.28 (d, J = 8.7 Hz, 2.33 Hz, 1H, 4-H) ppm. ^{13}C NMR (176 MHz, $CDCl_3$) δ = 14.13 (CH_3), 22.71, 25.87, 26.06, 26.14, 27.99, 28.88, 28.95, 29.06, 29.27, 29.40, 29.42, 29.45, 29.47, 29.52, 29.58, 29.63, 29.66, 29.70, 29.71, 29.73, 29.75, 29.78, 30.18, 30.24, 31.94, 31.96 (CH_2), 32.61 (3'-SCH_2), 35.24 (4'-SCH_2), 71.95, 74.88, 75.61 (OCH_2), 96.64 (C-8), 113.92 (C-3), 118.04 (C-4a), 119.27 (C-2'), 120.88 (q, J = 320.6 Hz, CF_3), 129.06 (C-1'), 137.15 (C-4'), 141.23 (C-6, C-7), 148.59 (C-5), 149.31 (C-3'), 150.30 (C-4), 155.60 (C-8a), 166.85 (C-6, C-7), 169.35 (C-2) ppm. FT-IR (ATR): $\tilde{\nu}$ = 2920 (s), 2852 (s), 1628 (w), 1548 (m), 1518 (m), 1493 (m), 1466 (m), 1427 (m), 1366 (m), 1320 (s), 1253 (s), 1224 (m), 1161 (m), 1134 (m), 1111 (w), 1076 (w), 1029 (s), 855 (w), 803 (w), 721 (w), 639 (m), 573 (w), 518 (w) cm^{-1}. MS (ESI): m/z = 1360.18 [M^+], 1414.23 [M + OMe + Na^+], 1423.24. HRMS (ESI): for $C_{87}H_{155}O_4S_3^+$ calc: 1360.1082 found: 1360.1882. CHN: calc. for $C_{88}H_{155}F_3O_7S_4$ (1510.44): C 69.98, H 10.34, S 8.49; found: C 70.11, H 10.29, S 7.62.

6,7-Bis(dodecylthio)-2-(4-(dodecylthio)phenyl)chromenium triflate (S_2-Fla-S_1)

Synthesis according to GP 3: phenol **11b** (200 mg, 404 µmol), ethynylketone **10a** (134 mg, 404 µmol), EtOAc (10 mL), yield: 125 mg, 131 µmol, 32 %, brown solid.

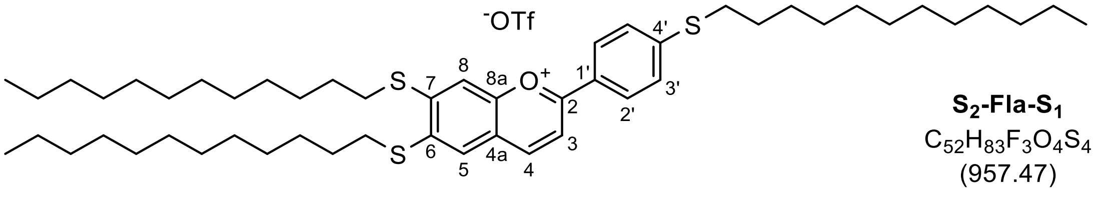

S_2-Fla-S_1
$C_{52}H_{83}F_3O_4S_4$
(957.47)

^{1}H NMR (700 MHz, $CDCl_3$): δ = 0.85–0.91 (m, 9H, CH_3), 1.17–1.61 (m, 54H, CH_2), 1.68–1.75 (m, 2H, 4'-SCH_2CH_2), 1.75–1.82 (m, 2H, 6-SCH_2CH_2), 1.82–1.88 (m, 2H, 7-SCH_2CH_2), 2.95 (t, J = 7.5 Hz, 2H, 4'-SCH_2), 3.10 (t, J = 7.4 Hz, 2H, 6-SCH_2), 3.33 (t, J = 7.2 Hz, 2H, 7-SCH_2), 7.21 (dd, J = 8.9 Hz, 2.03 Hz, 2H, 3'-H), 7.52 (s, 1H, 5-H), 8.10 (s, 1H, 8-H), 8.20 (d, J = 8.9 Hz, 2H, 2'-H), 8.22 (d, J = 8.7 Hz, 1H, 3-H), 8.86 (d, J = 8.7 Hz, 1H 4-H) ppm. ^{13}C NMR (176 MHz, $CDCl_3$) δ = 14.13 (CH_3), 22.71, 27.45, 27.95, 28.25, 28.91, 29.07, 29.09, 29.26, 29.30, 29.34, 29.38, 29.49, 29.54, 29.62, 29.65, 29.66, 29.69, 29.71 (CH_2), 31.56 (4'-SCH_2), 31.94 (CH_2), 33.36 (6-SCH_2), 33.53 (7-SCH_2), 112.67 (C-8), 114.63 (C-3), 120.95 (q, J = 320.4 Hz, CF_3), 121.31 (C-4a), 122.56 (C-5), 123.20 (C-1'), 126.19 (C-3'), 129.96 (C-2'), 140.99 (C-6), 151.52 (C-4), 153.55 (C-4'), 153.81 (C-8a), 156.69 (C-7), 169.33 (C-2) ppm. FT-IR (ATR): $\tilde{\nu}$ = 3500 (w), 3096 (w), 2921 (s), 2852 (s),

1607 (m), 1590 (m), 1561 (m), 1508 (s), 1481 (s), 1345 (s), 1258 (s), 1226 (m), 1191 (m), 1158 (m), 1093 (s), 1057 (s), 1030 (w), 1006 (w), 970 (w), 929 (w), 878 (w), 823 (w), 743 (w), 722 (w), 638 (m), 601 (w), 574 (w), 517 (w), 494 (w), 443 (w) cm^{-1}. MS (ESI): *m/z* = 807.55 [M$^+$], 825.56 [M + OH], 861.56 [M + OMe + Na$^+$]. HRMS (ESI): for $C_{51}H_{83}S_3^+$ calc.: 807.5601 found: 807.5573. CHN: calc. for $C_{52}H_{83}F_3O_4S_4$ (957.47): C 65.23, H 8.74, S 13.39; found: C 65.35, H 8.75, S 13.16.

5,6,7-Tris(dodecylthio)-2-(4-(dodecylthio)phenyl)chromenium triflate (S_3-Fla-S_1)

Synthesis according to GP 3: phenol **11c** (144 mg, 207 µmol), ethynylketone **10a** (68 mg, 207 µmol), EtOAc (10 mL), yield: 46 mg, 40 µmol, 19 %, brown solid.

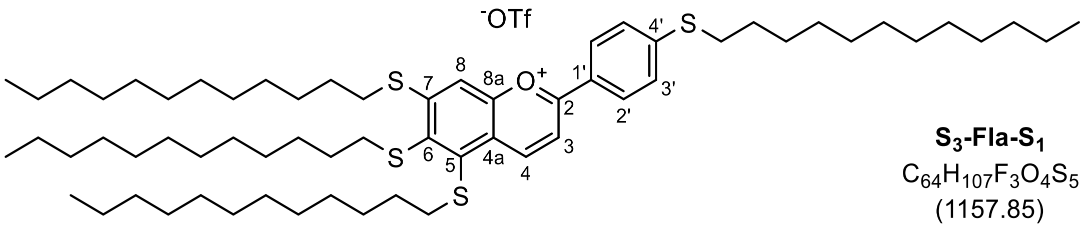

S_3-Fla-S_1
$C_{64}H_{107}F_3O_4S_5$
(1157.85)

^{1}H NMR (500 MHz, $CDCl_3$): δ = 0.82–0.93 (m, 12H, CH_3), 1.17–1.77 (m, 72H, CH_2), 1.85–1.96 (m, 2H, 7-SCH_2CH_2), 2.90 (t, J = 7.5 Hz, 2H, 4'-SCH_2), 2.97–3.06 (m, 4H, 5-SCH_2, 6-SCH_2), 3.28 (t, J = 7.0 Hz, 2H, 7-SCH_2), 7.14 (d, J = 8.5 Hz, 2H, 3'-H), 8.11 (s, 1H, 8-H), 8.37 (d, J = 8.5 Hz, 2H, 2'-H), 8.46 (d, J = 9.1 Hz, 1H, 3-H), 9.36 (d, J = 9.1 Hz, 1H, 4-H) ppm. ^{13}C NMR (126 MHz, $CDCl_3$) *a* = 14.13 (CH_3), 22.71, 27.27, 28.19, 28.86, 28.97, 29.05, 29.19, 29.24, 29.28, 29.36, 29.39, 29.40, 29.52, 29.62, 29.67, 29.72, 29.91, 31.63, 31.92 (CH_2), 31.94 (4'-SCH_2), 34.14 (7-SCH_2), 37.29 , 39.07 (5-SCH_2, 6-SCH_2), 113.22 (C-8), 115.39 (C-3), 123.12 (C-1'), 123.56 (q, J = 320.8 Hz, CF_3), 123.95 (C-4a), 126.17 (C-3'), 130.95 (C-2'), 142.10 (C-6), 144.05 (C-5), 152.68 (C-4), 154.34 (C-4'), 156.18 (C-8a), 164.45 (C-7), 170.45 (C-2) ppm. FT-IR (ATR): $\tilde{\nu}$ = 2920 (s), 2851 (s), 1591 (m), 1548 (s), 1501 (s), 1382 (s), 1341 (m), 1254 (s), 1223 (m), 1184 (m), 1155 (m), 1114 (m), 1094 (s), 1963 (m), 1029 (s), 1006 (w), 929 (w), 865 (w), 828 (m), 757 (w), 721 (w), 638 (s), 609 (w), 573 (w), 517 (w), 495 (w) cm^{-1}. MS (ESI): *m/z* = 1007.72 [M$^+$], 1061.72 [M + OMe + Na$^+$]. HRMS (ESI): for $C_{63}H_{107}S_4^+$ calc.: 1007.7199 found: 1007.7168. CHN: calc. $C_{64}H_{107}F_3O_4S_5$ (1157.85): C 66.39, H 9.32, S 13.84; found: C 66.91, H 9.38, S 13.23.

2-(3,4-Bis(dodecylthio)phenyl)-6,7-bis(dodecylthio)chromenium triflate (S_2-Fla-S_2)

Synthesis according to GP 3: phenol **11b** (172 mg, 348 µmol), ethynylketone **10b** (184 mg, 348 µmol), EtOAc (10 mL), yield: 47 mg, 40 µmol, 11 %, purple solid.

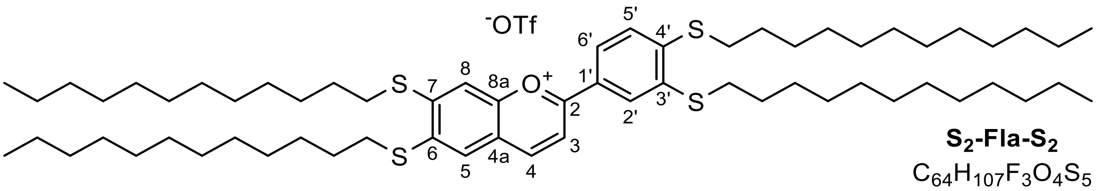

S_2-Fla-S_2
$C_{64}H_{107}F_3O_4S_5$
(1157.85)

^{1}H NMR (700 MHz, $CDCl_3$): δ = 0.84–0.91 (m, 12H, CH_3), 1.13–1.95 (m, 72H), 2.92–2.98 (m, 2H, 4'-SCH_2), 3.11–3.17 (m, 4H, 5-SCH_2, 6-SCH_2), 3.38 (t, J = 7.2 Hz, 2H, 7-SCH_2), 7.08–7.16 (m, 1H, 5'-H), 7.42–7.49 (m, 1H, 5-H), 7.77–7.85 (m, 1H, 2'-H), 8.09–8.15 (m, 1H, 8-H), 8.23–8.30 (m, 1H, 6'-H), 8.37–8.45 (m, 1H, 3-H), 8.77–8.87 (m, 1H, 4-H) ppm. ^{13}C NMR (176 MHz, $CDCl_3$) δ = 14.13 (CH_3), 22.71, 27.49, 27.95, 27.99, 28.49, 28.88, 28.92, 29.14, 29.23, 29.29, 29.33, 29.35, 29.39, 29.51, 29.56, 29.57, 29.61, 29.65, 29.67, 29.71, 29.73, 31.94 (CH_2), 32.58, 33.32, 33.37, 33.47 (SCH_2), 112.75 (C-8), 115.06 (C-3), 121.16 (q, J = 321.9 Hz, CF_3), 121.40 (C-4a), 122.13 (C-5), 123.39 (C-1'), 124.77 (C-5'), 126.06 (C-2'), 127.30 (C-6'), 137.24 (C-3'), 141.31 (C-6), 151.41 (C-4), 152.44 (C-4'), 153.88 (C-8a), 156.68 (C-7), 168.93 (C-2) ppm. FT-IR (ATR): $\tilde{\nu}$ = 2921 (s), 2852 (s), 1607 (m), 1557 (m), 1497 (s), 1457 (s), 1406 (w), 1341 (s), 1257 (s), 1224 (m), 1193 (m), 1159 (m), 1109 (m), 1091 (s), 1029 (s), 972 (w), 939 (w), 871 (w), 815 (w), 741 (w), 721 (w), 638 (s), 572 (w), 517 (w), 445 (w) cm^{-1}. MS (ESI): m/z = 1007.72 [M^+], 1061.72 [M + OMe + Na^+]. HRMS (ESI): for $C_{63}H_{107}S_4^+$ calc: 1007.7199 found: 1007.7174. CHN: calc. for $C_{64}H_{107}F_3O_4S_5$ (1510.44): C 66.39, H 9.32, S 13.84; found: C 66.47, H 9.33, S 13.54.

2-(3,4-Bis(dodecylthio)phenyl)-5,6,7-tris(dodecylthio)chromenium triflate (S_3-Fla-S_2)

Synthesis according to GP 3: phenol **11c** (226 mg, 325 µmol), ethynylketone **10b** (173 mg, 325 µmol), EtOAc (10 mL), yield: 84 mg, 62 µmol, 19 %, brown solid.

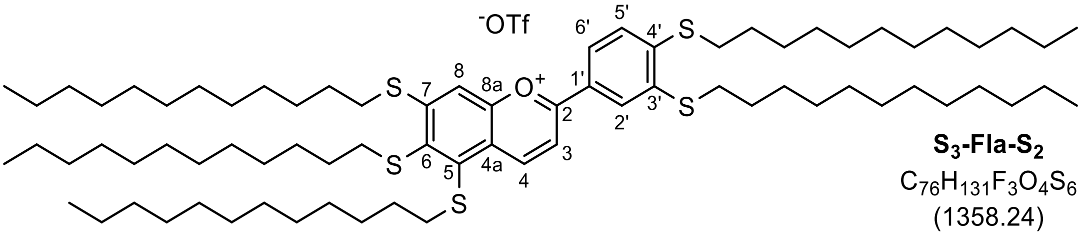

S_3-Fla-S_2
$C_{76}H_{131}F_3O_4S_6$
(1358.24)

^{1}H NMR (700 MHz, $CDCl_3$): δ = 0.84–0.91 (m, 15H, CH_3), 1.11–1.78 (m, 90H, CH_2), 1.86–1.95 (m, 2H, SCH_2CH_2), 2.97 (t, J = 7.5 Hz, 2H, 4'-SCH_2), 3.01 (t, J = 7.5 Hz, 2H, 5-SCH_2), 3.08 (t, J = 7.4 Hz, 2H, 6-SCH_2), 3.11 (t, J = 7.1 Hz, 2H, 3'-SCH_2), 3.26 (t, J = 7.1 Hz, 2H, 7-SCH_2), 7.35 (d, J = 8.5 Hz, 1H, 5'-H), 7.90 (s, 1H, 2'-H), 8.03 (s, 1H, 8-H), 8.43 (d, J = 8.5 Hz, 1H, 6'-H), 8.56 (d,

J = 8.9 Hz, 1H, 3-H), 9.38 (d, *J* = 8.9 Hz, 1H, 4-H) ppm. ^{13}C NMR (176 MHz, $CDCl_3$) δ = 14.13 (CH_3), 22.71, 27.30, 27.99, 28.41, 28.81, 28.89, 29.04, 29.17, 29.20, 29.33, 29.36, 29.37, 29.40, 29.44, 29.54, 29.56, 29.62, 29.66, 29.68, 29.69, 29.71, 29.73, 29.75, 29.81, 29.89, 31.93, 31.95 (CH_2), 32.62 (4'-SCH_2), 33.43 (3'-SCH_2), 34.08 (7-SCH_2), 37.33 (6-SCH_2), 38.95 (5-SCH_2), 112.88 (C-8), 115.44 (C-3), 120.97 (q, *J* = 320.7 Hz, CF_3), 123.44 (C-1'), 124.13 (C-4a), 125.28 (C-5'), 126.51 (C-2'), 128.46 (C-6'), 137.12 (C-3'), 142.17 (C-6), 144.32 (C-5), 152.61 (C-4), 153.21 (C-4'), 156.30 (C-8a), 164.54 (C-7), 169.97 (C-2) ppm. FT-IR (ATR): $\tilde{\nu}$ = 2954 (m), 2920 (s), 2851 (s), 1592 (m), 1546 (s), 1492 (s), 1454 (s), 1405 (w), 1377 (m), 1342 (m), 1252 (s), 1224 (m), 1160 (m), 1060 (s), 1029 (s), 982 (w), 942 (w), 866 (w), 821 (w), 757 (w), 721 (w), 638 (s), 572 (w), 517 (w), 433 (w) cm^{-1}. MS (ESI): *m/z* = 1207.88 [M^+]. HRMS (ESI): for $C_{75}H_{131}S_5^+$ calc.: 1027.8798 found: 1027.8798. CHN: calc. for $C_{76}H_{131}F_3O_4S_6$ (1358.24): C 67.21, H 9.72, S 14.16; found.: C 67.10, H 9.87, S 13.76.

3 Solid State Structure of O_1-V-Fla-S_1

Fortunately, we were able to obtain suitable single crystals of **O_1-V-Fla-S_1** for X-ray crystal structure determination, which enabled a comparison with the known solid-state structure of the oxygen analogue **O_1-V-Fla-O_1**[16] and thus the direct comparison of the S vs. O effect on the solid-state aggregation. The solid-state structure of **O_1-V-Fla-S_1** reveals several common features with the oxygen analogue **O_1-V-Fla-O_1** (Figure S1).

a)

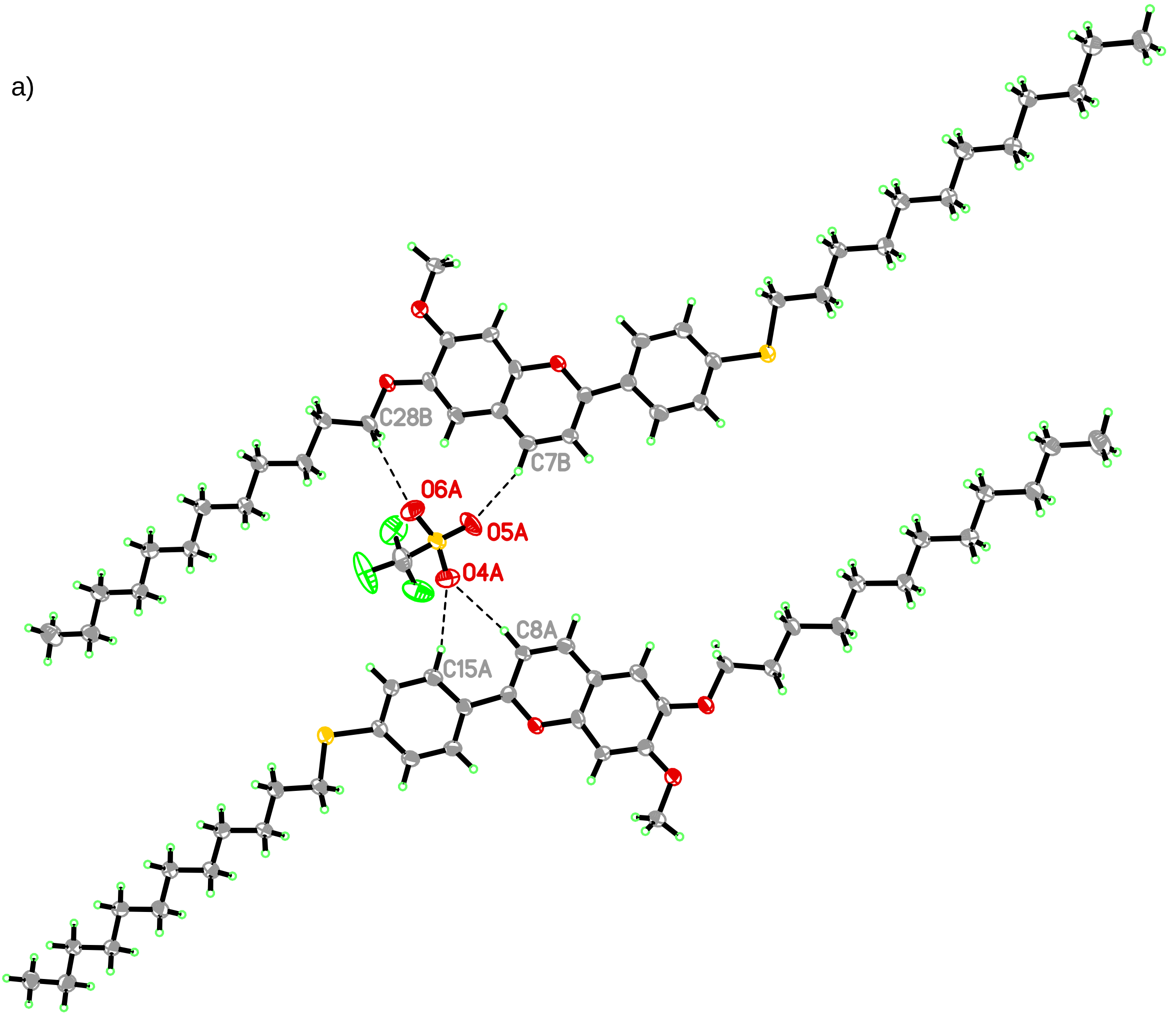

b)

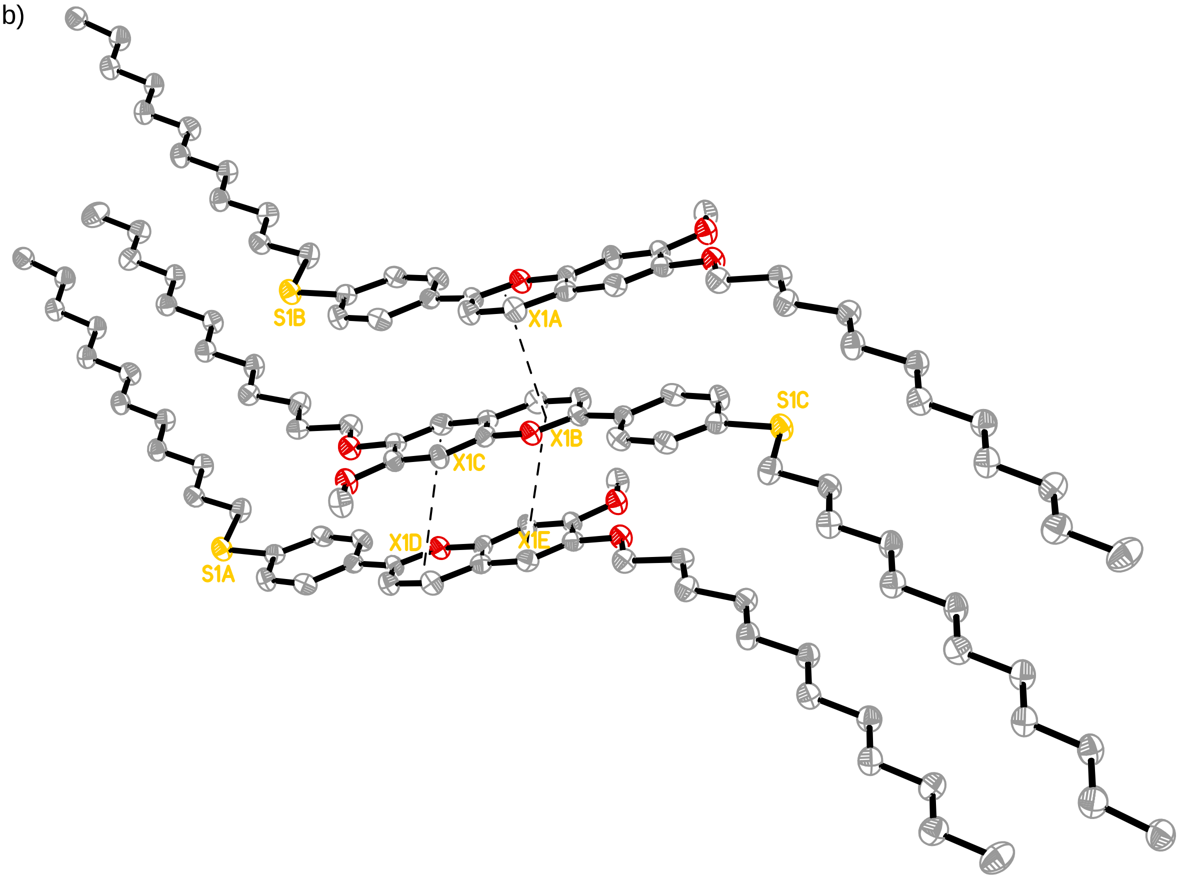

Figure S1 Single-crystal X-ray structure of **O_1-V-Fla-S_1** in the solid state: a) intermolecular hydrogen bonds with triflate and b) π–π interactions between cations.

Compound **O_1-V-Fla-S_1** crystallizes with one ion pair in the asymmetric unit of the centrosymmetric space group P-1. There is no evidence of a sulphur-sulphur interaction in the solid state indicated by an intermolecular cation/cation S-S distance of 7.11 Å and a cation/anion S-S distance of 6.72 Å. The flavylium cation is almost planar with a torsion angle of 6.9(1)° between the AC rings and the phenyl moiety (B-ring). The oxygen atoms of the triflate anion act as acceptors for several hydrogen bond interactions (Figure 2a). First, there are π(C-H) donors of the AC ring and the phenyl moieties. The (H···O) interval of the relevant distances is 2.28 to 2.53 Å. In addition, a weaker interaction is evident with the methyl C-H function of the methoxy group. The (H···O) distances are 2.55 and 2.56 Å, respectively. Finally, there is also a weak interaction between a C-H donor of the alkyl chain and the O6 of the triflate anion with a (H28B···O6) distance of 2.54 Å. The cation built up a layer type stacking interaction with a pairwise 180° rotated orientation of the molecules forced by a slight π-π stacking interaction of the AC cores (Figure 2b). In detail, the pyrylium core interacts with the benzene part of the AC unit and vice versa. The distance of the centroids is in both cases 3.78 Å. Additionally, each pair is also stabilized by a slightly stronger π-π interaction generated only between the pyrylium cores

with a distance of 3.50 Å. Remarkably the phenyl groups of the flavylium moieties are not involved in π-π stacking interactions. In the *bc* view of the packing diagram there is a layer-type orientation of the molecules along the c-axis evident. The centre part of the cation and the triflate anions form a polar layer which alternates with the nonpolar layer consisting the aliphatic interdigitated chains.

Comparison of the thioether-substituted flavylium cations with the alkoxy-substituted flavylium cations are organized in both series in columnar aggregates with antiparallel orientation of the AC rings of intra-columnar neighbors in a similar fashion as was proposed for the H-aggregates in solution (see above) and the stacking found for solid **O_1-V-Fla-O_1**.[16] The most significant difference between S and O derivative is the smaller bond angle of the thioether 4'-S-CH_2 (106.2°) as compared to the alkoxy derivative 4'-O-CH_2 (118.5°).

4 NMR Experiments (O_1-iV-Fla-S_3)

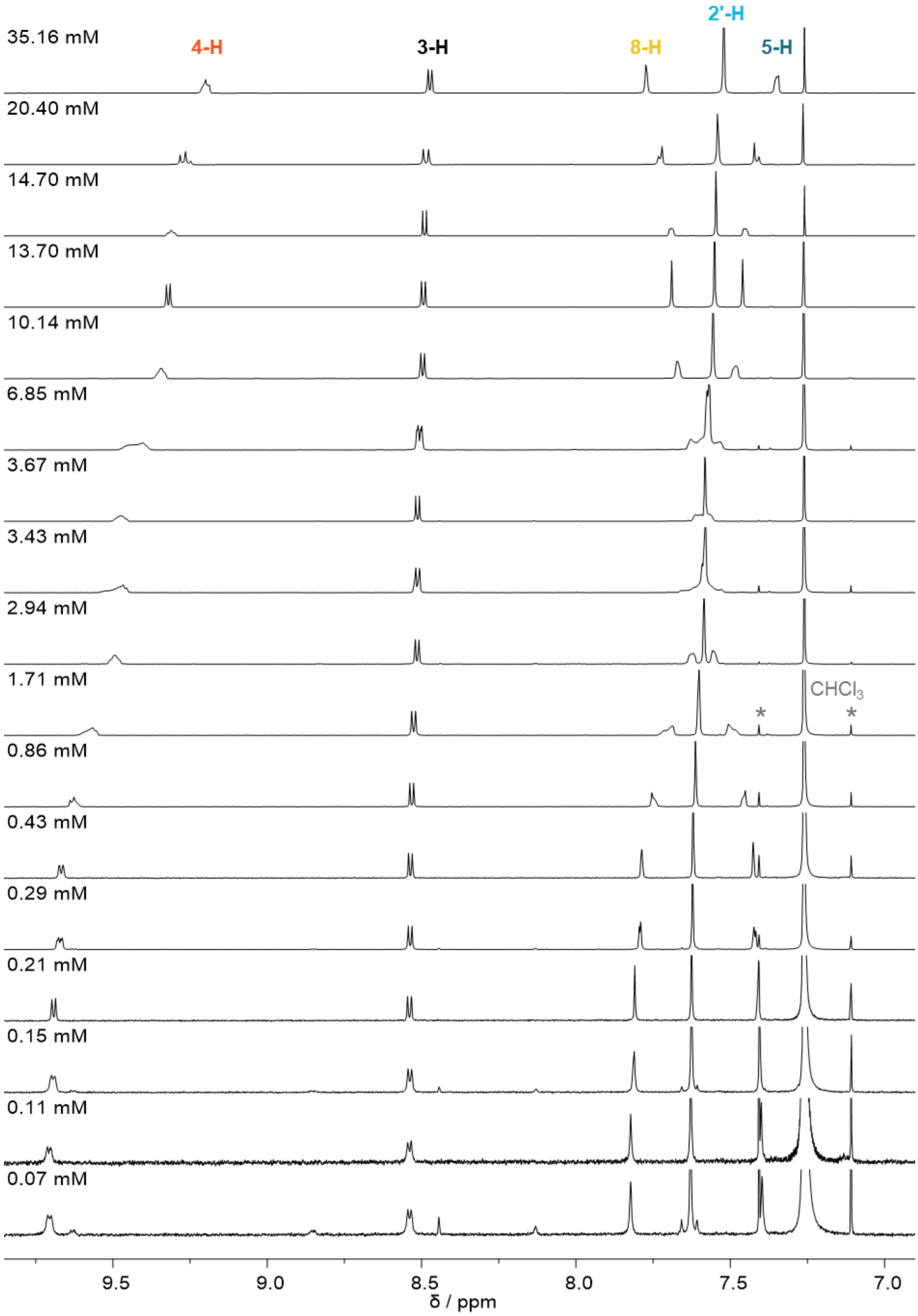

Figure S2 Aromatic signals of the NMR spectra of **O_1-iV-Fla-S_3** at various concentrations in $CDCl_3$ (700 MHz, the ^{13}C satellites are marked by asterisks).

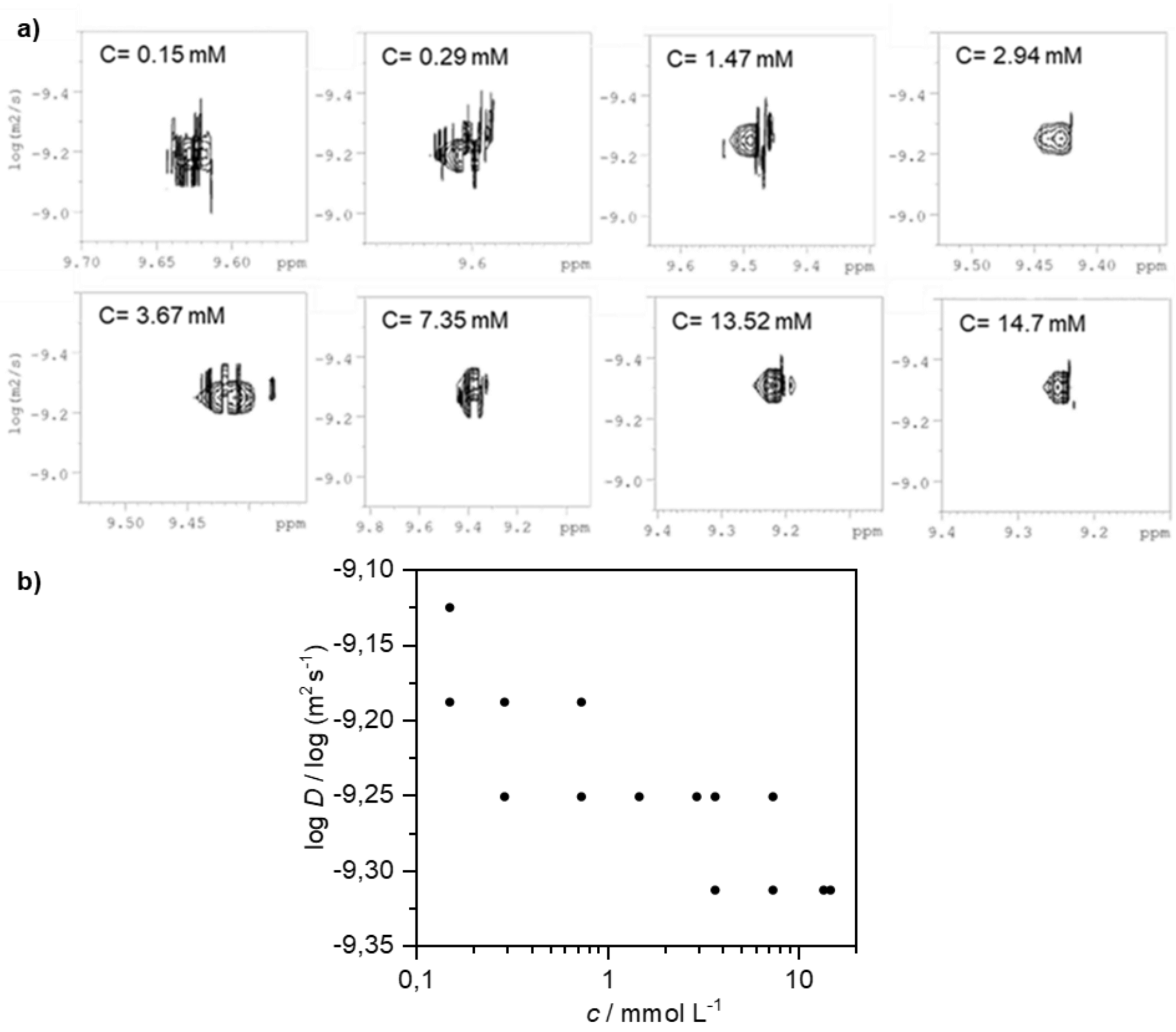

Figure S3 a) DOSY signal of the 4-H proton of **O$_1$-iV-Fla-S$_3$** at different concentrations. The recording was performed with the pulse sequence ledbpgp2s, a gradient time of 2 ms at 5 - 95 % gradient strength and a diffusion time Δ of 50 ms. b) Concentration-dependent diffusion coefficients D of the compound **O$_1$-iV-Fla-S$_3$**.

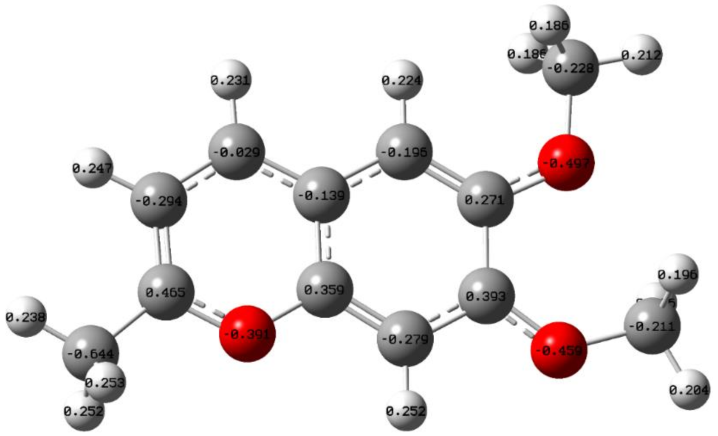

Figure S4 Optimized minimum structure of a substituted a 1-benzopyrylium cation in vacuo. The numerals indicate localized natural charges via natural population analysis (NPA). The DFT calculations were performed at the B3LYP/AUG-cc-pVTZ level of theory (singlet spin state). Light gray: H; dark gray: C; red: oxygen.

Table S1 Optimized geometry of the 1-benzopyrilium cation at B3LYP/AUG-cc-pVTZ level of theory.

atom	x	y	z
C	-1.62410	0.46350	0.00010
C	-0.57660	1.35130	0.00010
C	0.76710	0.90670	0.00000
C	0.99890	0.49120	0.00020
C	-0.03140	1.40390	0.00040
C	-1.35240	0.96030	0.00030
H	1.75060	2.81430	0.00020
H	-0.76340	2.41370	0.00010
C	1.88320	1.74020	0.00010
H	0.16450	2.46550	0.00050
C	3.33640	0.16270	0.00010
C	3.16750	1.20300	0.00000
H	4.03950	1.83780	0.00000
O	2.26980	0.96550	0.00020
O	-2.91860	0.81580	0.00030
O	-2.25160	-1.92880	0.00070
C	-3.69330	1.78830	0.00100
H	-4.02610	-1.26950	-0.89420
H	-4.05270	-2.81160	-0.00120

5 DSC, X-Ray Scattering and UV-VIS Experiments

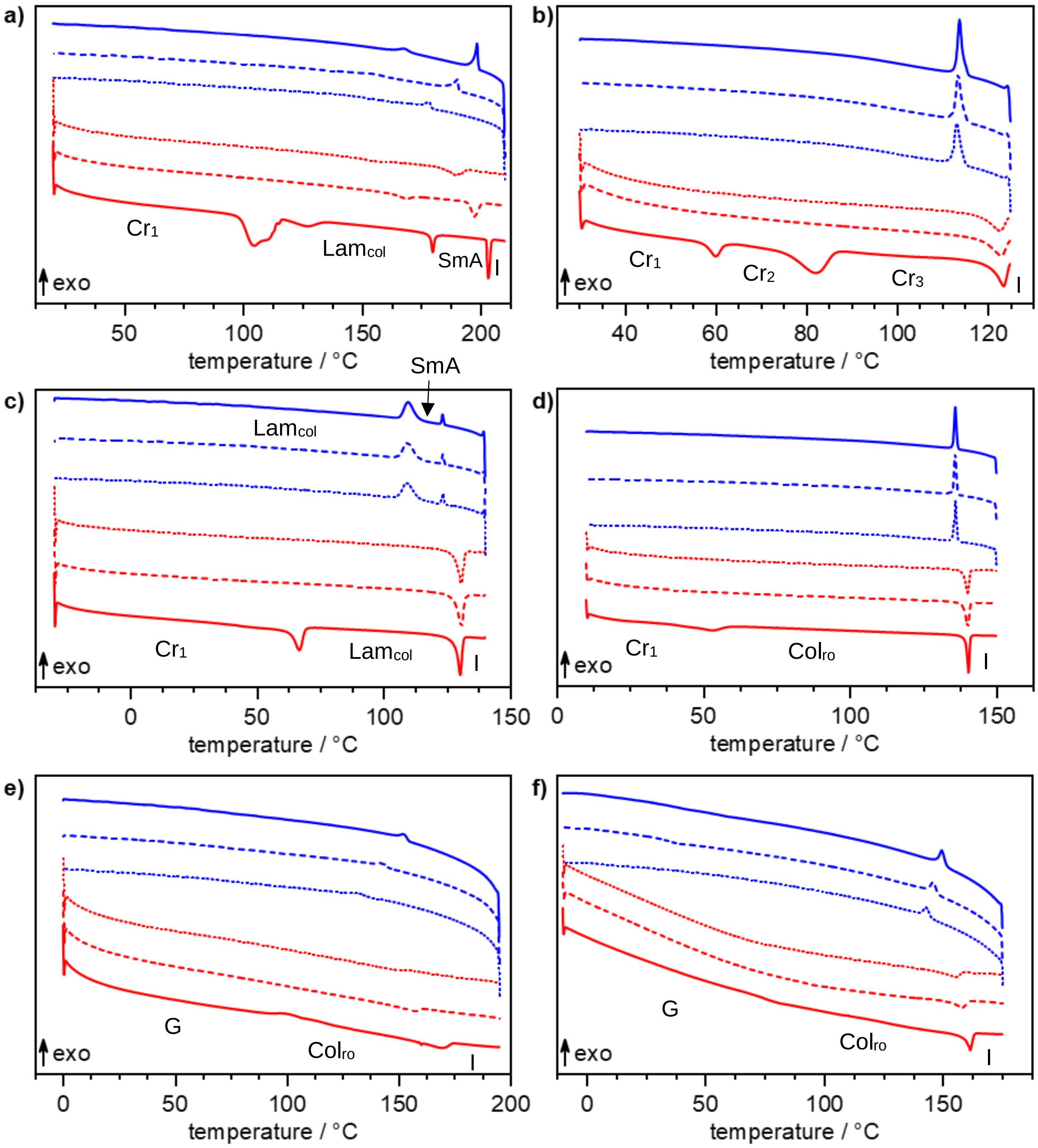

Figure S5 DSC traces of a) **O1-V-Fla-S1**, b) **O1-iV-Fla-S1**, c) **O2-Fla-S1**, d) **O1-V-Fla-S2,** e) **O1-iV-Fla-S2** and f) **O1-iV-Fla-S3** in the 1st (bold), 2nd (dashed), 3rd (dotted) cooling (blue) and heating (red) cycle recorded at a rate of 5 K min^{-1}.

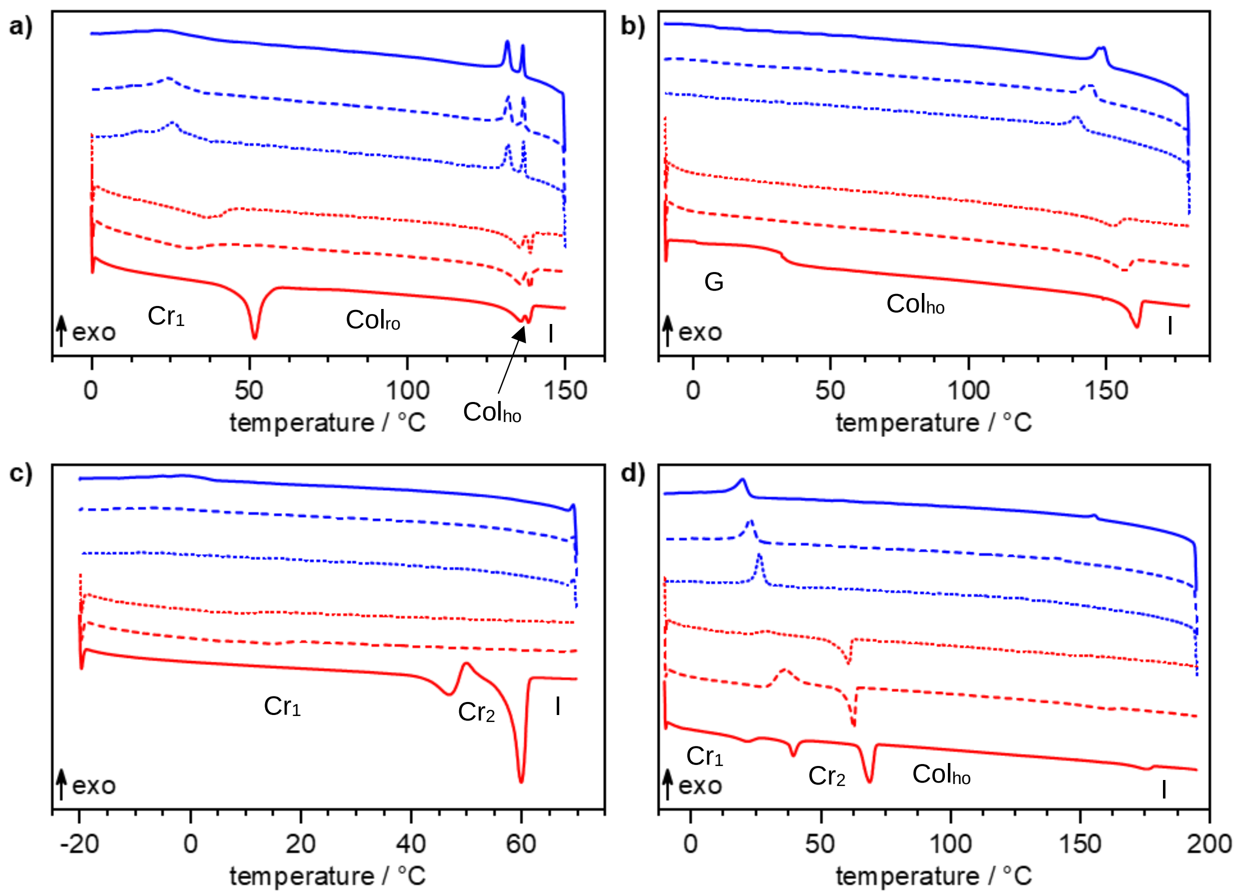

Figure S6 DSC traces of a) **O_2-Fla-S_2**, b) **O_2-Fla-S_3**, c) **O_3-Fla-S_1** and d) **O_3-Fla-S_3** in the 1st (bold), 2nd (dashed), 3rd (dotted) cooling (blue) and heating (red) cycle recorded at a rate of 5 K min^{-1}.

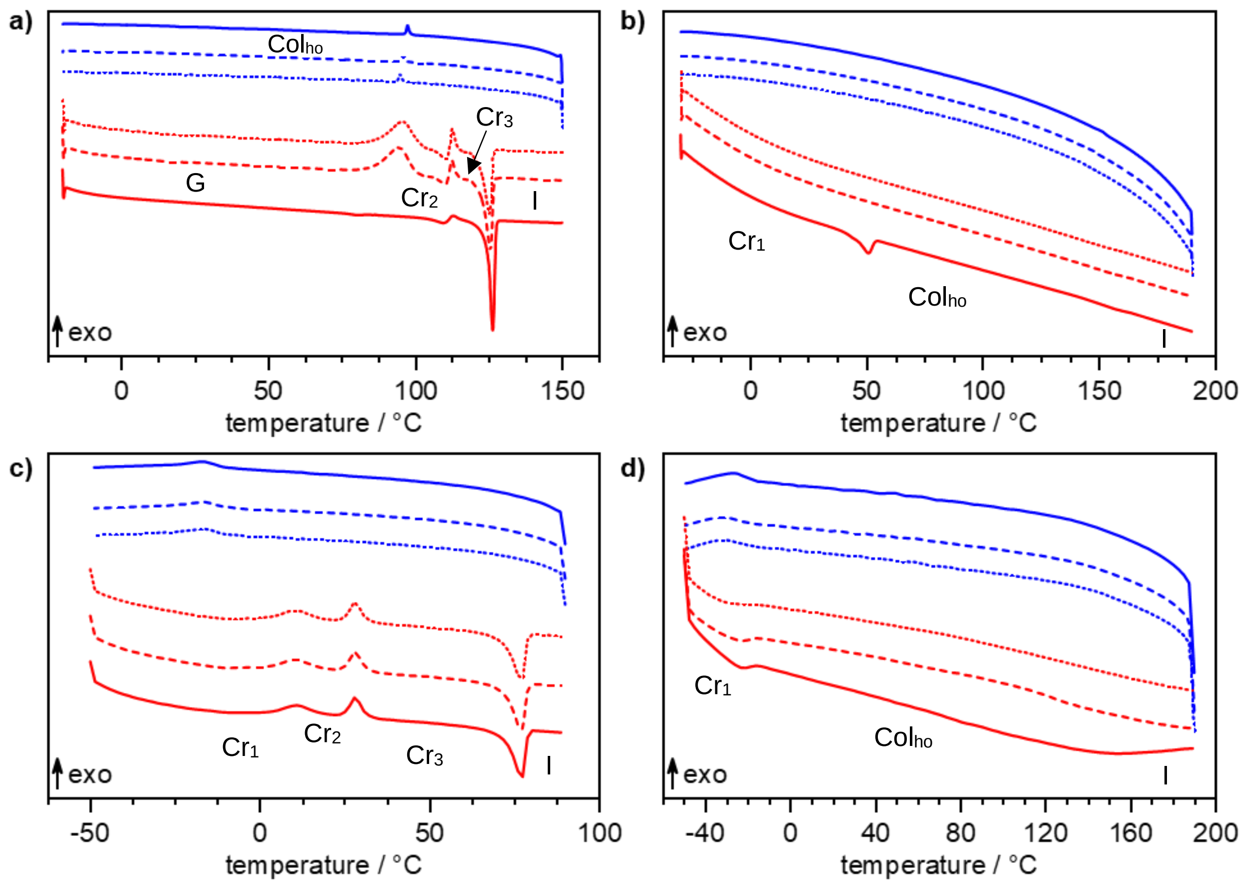

Figure S7 DSC traces of a) **S$_2$-Fla-S$_1$**, b) **S$_2$-Fla-S$_2$**, c) **S$_3$-Fla-S$_1$** and d) **S$_3$-Fla-S$_2$** in the 1st (bold), 2nd (dashed), 3rd (dotted) cooling (blue) and heating (red) cycle recorded at a rate of 5 K min^{-1}.

Table S2 Phase transition temperatures T / °C and enthalpies ΔH / kJ mol^{-1} of the flavylium salts **O$_n$-Fla-S$_m$** and **O$_n$-Fla-S$_m$** during first and second heating/cooling in the DSC (heating/cooling rate: 10 K min^{-1}).

compound		phase [a]	T_1 (ΔH)	phase [a]	T_2 (ΔH)	phase [a]	T_3 (ΔH)	Phase [a]
**O$_1$-V-Fla-S$_1$**	1st H	Cr$_1$[b]	126 (-33.6)	Lam$_{Col}$	179 (-3.1)	SmA	202 (-5.9)	I
	1st C				170 (1.3)		199 (5.2)	
	2nd H				164 (-1.5)		194 (-5.4)	
	2nd C				159 (0.9)		191 (3.7)	
**O$_1$-iV-Fla-S$_1$**	1st H	Cr$_1$	57 (-7.1)	Cr$_2$	75 (-32.0)	Cr$_3$	120 (-13.1)	I
	1st C		134 (3.61)				115 (14.1)	
	2nd H		140 (-4.4)				119 (-11.6)	
	2nd C		132 (0.75)				115 (13.5)	
**O$_2$-Fla-S$_1$**	1st H	Cr$_1$	63 (-16.6)	Lam$_{Col}$	-		128 (-17.3)	I
	1st C			Lam$_{Col}$	114 (12.7) [f]	SmA	124 (1.5)	I
	2nd H				-		128 (-16.7)	
	2nd C				114 (12.7)		123 (1.5)	
**O$_1$-V-Fla-S$_2$**	1st H	Cr$_1$	43 (18.3)	Col$_{ro}$			139 (-17.7)	I
	1st C						137 (16.8)	
	2nd H						139 (-19.9)	
	2nd C						137 (15.9)	
**O$_1$-iV-Fla-S$_2$**	1st H	G	98 (-)	Col$_{ro}$			154 (-11.4)	I

	1st C						155 (2.3)	
	2nd H						142 (-5.4)	
	2nd C						145 (2.02)	
**O$_1$-iV-Fla-S$_3$**	1st H	G	80 (-)[c]	Col$_{ro}$			159 (-18.1)	I
	1st C						152 (12.4)	
	2nd H						151 (-13.5)	
	2nd C						147 (9.1)	
**O$_2$-Fla-S$_2$**	1st H	Cr$_1$	49 (-30.6)	Col$_{ro}$	131 (-)	Col$_{ho}$	138 (-13.9)	I
	1st C		24		131 (6.4)		137 (3.1)	
	2nd H		31		131 (-5.7)		138 (-2.3)	
	2nd C		24		133 (6.3)		138 (3.2)	
**O$_2$-Fla-S$_3$**	1st H	G	35			Col$_{ho}$	157 (-23.4)	I
	1st C						151 (15.7)	
	2nd H						149 (-17.8)	
	2nd C						147 (12.1)	
**O$_3$-Fla-S$_1$**	1st H	Cr$_1$	42 (-25.9)	Cr$_2$			58 (-69.3)	I
	1st C							
	2nd H							
	2nd C							
**O$_3$-Fla-S$_3$**	1st H	Cr$_1$	15 (-6.0)[d]	Cr$_2$	65 (-26.8)	Col$_{ho}$	168 (-4.5)	I
	1st C				23 (15.3)		143 (1.3)	
	2nd H		31 (24)[e]		60 (-21)		157 (-2.3)	
	2nd C				26 (16.9)		144 (0.98)	
**S$_2$-Fla-S$_1$**	1st H	Cr$_1$					124 (-50.3)	I
	1st C					Col$_{ho}$[f]	98 (1.9)	
	2nd H	G	86 (41.4)[e]	Cr$_2$	111 (8.0)[f]	Cr$_3$	122 (40.7)	
	2nd C					Col$_{ho}$[f]	97 (1.8)	
**S$_2$-Fla-S$_2$**	1st H	Cr$_1$	44 (-28.8)			Col$_{ho}$	172[c]	I
	1st C							
	2nd H							
	2nd C							
**S$_3$-Fla-S$_1$**	1st H	Cr$_1$	7 (3.4)	Cr$_2$	25 (14.7)	Cr$_3$	72 (-40.3)	I
	1st C		-13 (2.4)					
	2nd H		7 (3.4)		25 (14.4)		72 (-38.7)	
	2nd C		-13 (1.4)					
**S$_3$-Fla-S$_2$**	1st H	Cr$_1$	-24 (-17.5)	Col$_{ho}$			172[c]	I
	1st C		-30 (8.7)					
	2nd H		-26 (-13.6)					
	2nd C		-33 (6.4)					

[a] The following phases could be observed: Cr (crystalline), G (glass), SmA (smectic A), Lam$_{Col}$ (lamello-columnar), Col$_{ro}$ (ordered columnar rectangular), Col$_{ho}$ (ordered columnar hexagonal), I (isotropic). [b] Additional Cr-Cr-transition during the first heating at 99 °C (33.6 kJ mol^{-1}) [c] Phase transition determined by POM. [d] additional crystal-crystal-transition at 38 °C (-8.8 kJ mol^{-1}). [e] cold crystallization. [f] monotropic behavior.

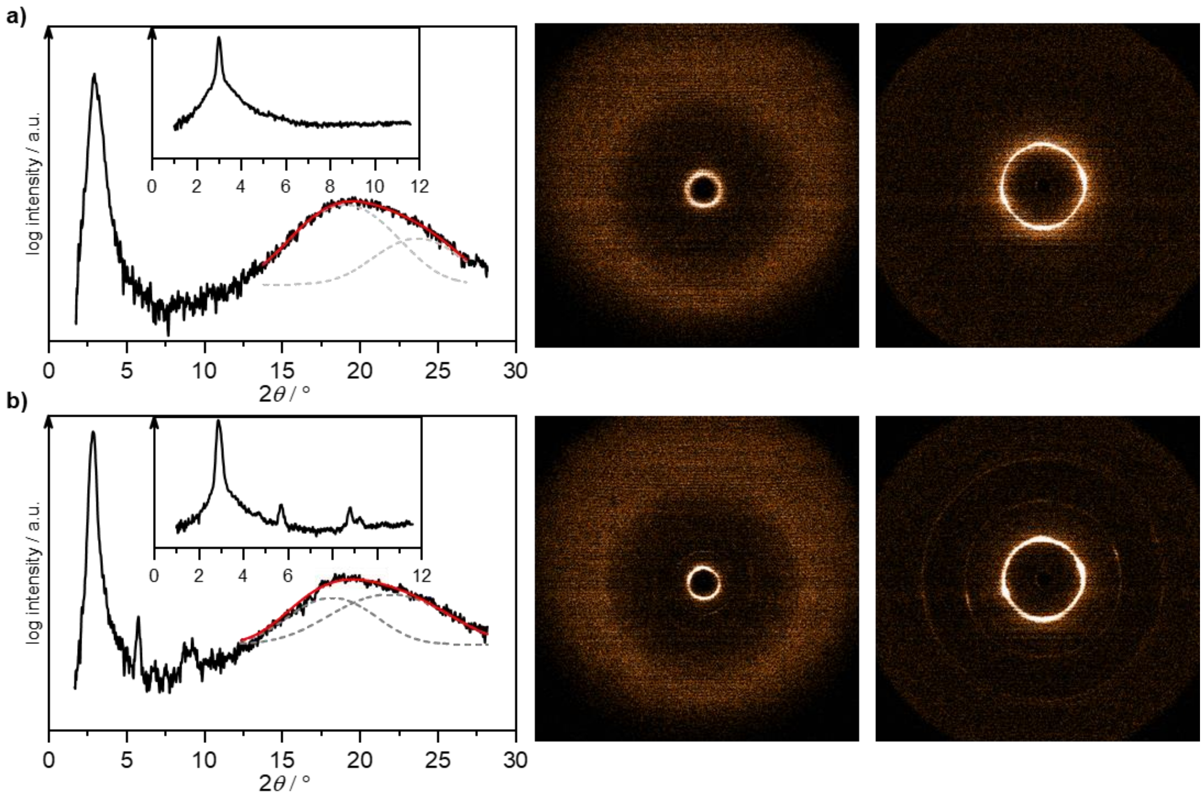

Figure S8 WAXS and SAXS (inset) diffractograms and the corresponding patterns (left: WAXS, right: SAXS) of **O_2-Fla-S_1** in a) the SmA phase at 120 °C and b) the Lam_{Col} phase at 90 °C.

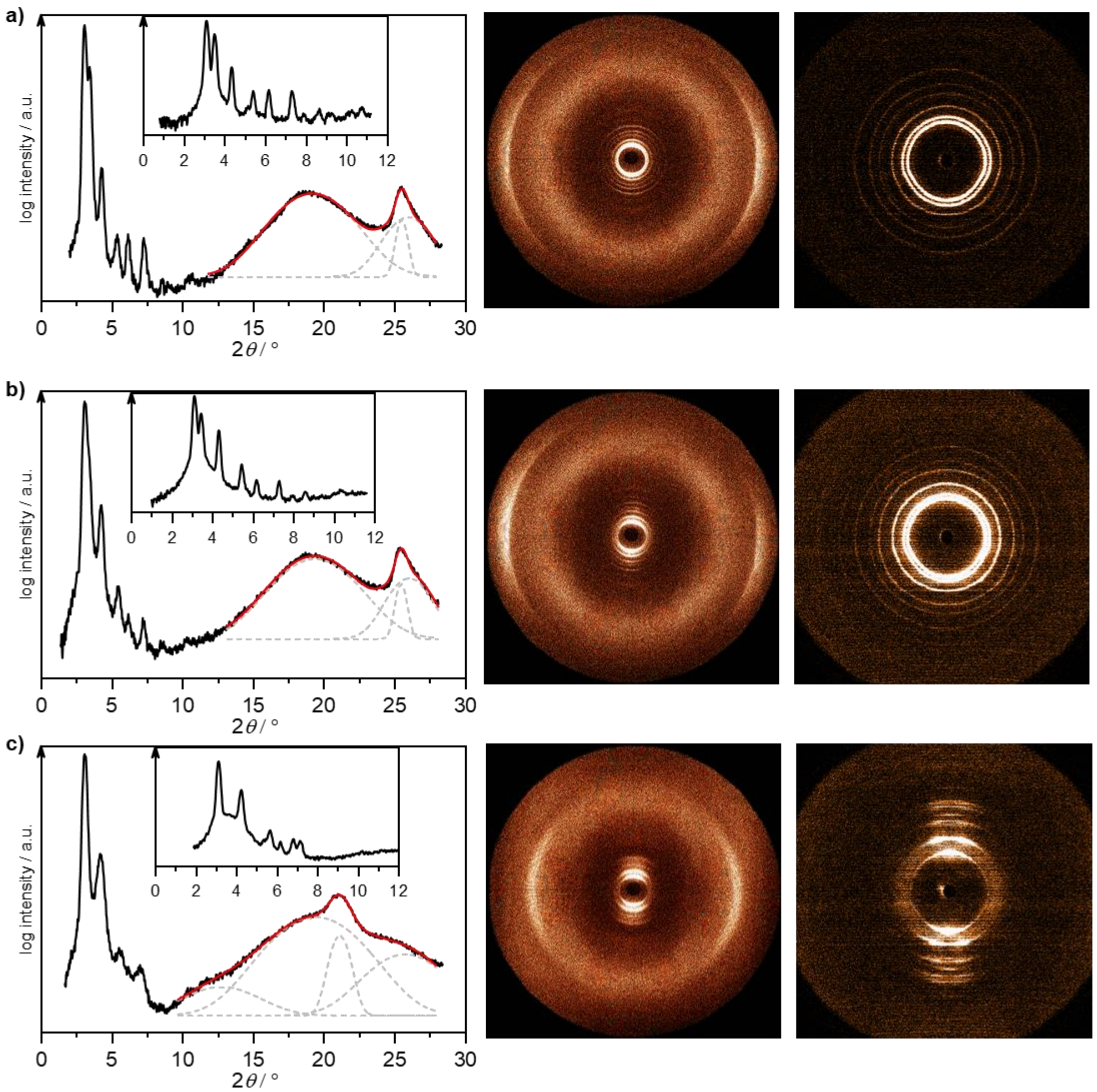

Figure S9 WAXS and SAXS (inset) diffractograms and the corresponding patterns (left: WAXS, right: SAXS) of fibre samples of a) **O_1-V-Fla-S_2** at 120°C, b) **O_1-iV-Fla-S_2** at 100 °C and c) **O_1-iV-Fla-S_3** at 100 °C.

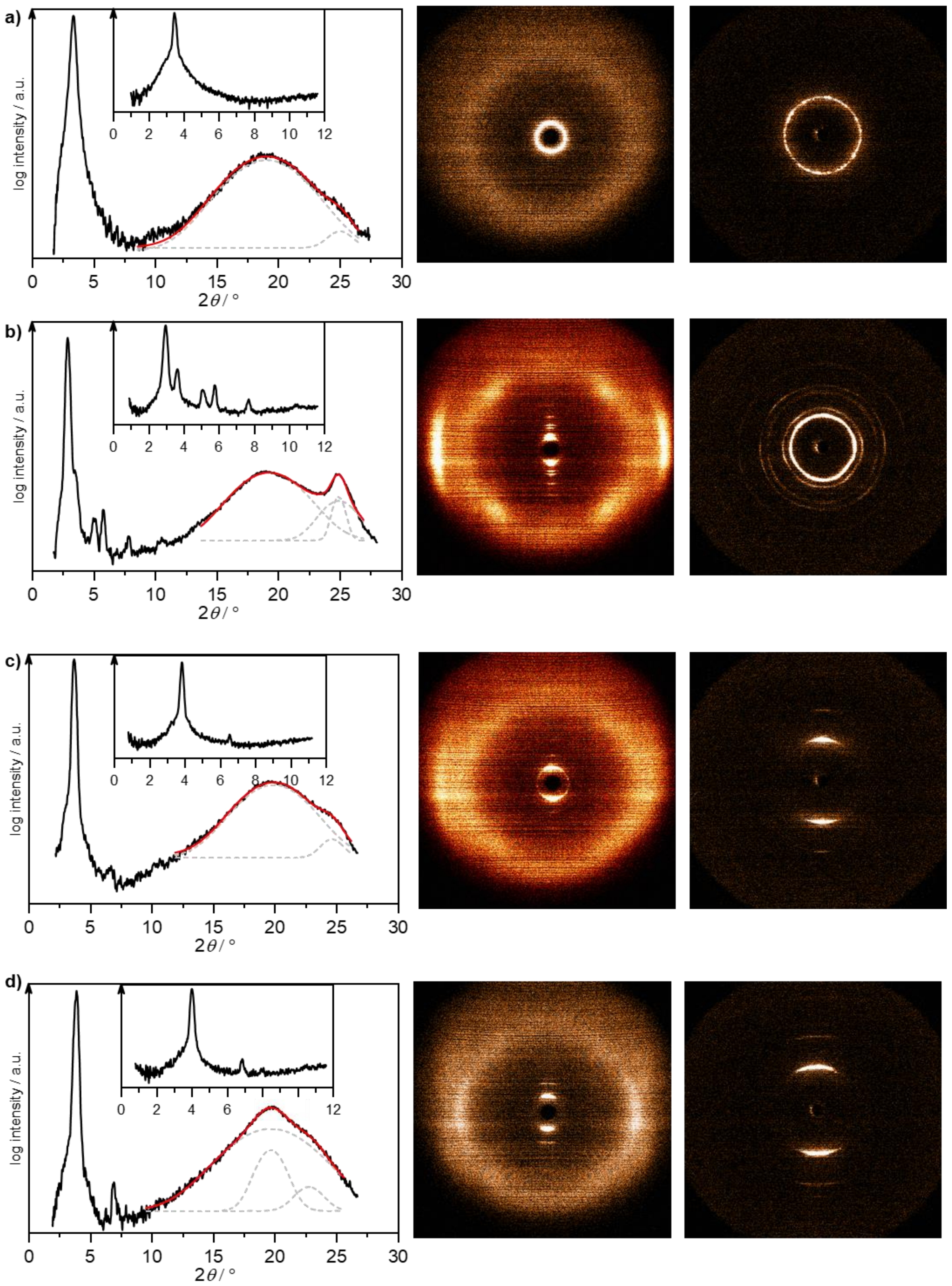

Figure S10 WAXS and SAXS (inset) diffractograms and the corresponding patterns (left: WAXS, right: SAXS) of fibre samples of a) **O2-Fla-S2** in the Col_{ho} phase at 135 °C and b) in the Col_{ro} phase at 100 °C. c) **O2-Fla-S3** and d) **O3-Fla-S3** at 100 °C.

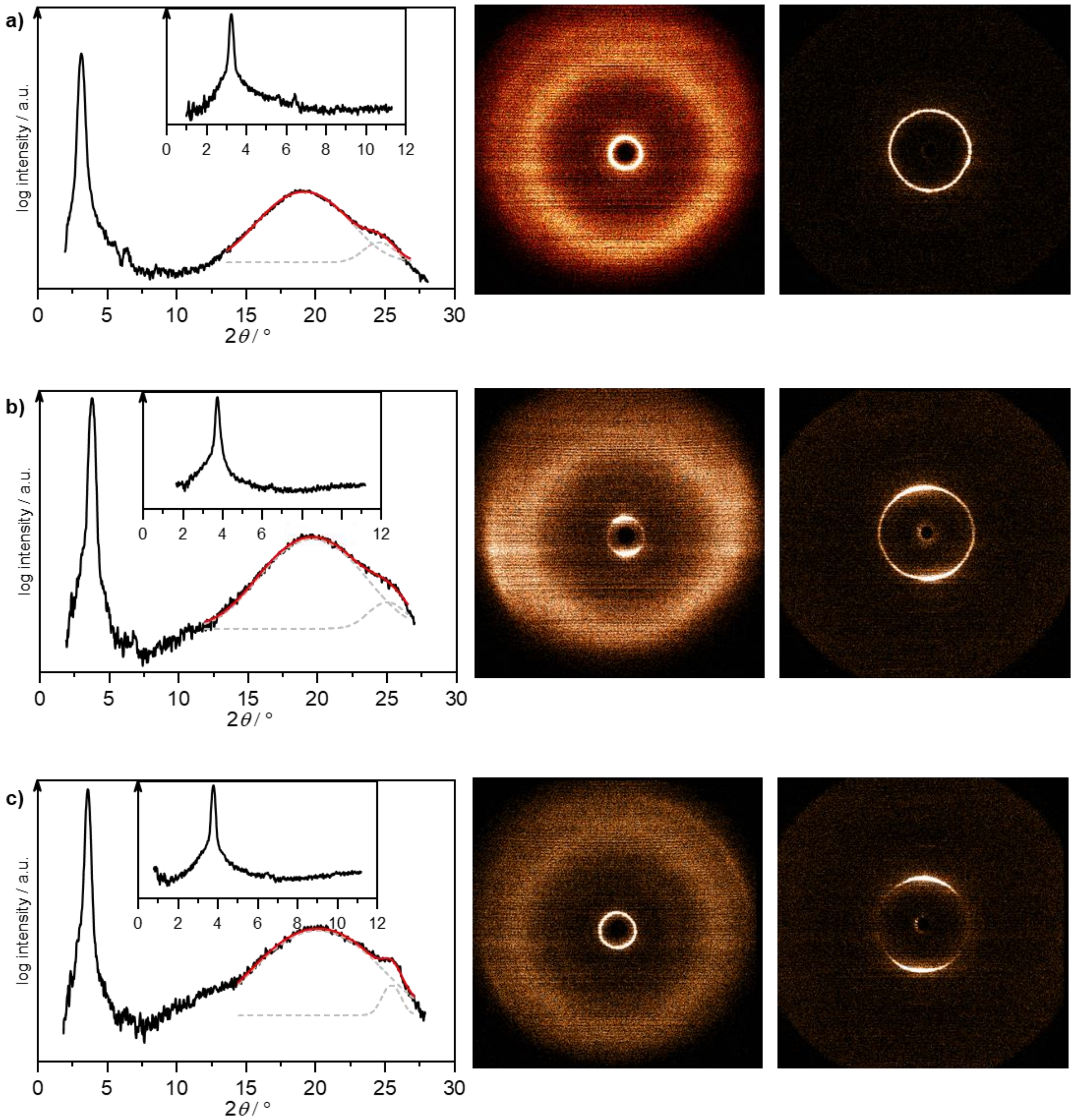

Figure S11 WAXS and SAXS (inset) diffractograms and the corresponding patterns (left: WAXS, right: SAXS) of fibre samples of a) **S_2-Fla-S_1** at 60 °C, b) **S_2-Fla-S_2** at 90 °C and c) **S_3-Fla-S_2** at 100 °C.

Table S3 Detailed unit cell parameters.

ILC	Z	$d_{\pi\text{-}\pi}$ [Å]	d_{halo} [Å]	ρ [g/cm^3] assuming $d_{\pi\text{-}\pi}$
**O_2-Fla-S_2**	2	3.56	4.66	1.31
**O_2-Fla-S_3**	1	3.62	4.46	0.99
**O_3-Fla-S_3**	1	3.9	4.51	1.13
**S_2-Fla-S_1**	2	3.62	4.62	1.029
**S_2-Fla-S_2**	1	3.55	4.51	0.852
**S_3-Fla-S_2**	1	3.49	4.4	1.031

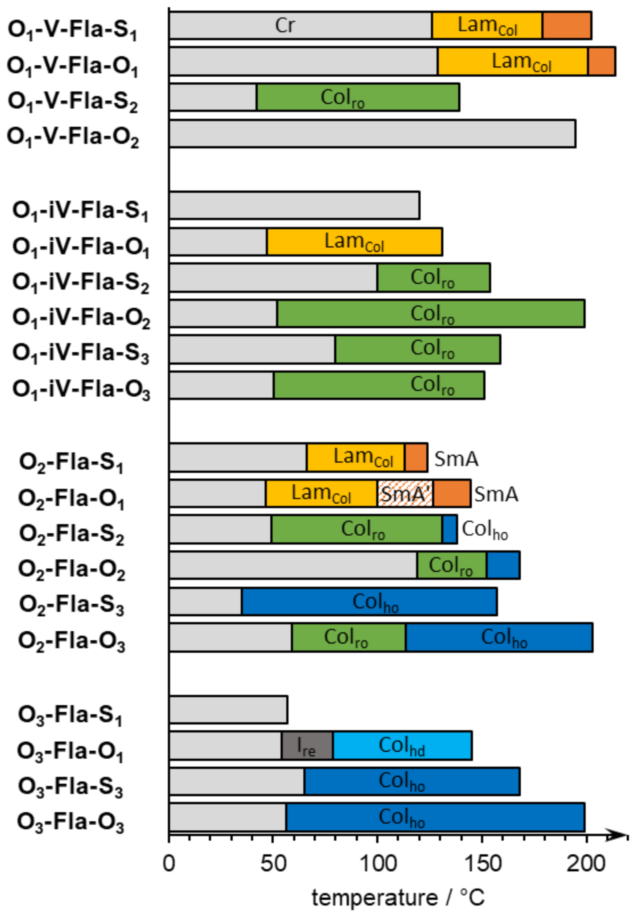

Figure S12 Mesophases of thioether flavylium salts **O_n-Fla-S_m** in comparison to their ether counterparts **O_n-Fla-O_m**. The values for **O_n-Fla-O_m** were taken from ref. [13].

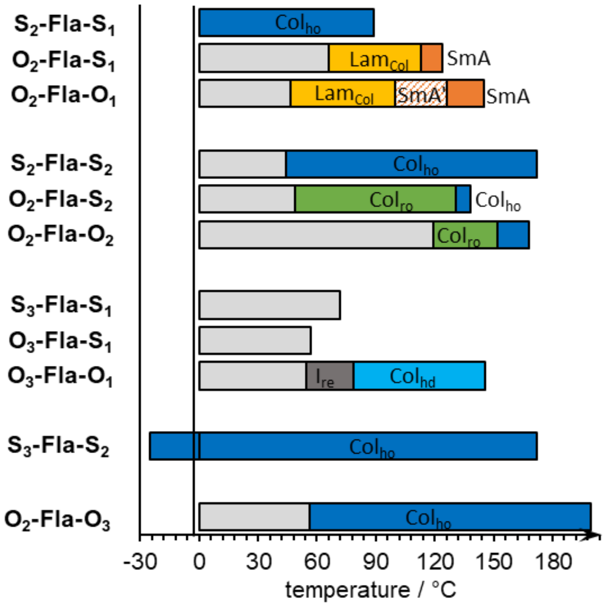

Figure S13 Mesophases of fully thioether substituted flavylium salts **S_n-Fla-S_m** in comparison to their mixed (**O_n-Fla-S_m)** and alkoxy substituted counterparts **O_n-Fla-O_m**. The values for **O_n-Fla-O_m** were taken from ref. [13].

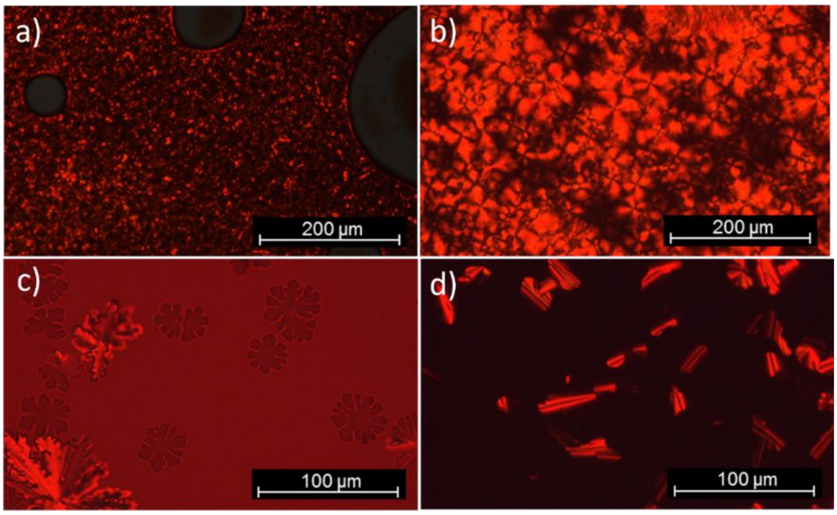

Figure S14 POM textures of **O_2-Fla-S_1**: a) maltese crosses at 88 °C, cooling rate of 5 K min^{-1} b) Textur von **O_2-Fla S_1** e) focal conice textur at 112 °C, cooling rate of 5 K min ^{1}. POM textures of **O_2-Fla-S_2**: c) dentric growth at 142 °C, cooling rate of 2 K min^{-1}. d) fan-ike textures with line effects and large homeotropic areas at 140 °C, cooling rate of 2 K min^{-1}.

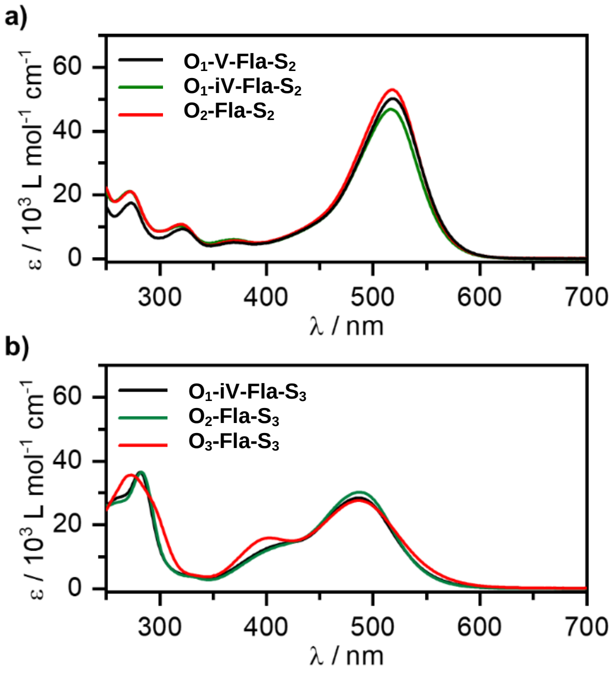

Figure S15 (a) Absorption spectra of **O_n-Fla-S_2** ($c = 2 \cdot 10^{-5}$ mol L^{-1} in $CHCl_3$); (b) absorption spectra of **O_n-Fla-S_3** ($c = 2 \cdot 10^{-5}$ mol L^{-1} in $CHCl_3$).

6 References

[1] AnalySIS 3.2, *Soft Imaging System*, **2002**.

[2] STARe 7.01, *Mettler Toledo*, **2006**.

[3] SAXS 4.1.26, *Bruker*, **2006**.

[4] Datasqueeze 2.2, P. Heiney, **2011**.

[5] OriginPro 9.1.0 Sr2 B271, *OriginLab Cooperation*, **2011**.

[6] N. Godbert, A. Crispini, M. Ghedini, M. Carini, F. Chiaravalloti, A. Ferrise, *J. Appl. Crystallogr.* **2014**, *47*, 668–679.

[7] Gaussian 16, Revision B.01, Frisch, M. J.; Trucks, G. W.; Schlegel, H. B.; Scuseria, G. E.; Robb, M. A.; Cheeseman, J. R.; Scalmani, G.; Barone, V.; Petersson, G. A.; Nakatsuji, H.; Li, X.; Caricato, M.; Marenich, A. V.; Bloino, J.; Janesko, B. G.; Gomperts, R.; Mennucci, B.; Hratchian, H. P.; Ortiz, J. V.; Izmaylov, A. F.; Sonnenberg, J. L.; Williams-Young, D.; Ding, F.; Lipparini, F.; Egidi, F.; Goings, J.; Peng, B.; Petrone, A.; Henderson, T.; Ranasinghe, D.; Zakrzewski, V. G.; Gao, J.; Rega, N.; Zheng, G.; Liang, W.; Hada, M.; Ehara, M.; Toyota, K.; Fukuda, R.; Hasegawa, J.; Ishida, M.; Nakajima, T.; Honda, Y.; Kitao, O.; Nakai, H.; Vreven, T.; Throssell, K.; Montgomery, Jr., J. A.; Peralta, J. E.; Ogliaro, F.; Bearpark, M. J.; Heyd, J. J.; Brothers, E. N.; Kudin, K. N.; Staroverov, V. N.; Keith, T. A.; Kobayashi, R.; Normand, J.; Raghavachari, K.; Rendell, A. P.; Burant, J. C.; Iyengar, S. S.; Tomasi, J.; Cossi, M.; Millam, J. M.; Klene, M.; Adamo, C.; Cammi, R.; Ochterski, J. W.; Martin, R. L.; Morokuma, K.; Farkas, O.; Foresman, J. B.; Fox, D. J. *Gaussian, Inc.*, Wallingford CT, **2016**.

[8] a) B. Miehlich, A. Savin, H. Stoll, H. Preuss, *Chem. Phys. Lett.* **1989**, *157*, 200–206; b) A. D. Becke, *J. Chem. Phys.* **1993**, *98*, 5648–5652.

[9] D. E. Woon, T. H. Dunning, *J. Chem. Phys.* **1995**, *103*, 4572–4585.

[10] A. E. Reed, R. B. Weinstock, F. Weinhold, *J. Chem. Phys.* **1985**, *83*, 735–746.

[11] A. Jankowiak, Ż. Debska, J. Romański, P. Kaszyński, *J. Sulfur Chem.* **2012**, *33*, 1–7.

[12] S. Xia, L. Gan, K. Wang, Z. Li, D. Ma, *J. Am. Chem. Soc.* **2016**, *138*, 13493–13496.

[13] R. Forschner, J. Knelles, K. Bader, C. Müller, W. Frey, A. Köhn, Y. Molard, F. Giesselmann, S. Laschat, *Chem. Eur. J.* **2019**, *25*, 12966–12980.

[14] S. Chassaing, M. Kueny-Stotz, G. Isorez, R. Brouillard, *Eur. J. Org. Chem.* **2007**, *2007*, 2438–2448.

[15] M. Frigerio, M. Santagostino, S. Sputore, *J. Org. Chem.* **1999**, *64*, 4537–4538.

[16] Q. Zheng, G. S. He, P. N. Prasad, *J. Mater. Chem.* **2005**, *15*, 579–587.

7 ^{1}H and ^{13}C NMR spectra

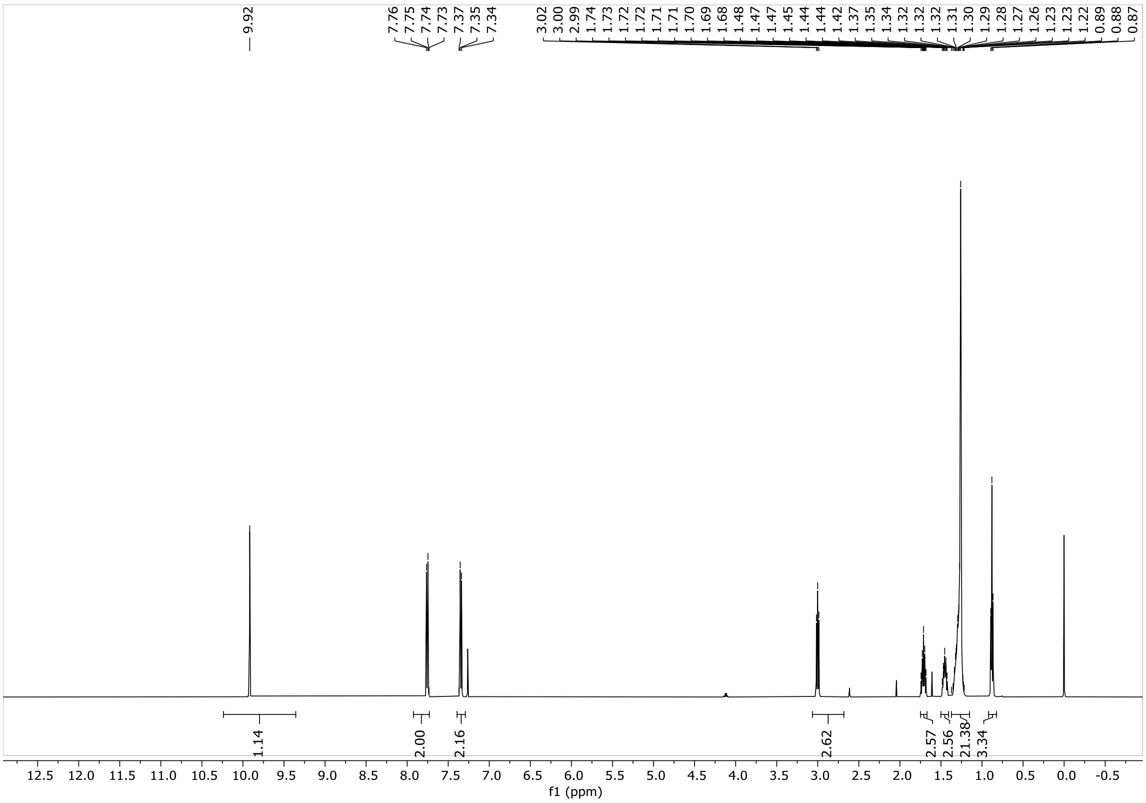

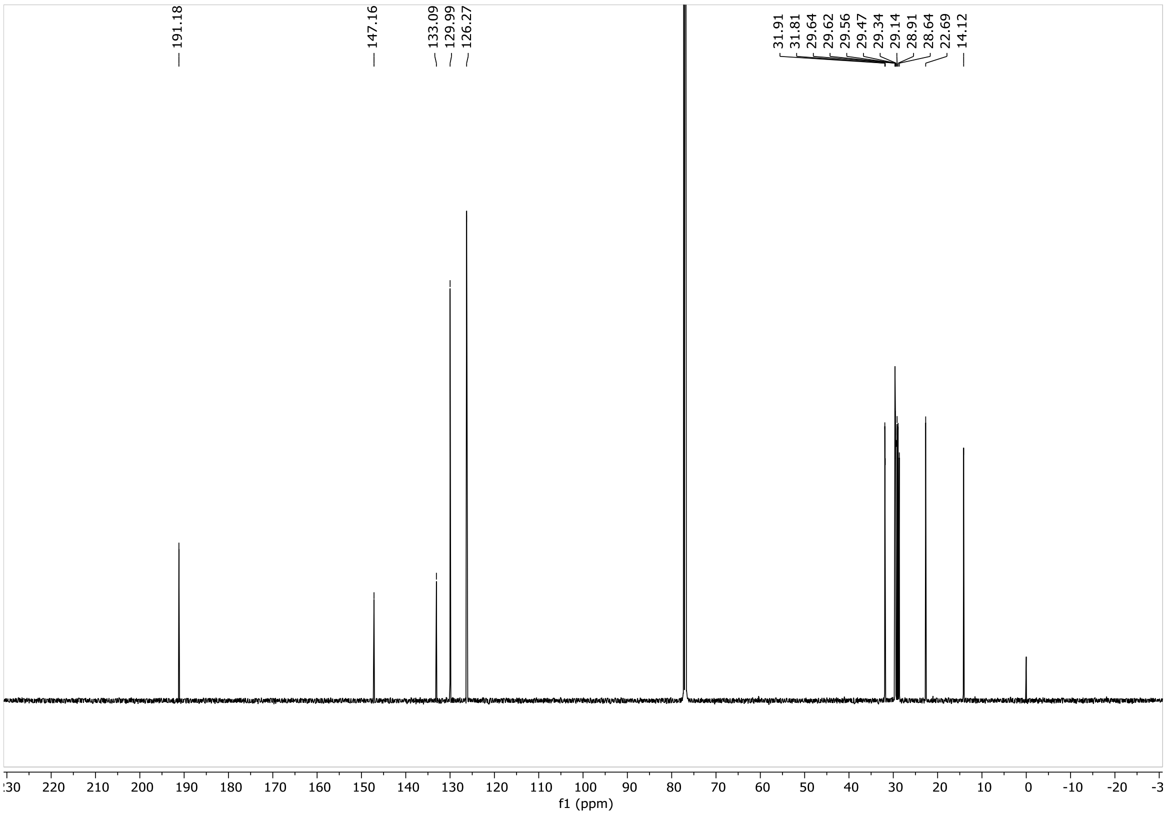

Figure S16: ^{1}H (top**,** at 300 MHz) and ^{13}C NMR (bottom**,** at 75 MHz) of **8a** in $CDCl_3$.

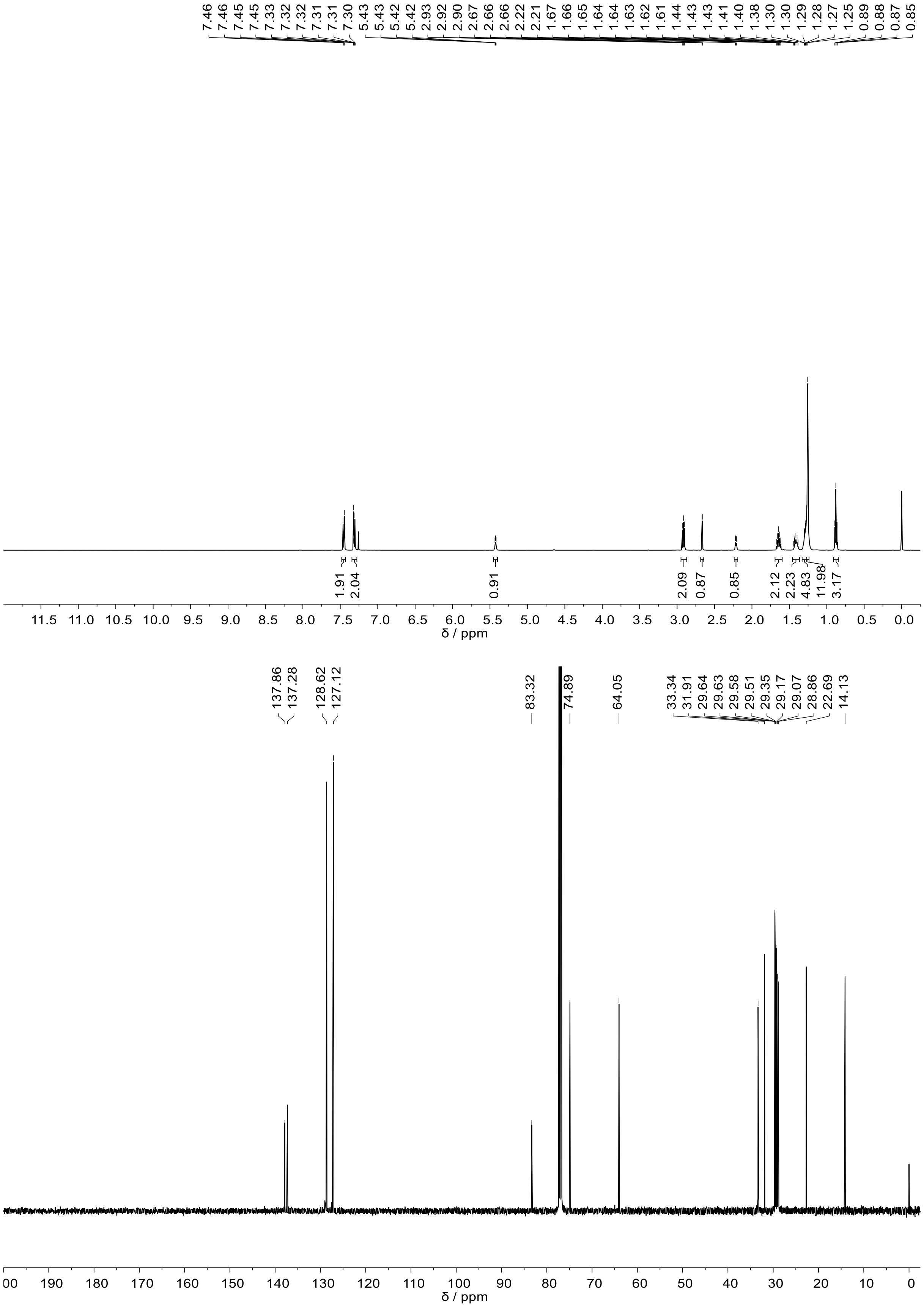

Figure S18: ^{1}H (top, at 500 MHz) and ^{13}C NMR (bottom, at 126 MHz) of **9a** in $CDCl_3$ in $CDCl_3$.

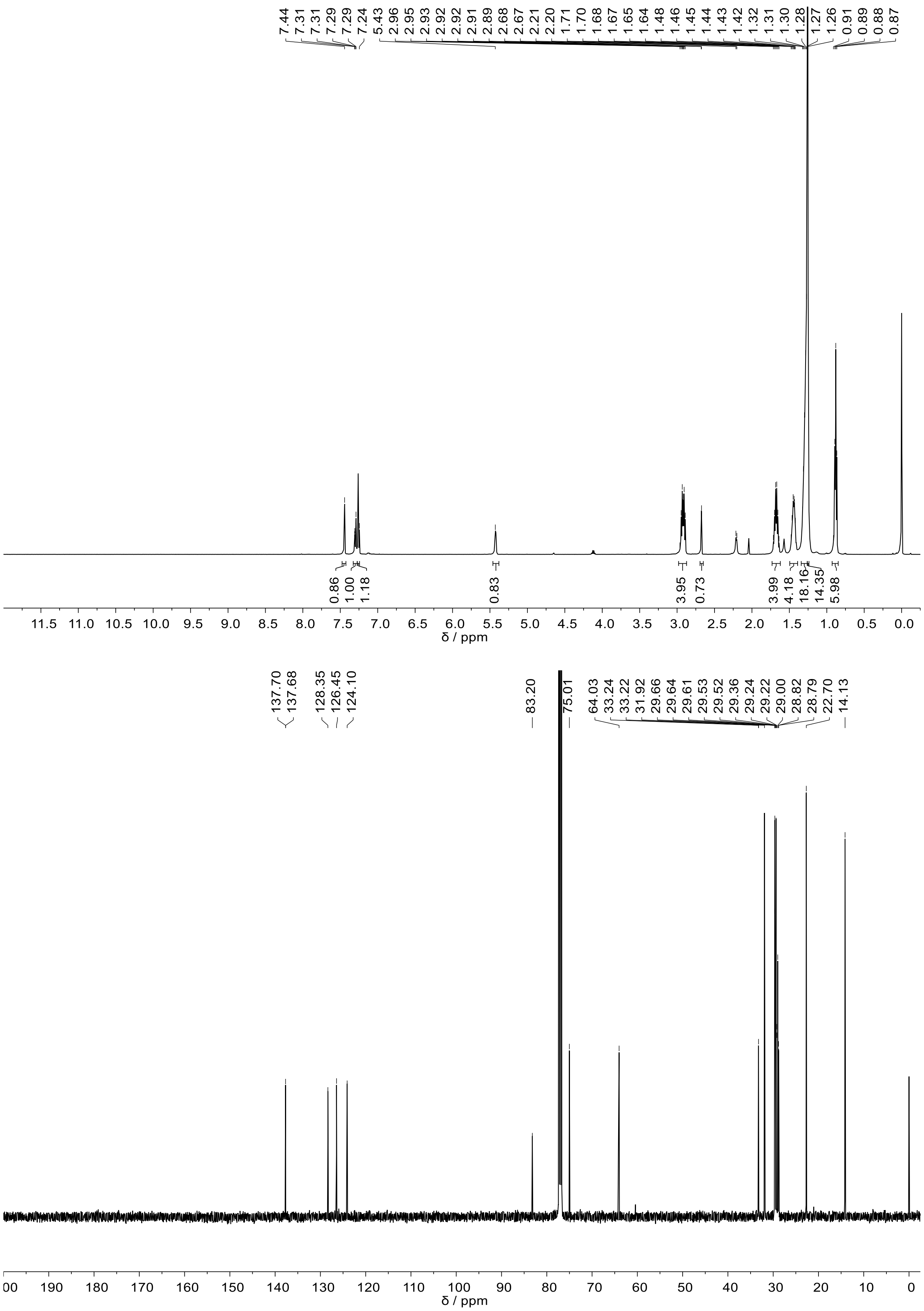

Figure S19: ^{1}H (top, at 500 MHz) and ^{13}C NMR (bottom, at 126 MHz) of **9b** in $CDCl_3$.

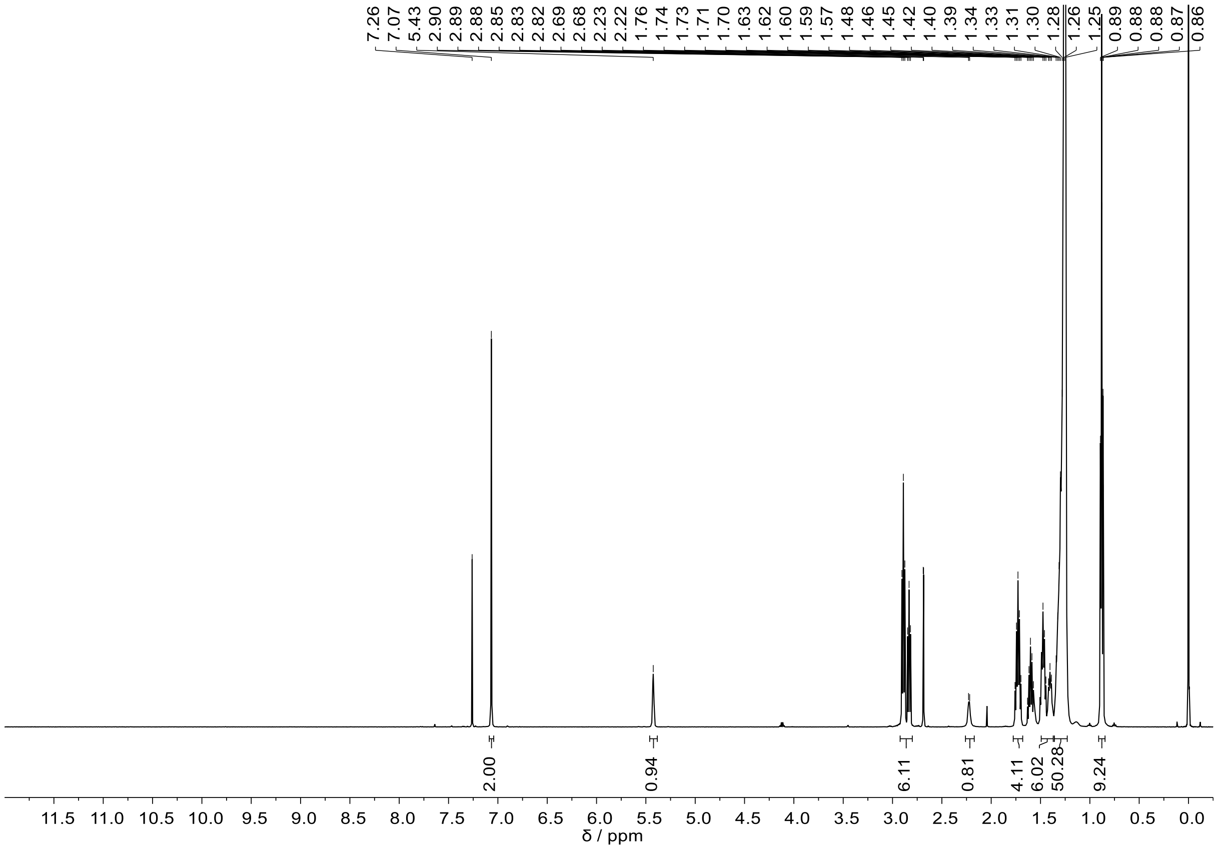

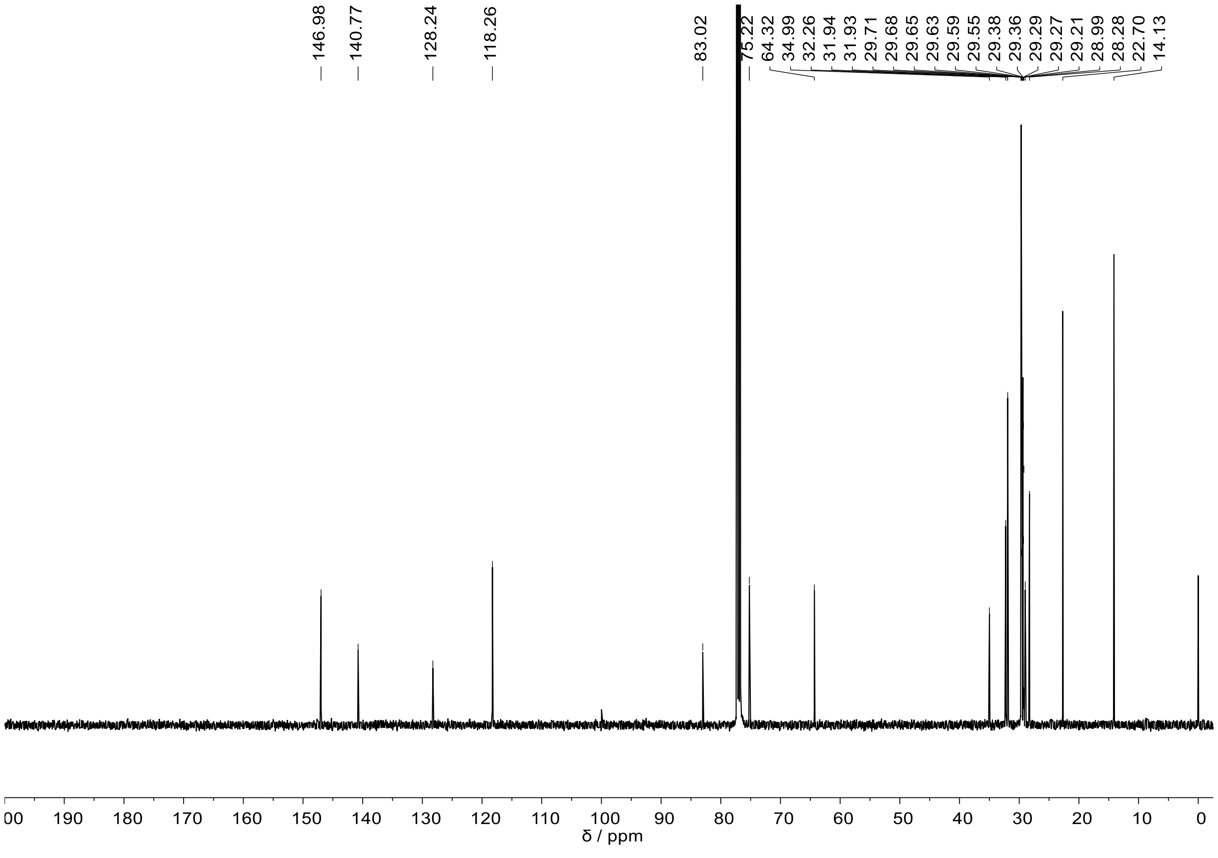

Figure S20: ^{1}H (top, at 500 MHz) and ^{13}C NMR (bottom, at 126 MHz) of **9c** in $CDCl_3$.

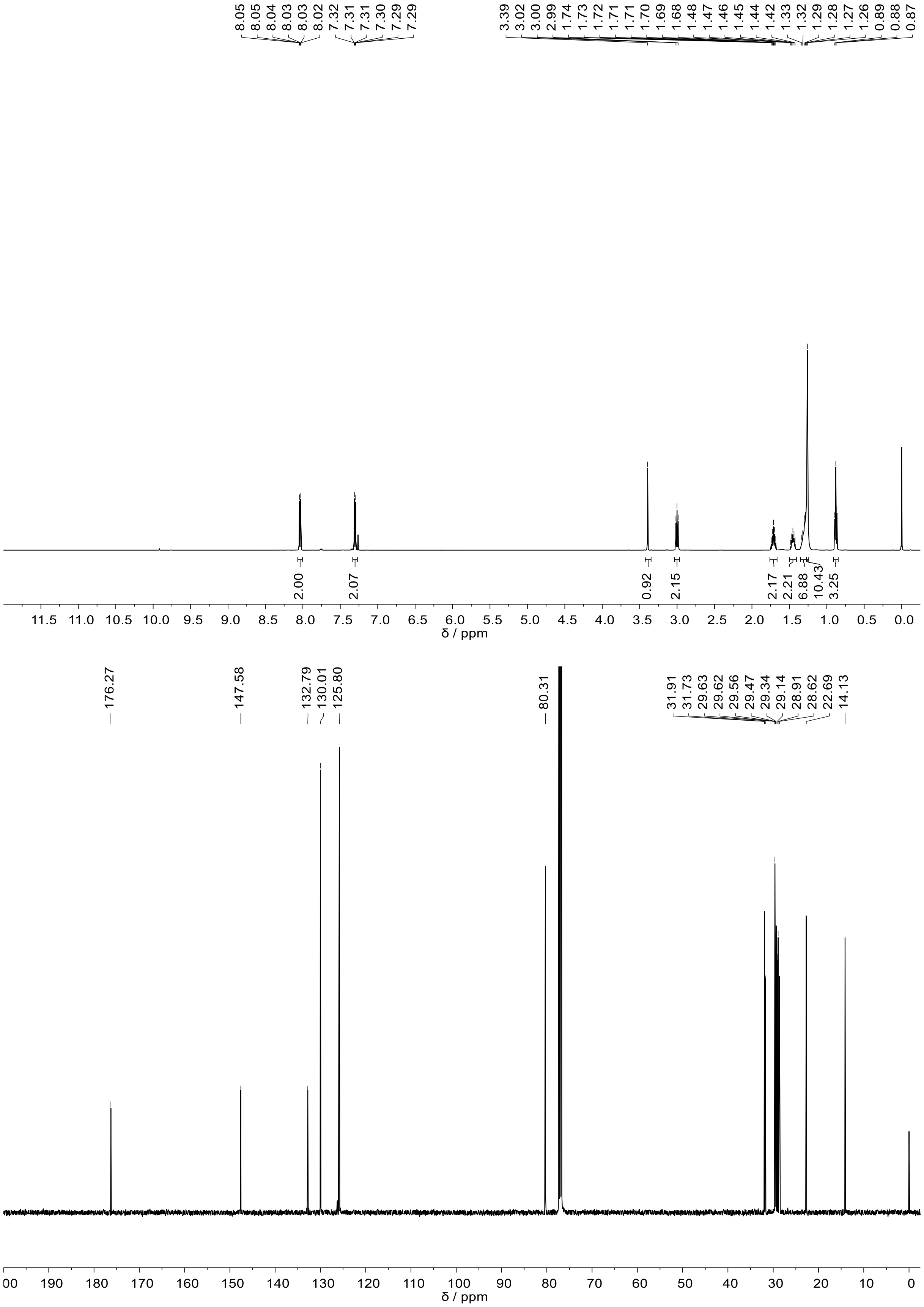

Figure S21: ^{1}H (top, at 500 MHz) and ^{13}C NMR (bottom, at 136 MHz) of **10a** in $CDCl_3$.

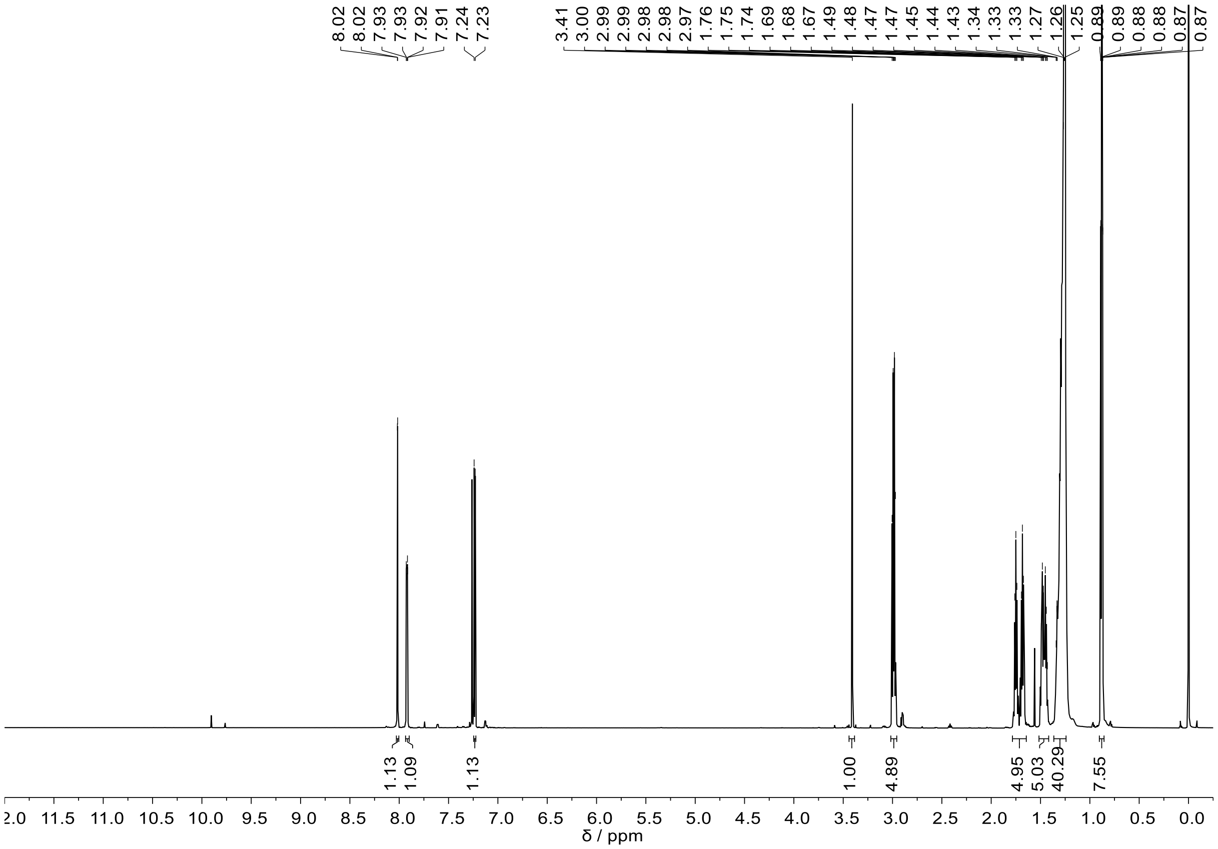

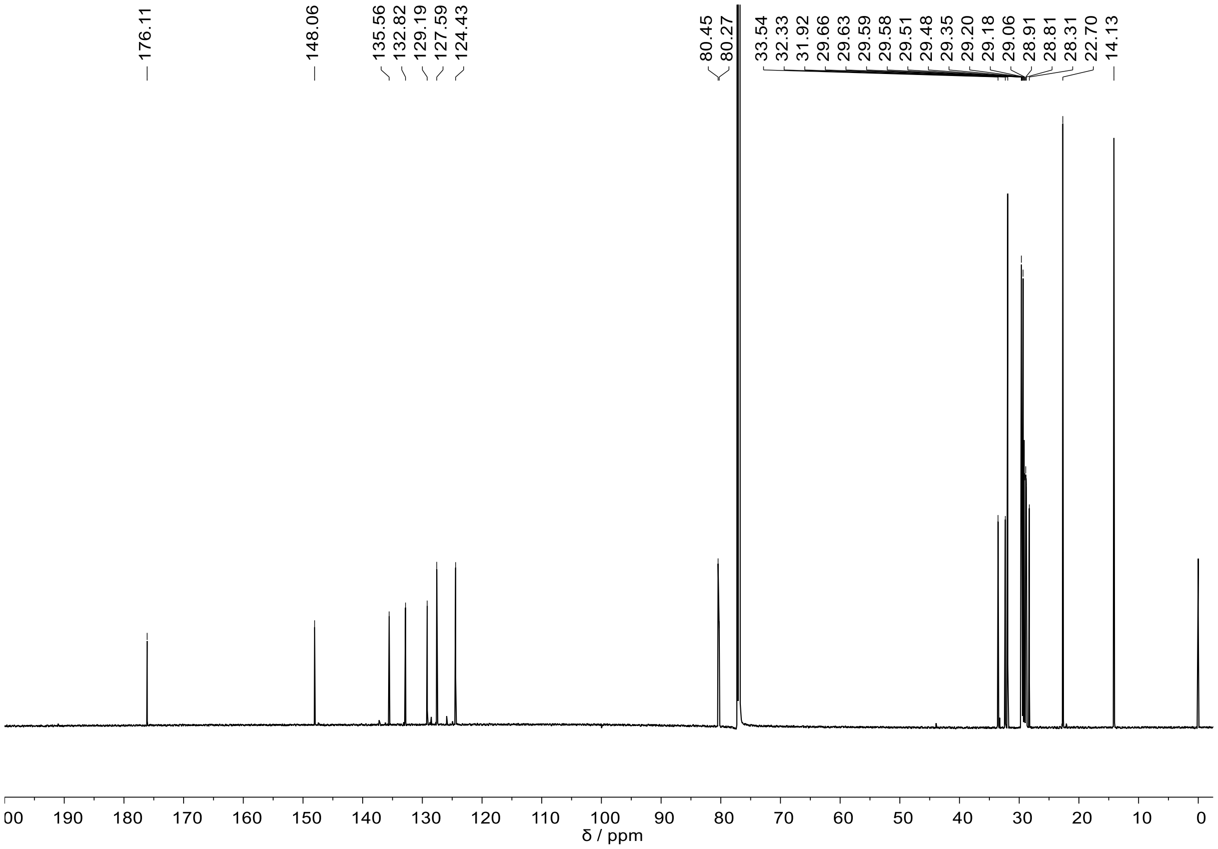

Figure S22: ^{1}H (top, at 500 MHz) and ^{13}C NMR (bottom, at 126 MHz) of **10b** in $CDCl_3$.

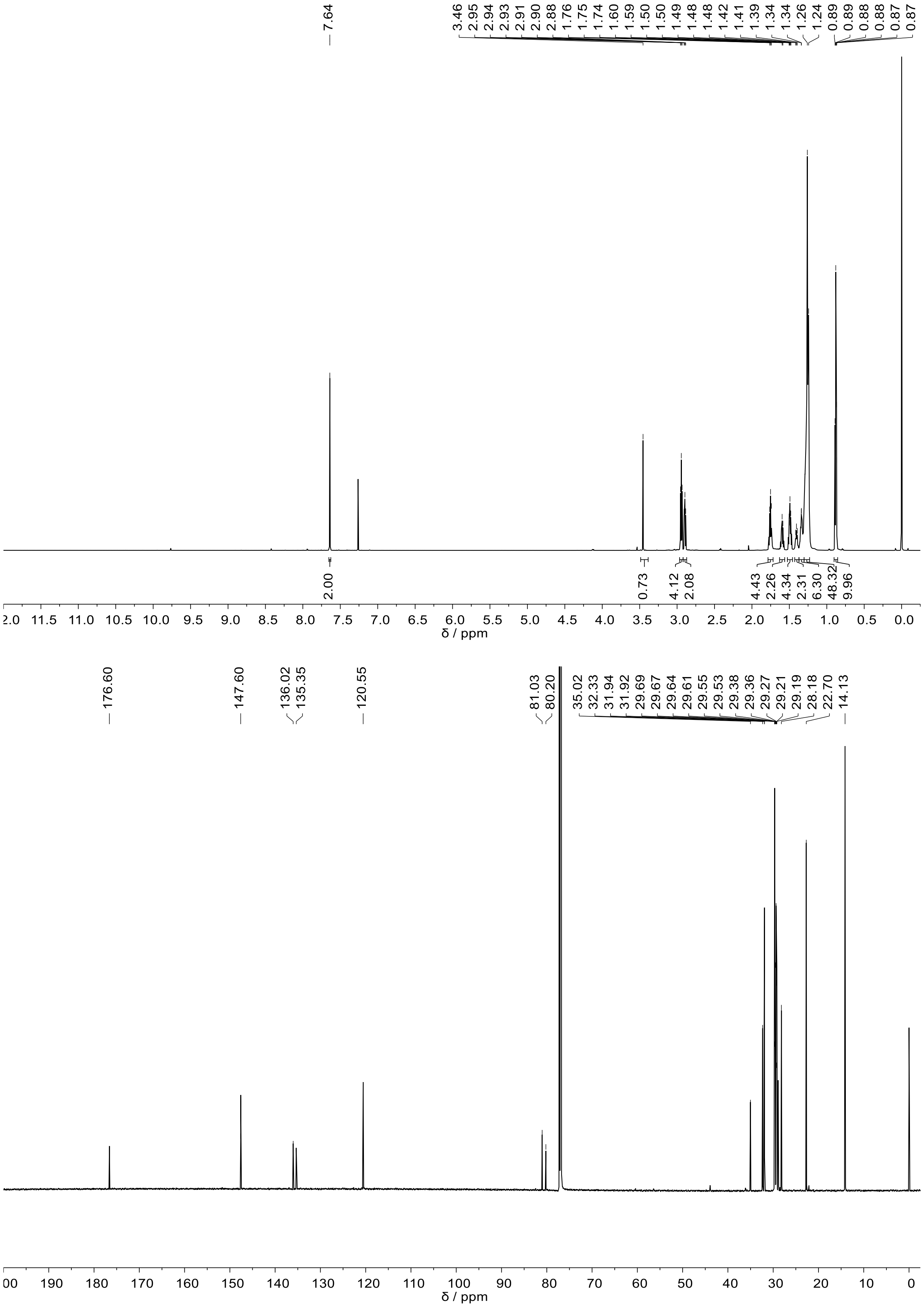

Figure S23: ^{1}H (top, at 700 MHz) and ^{13}C NMR (bottom, at 176 MHz) of **10c** in $CDCl_3$.

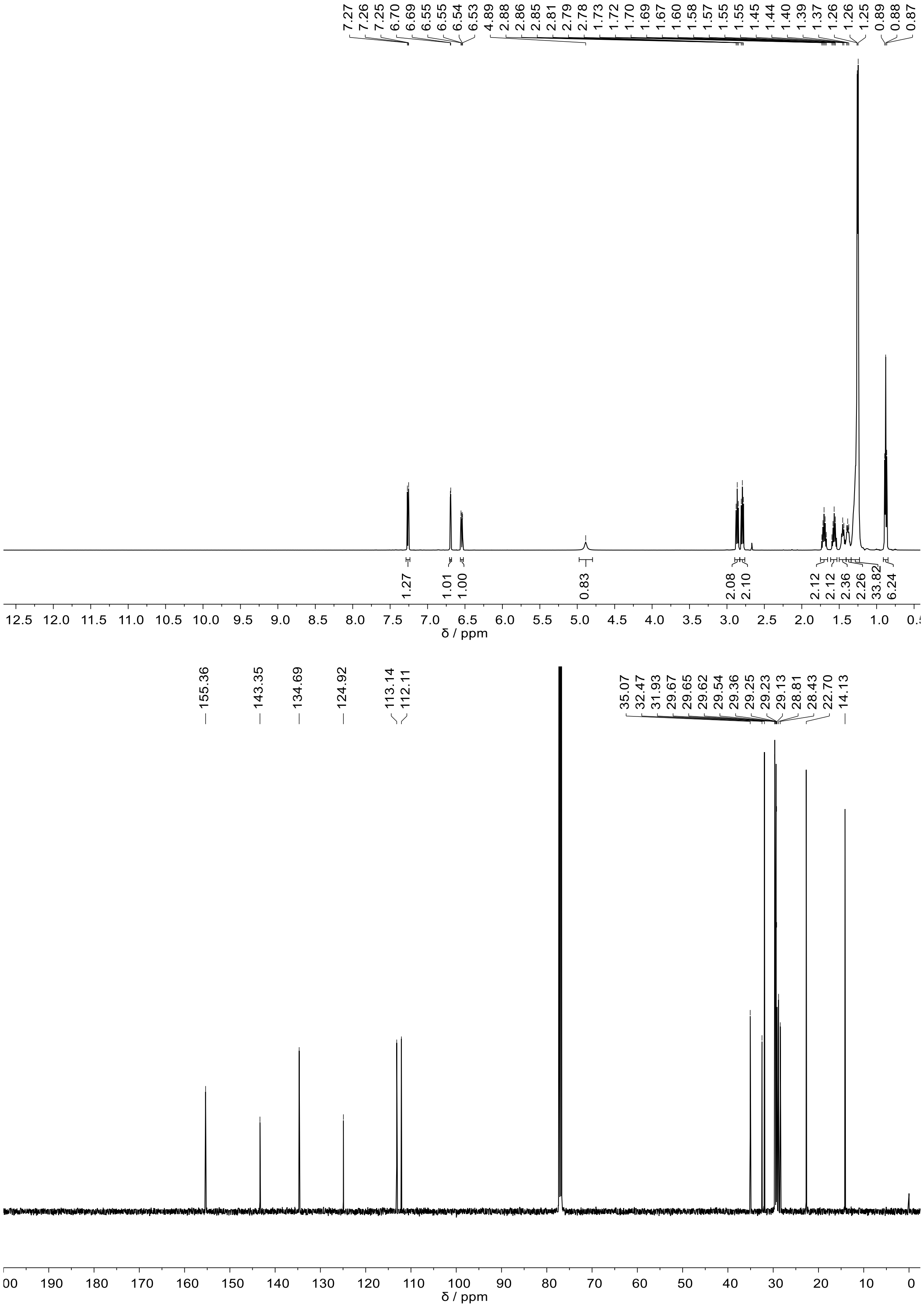

Figure S24: ^{1}H (top, at 500 MHz) and ^{13}C NMR (bottom, at 126 MHz) of **11b** in $CDCl_3$.

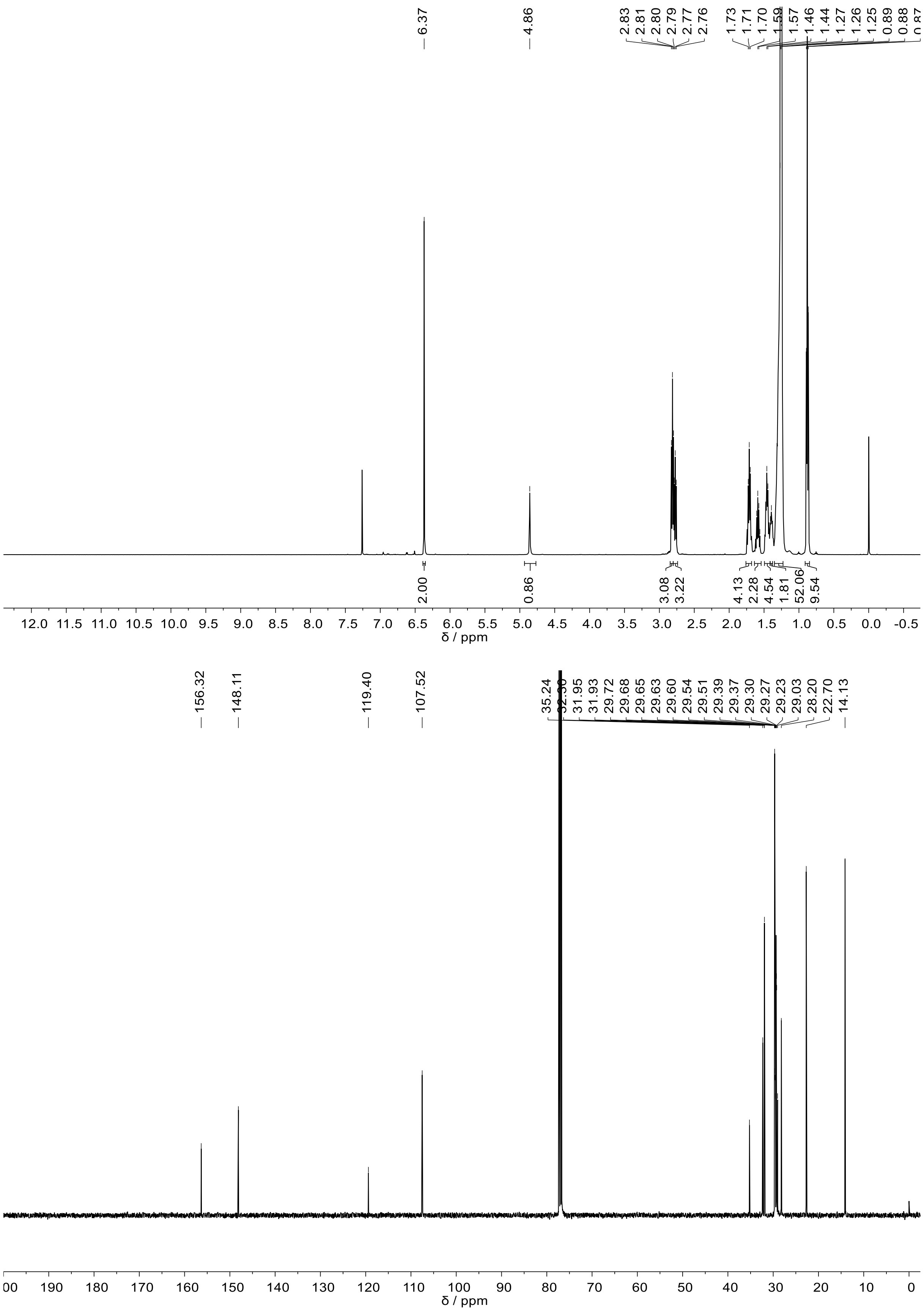

Figure S25: ^{1}H (top, at 500 MHz) and ^{13}C NMR (bottom, at 126 MHz) of **11c** in $CDCl_3$.

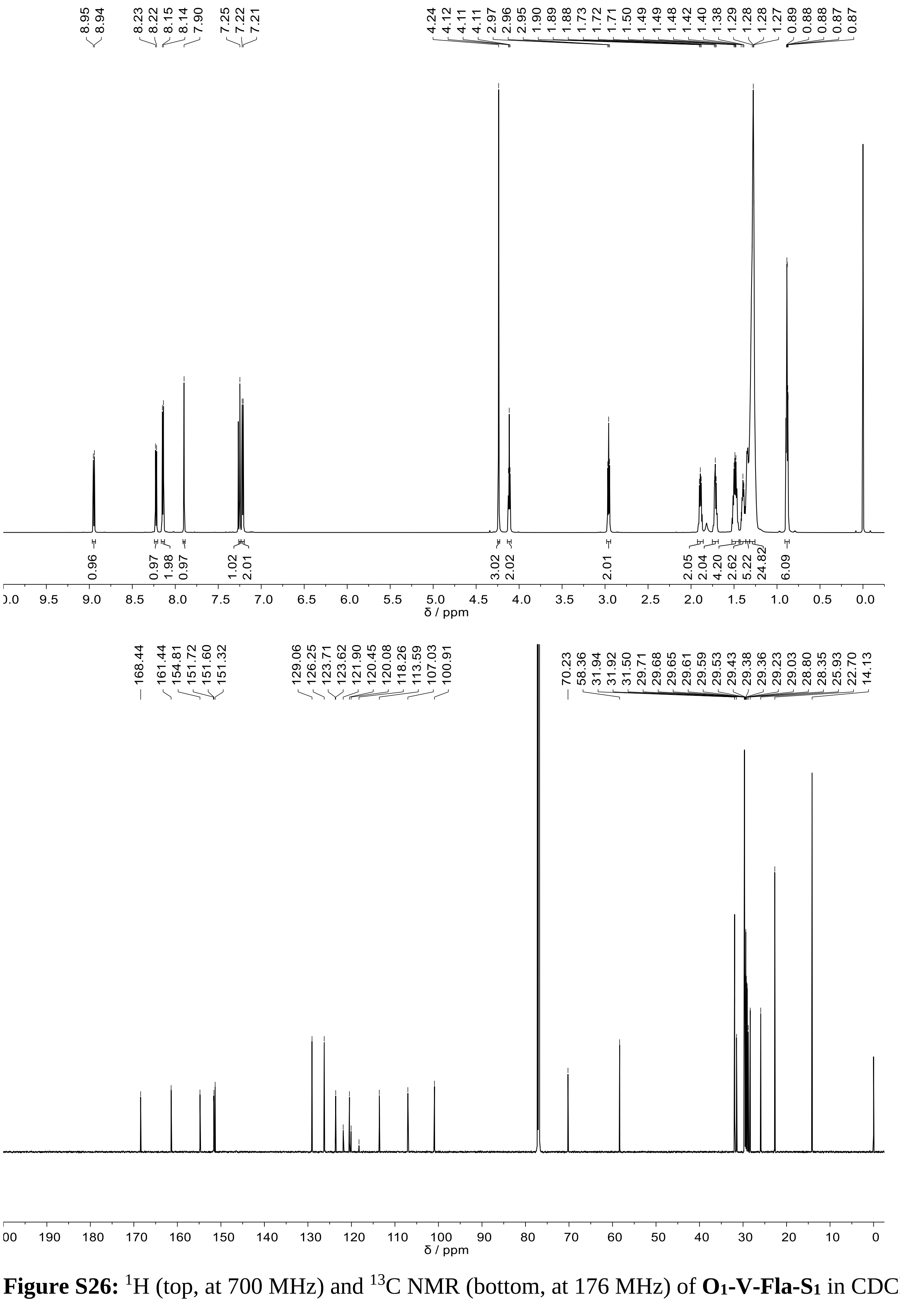

Figure S26: ^{1}H (top, at 700 MHz) and ^{13}C NMR (bottom, at 176 MHz) of **O_1-V-Fla-S_1** in $CDCl_3$.

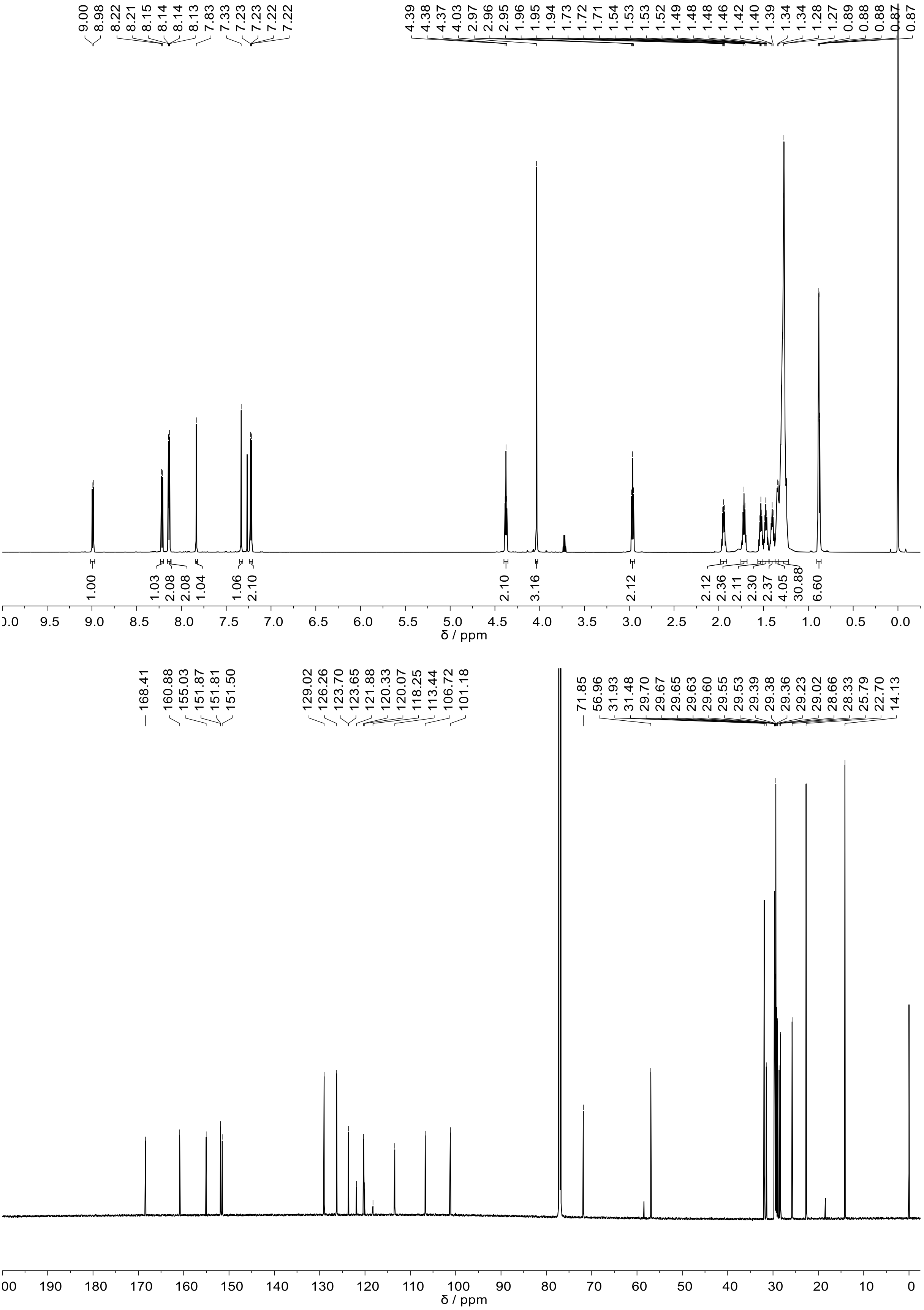

Figure S27: ^{1}H (top, at 700 MHz) and ^{13}C NMR (bottom, at 176 MHz) of **O_1-iV-Fla-S_1** in $CDCl_3$.

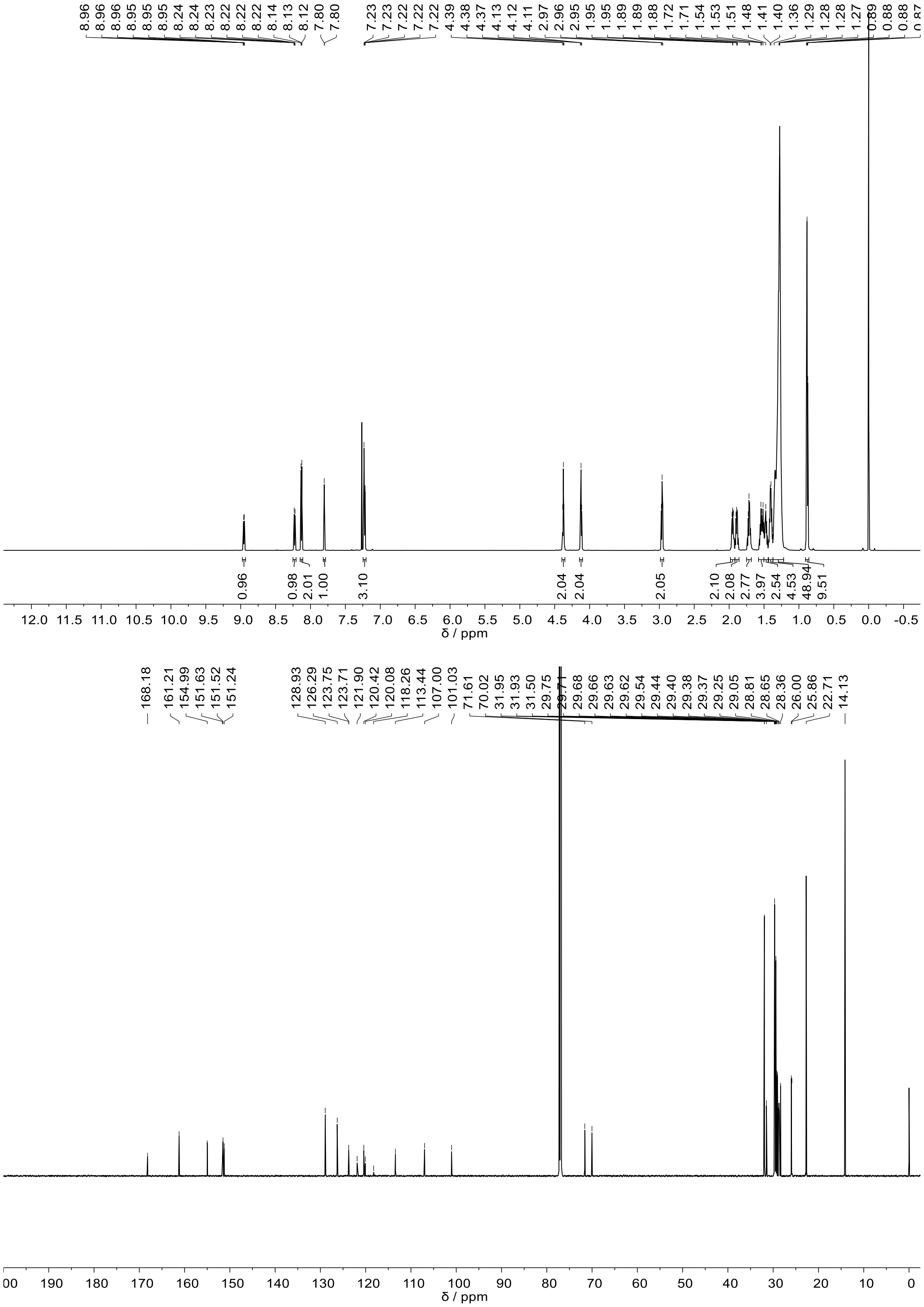

Figure S28: ^{1}H (top, at 700 MHz) and ^{13}C NMR (bottom, at 176 MHz) of **O_2-Fla-S_1** in $CDCl_3$.

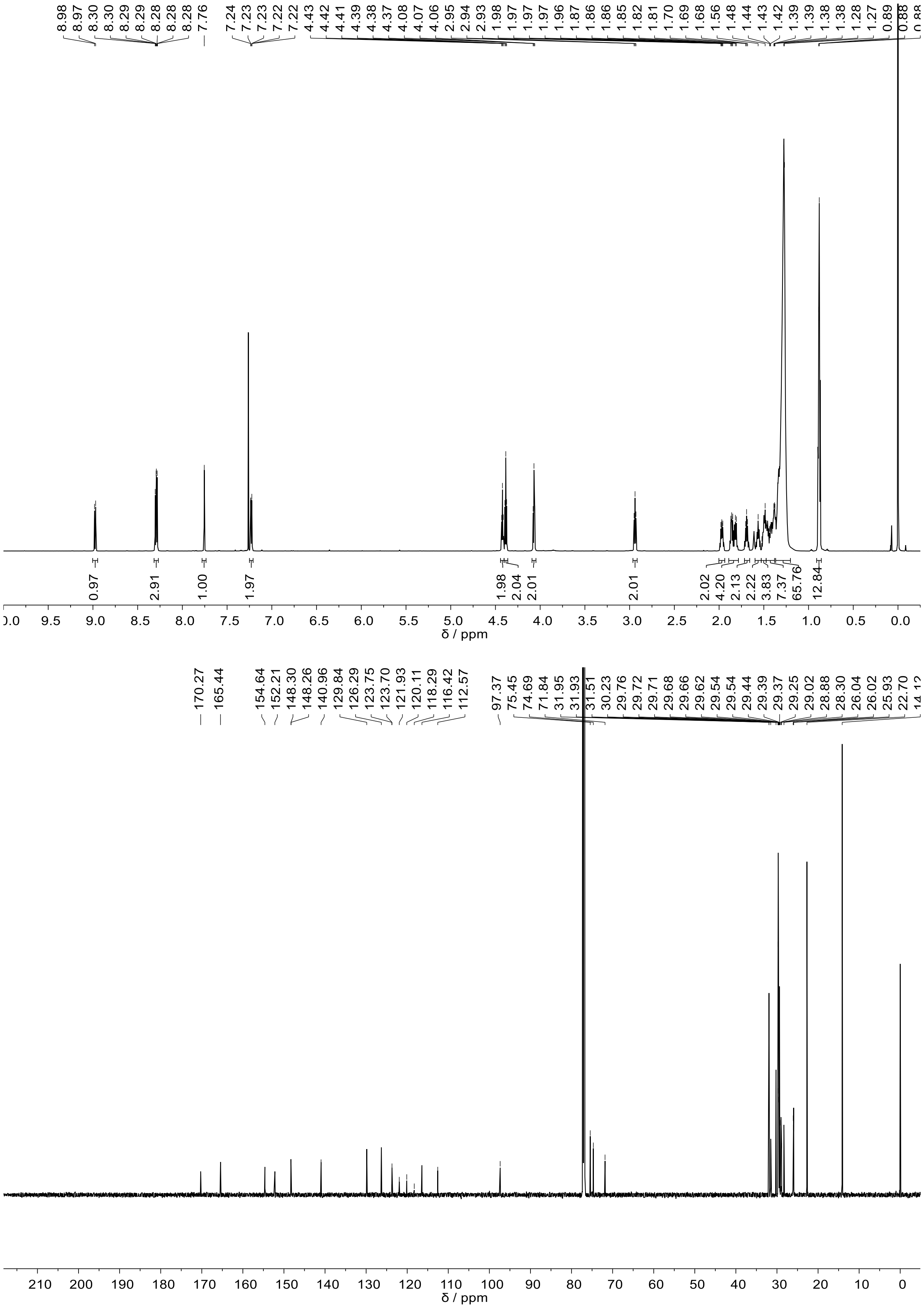

Figure S29: ^{1}H (top, at 700 MHz) and ^{13}C NMR (bottom, at 176 MHz) of **O₃-Fla-S₁** in $CDCl_3$.

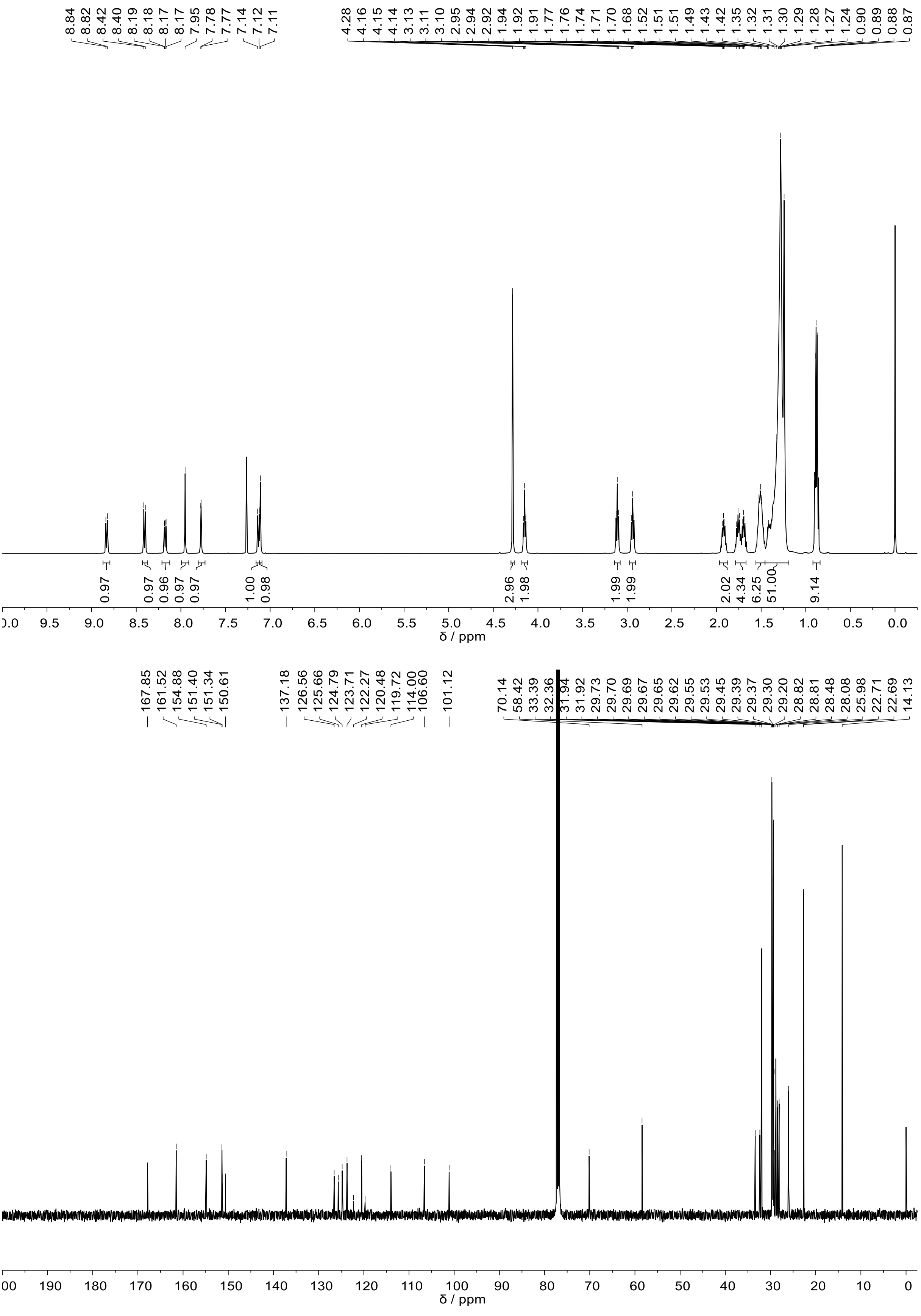

Figure S30: ^{1}H (top, at 500 MHz) and ^{13}C NMR (bottom, at 126 MHz) of **O_1-V-Fla-S_2** in $CDCl_3$.

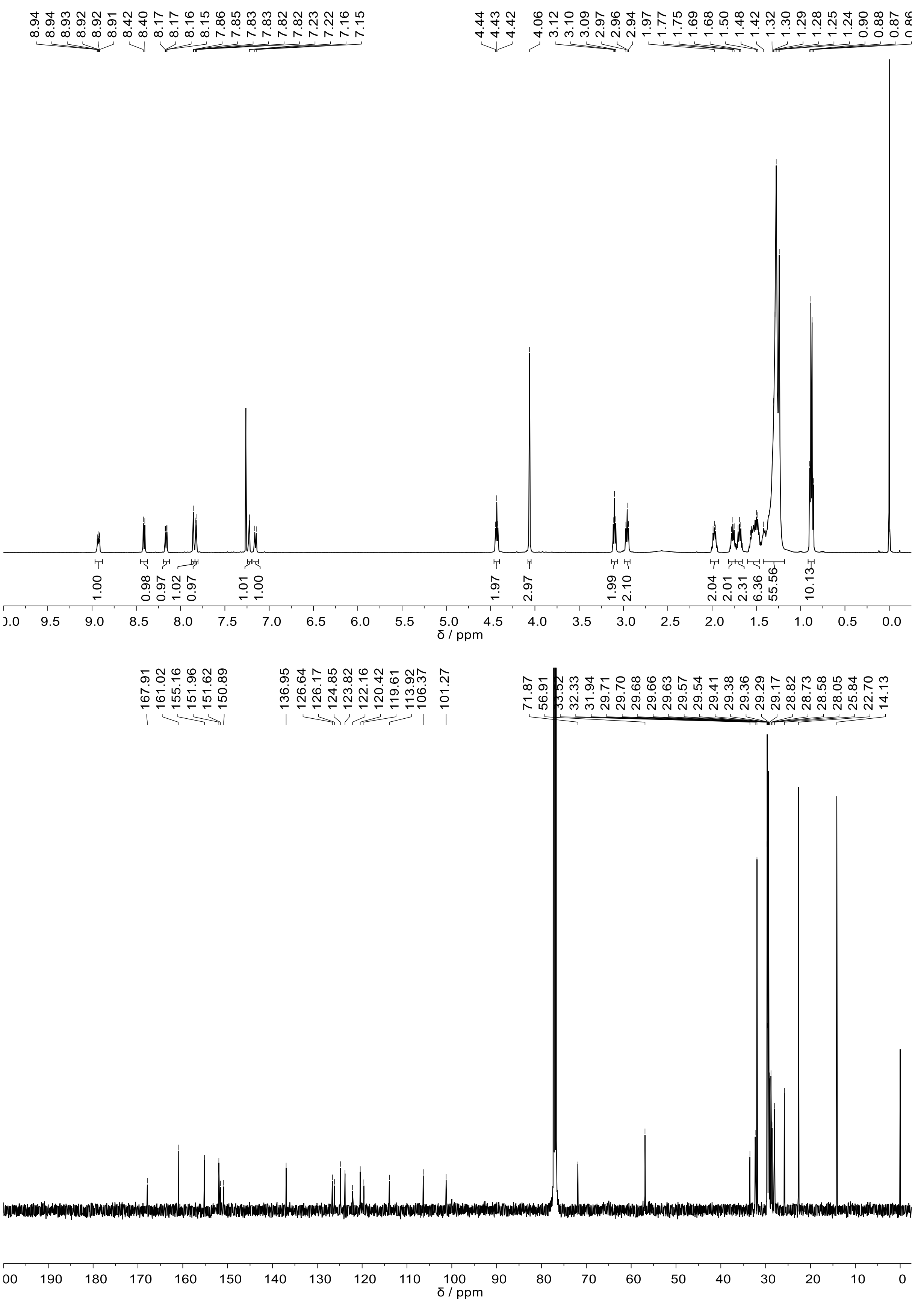

Figure S31: ^{1}H (top, at 500 MHz) and ^{13}C NMR (bottom, at 126 MHz) of **O₁-iV-Fla-S₂** in $CDCl_3$.

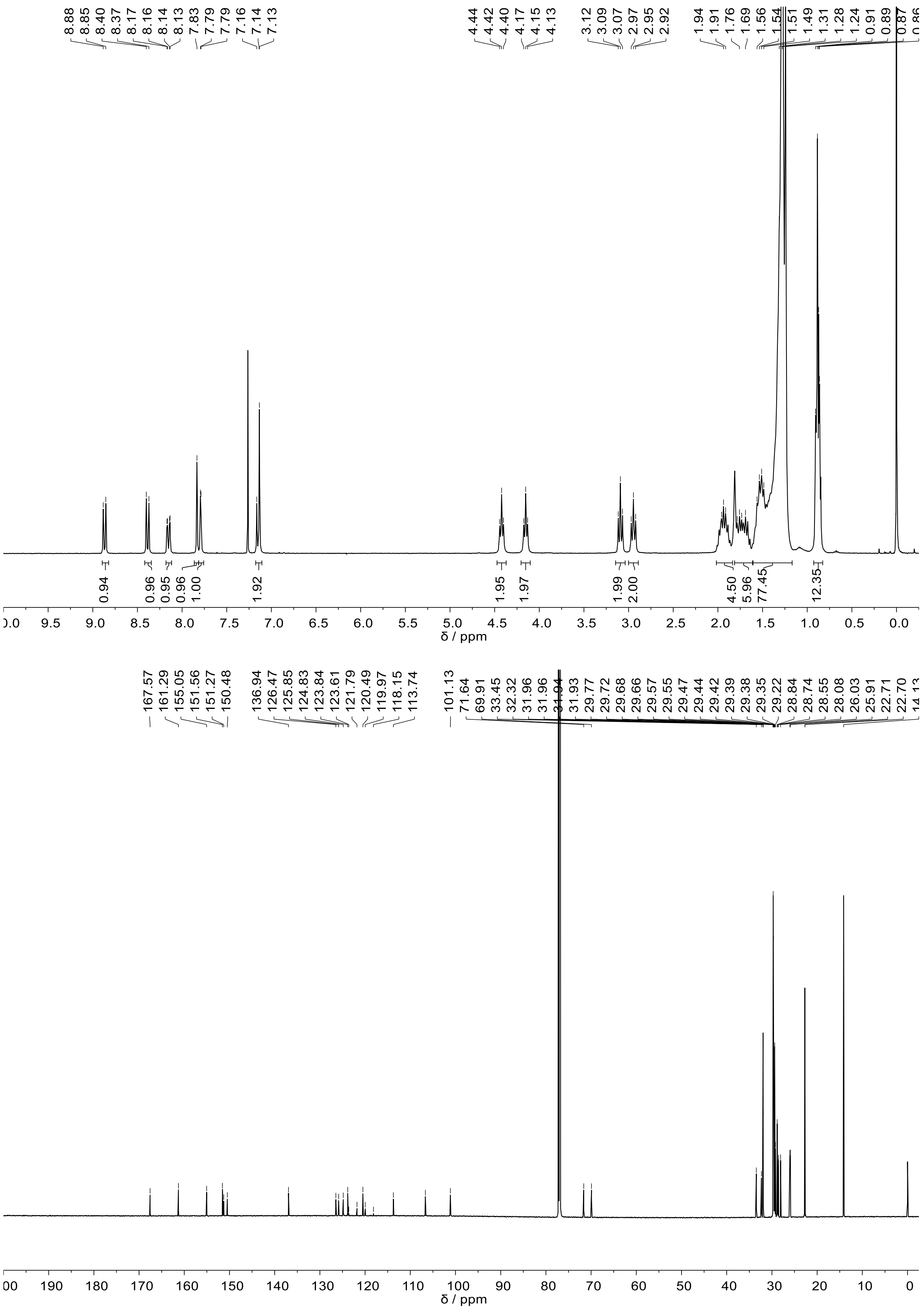

Figure S32: ^{1}H (top, at 300 MHz) and ^{13}C NMR (bottom, at 176 MHz) of **O_2-Fla-S_2** in $CDCl_3$.

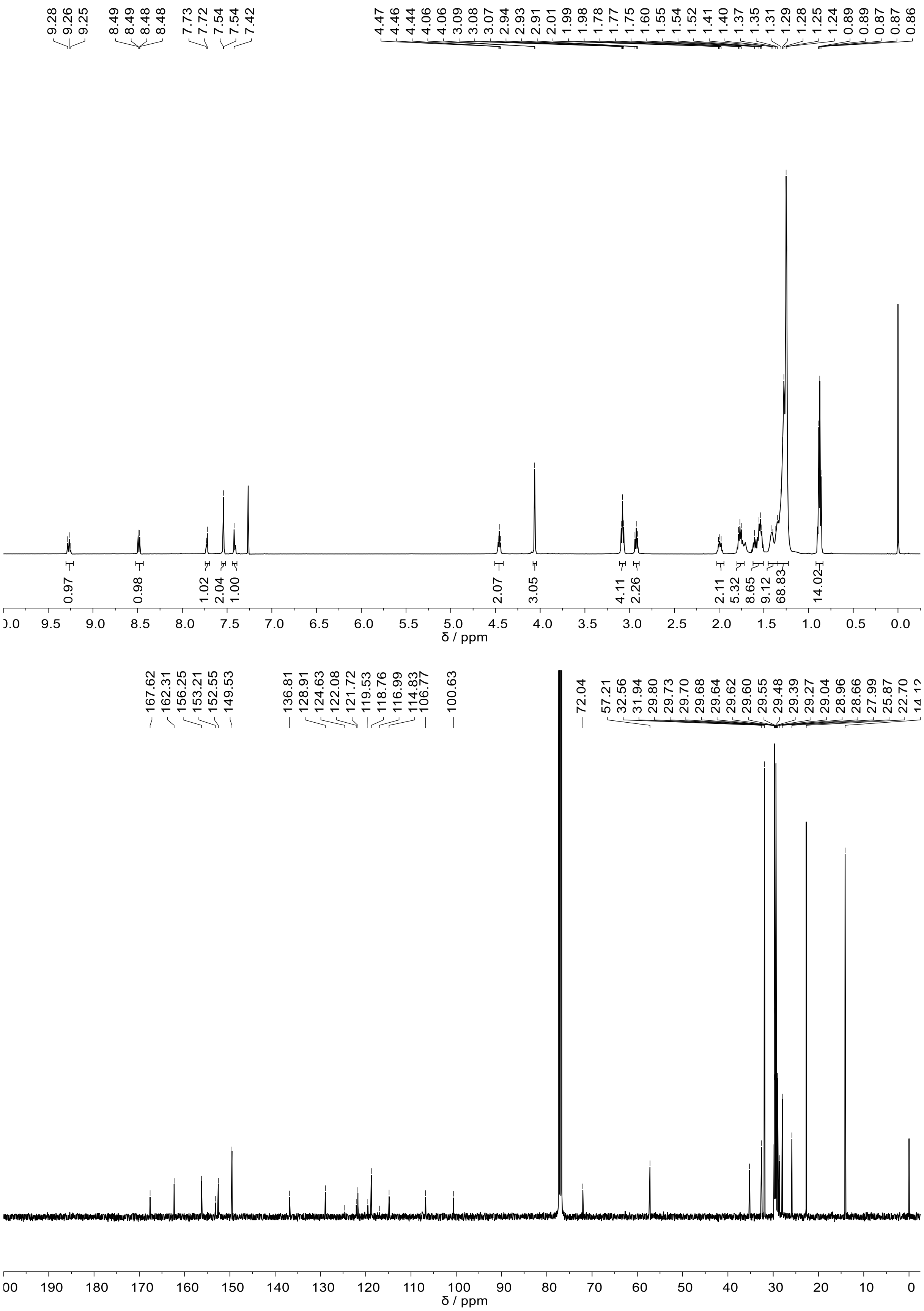

Figure S33: ^{1}H (top, at 500 MHz) and ^{13}C NMR (bottom, at 126 MHz) of **O_1-iV-Fla-S_3** in $CDCl_3$.

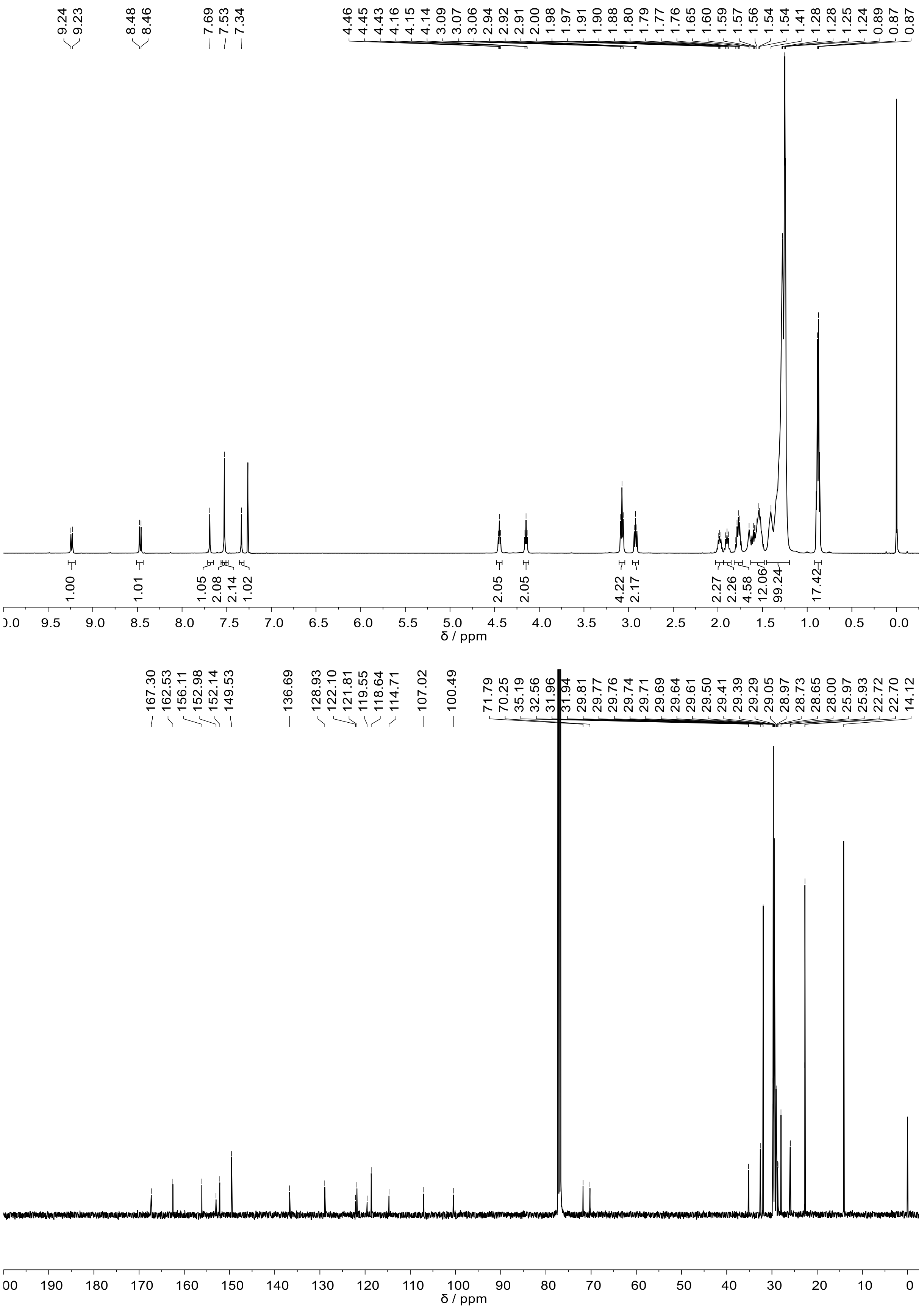

Figure S34: ^{1}H (top, at 500 MHz) and ^{13}C NMR (bottom, at 126 MHz) of **O_2-Fla-S_3** in $CDCl_3$.

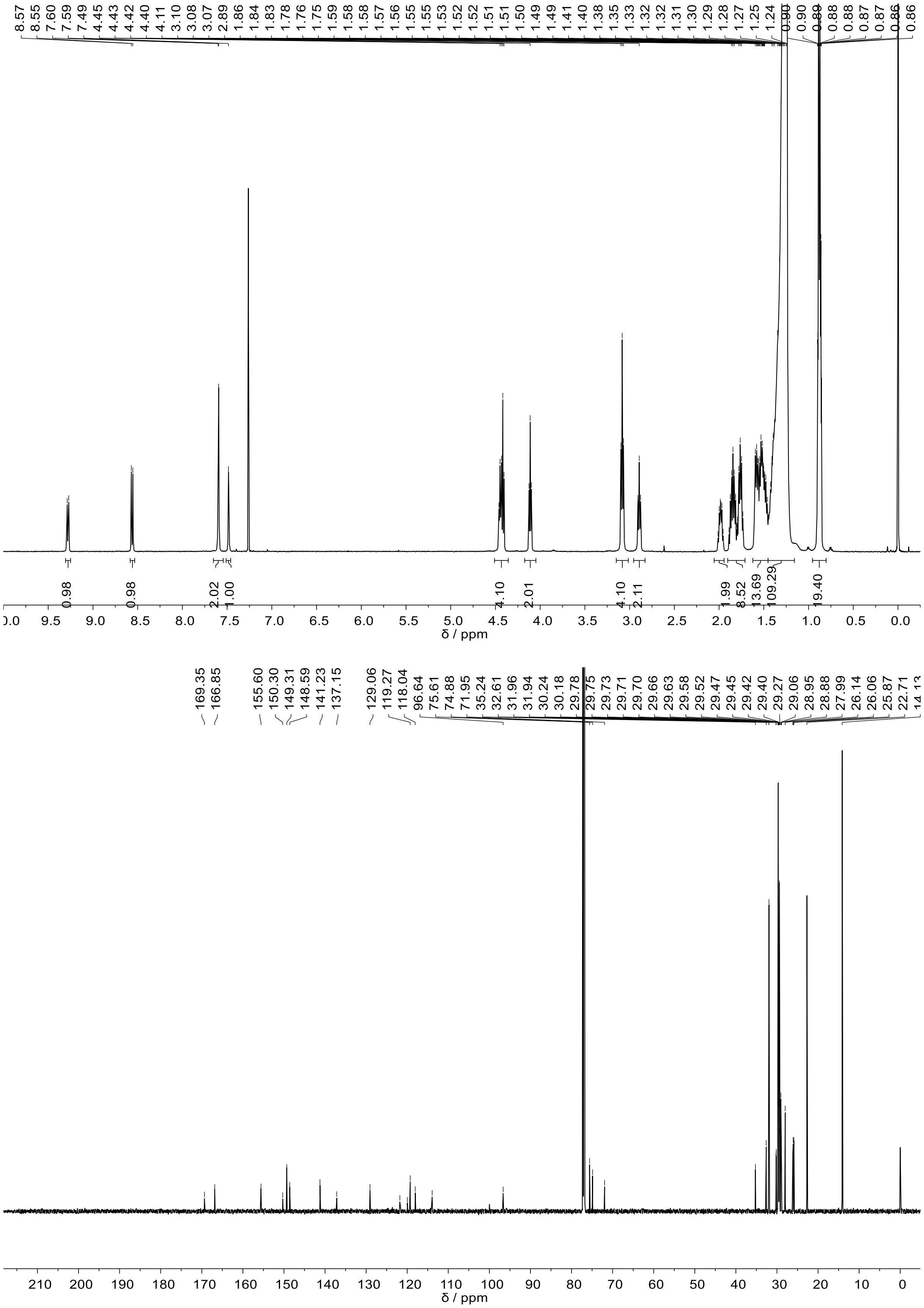

Figure S35: ^{1}H (top, at 500 MHz) and ^{13}C NMR (bottom, at 126 MHz) of **O$_3$-Fla-S$_3$** in $CDCl_3$.

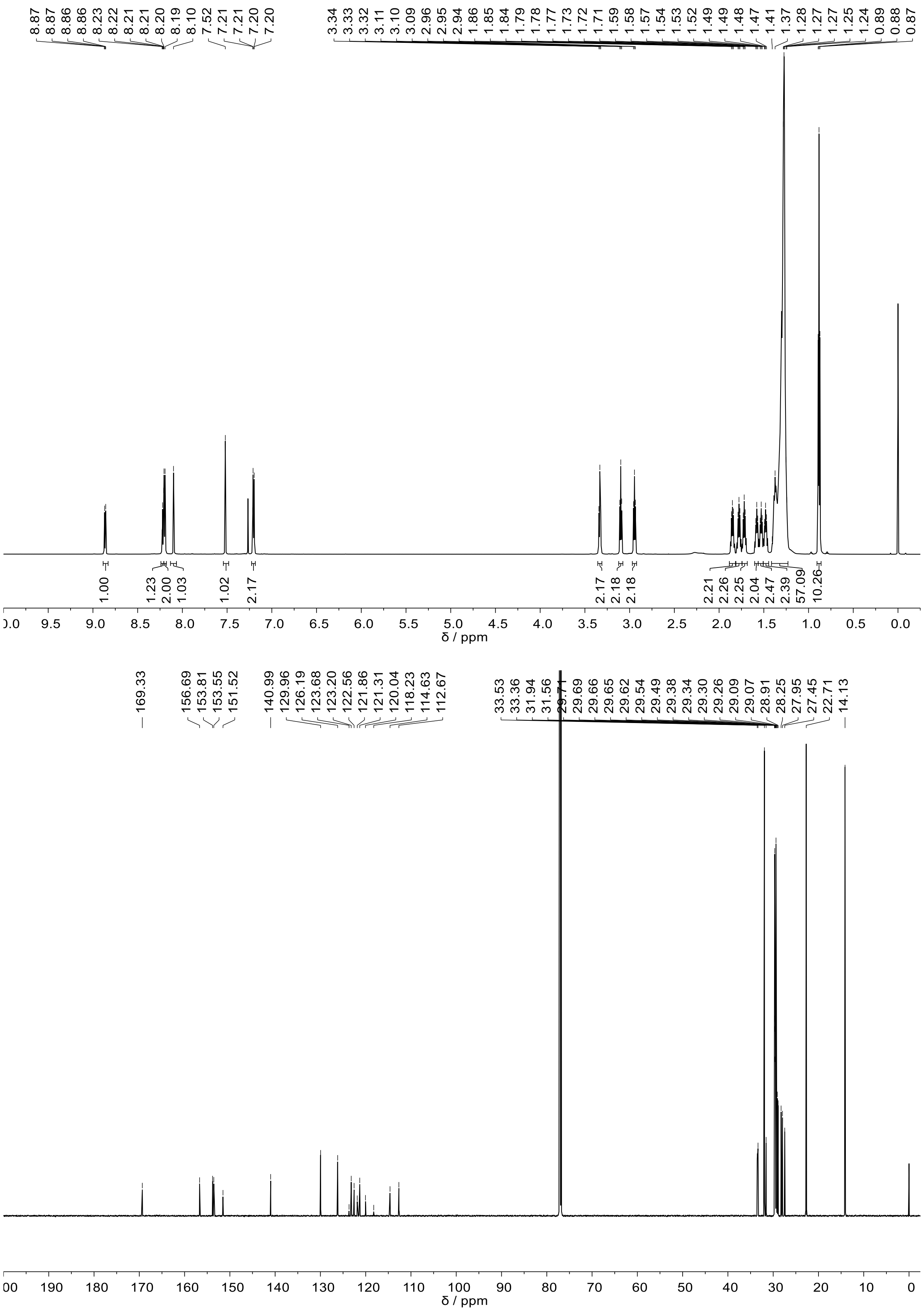

Figure S36: ^{1}H (top, at 700 MHz) and ^{13}C NMR (bottom, at 176 MHz) of **S_2-Fla-S_1** in $CDCl_3$.

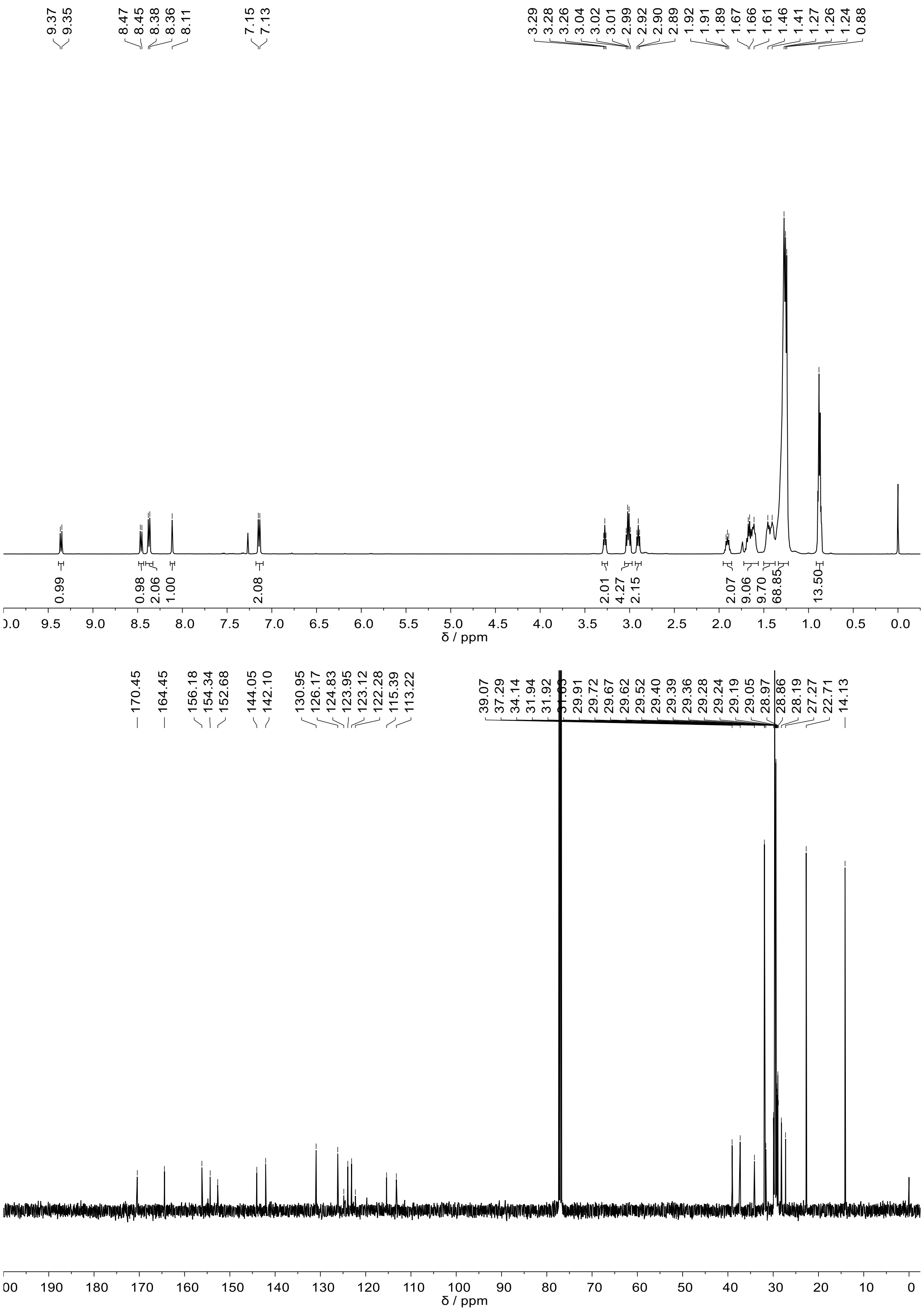

Figure S37: ^{1}H (top, at 500 MHz) and ^{13}C NMR (bottom, at 126 MHz) of **S$_3$-Fla-S$_1$** in $CDCl_3$.

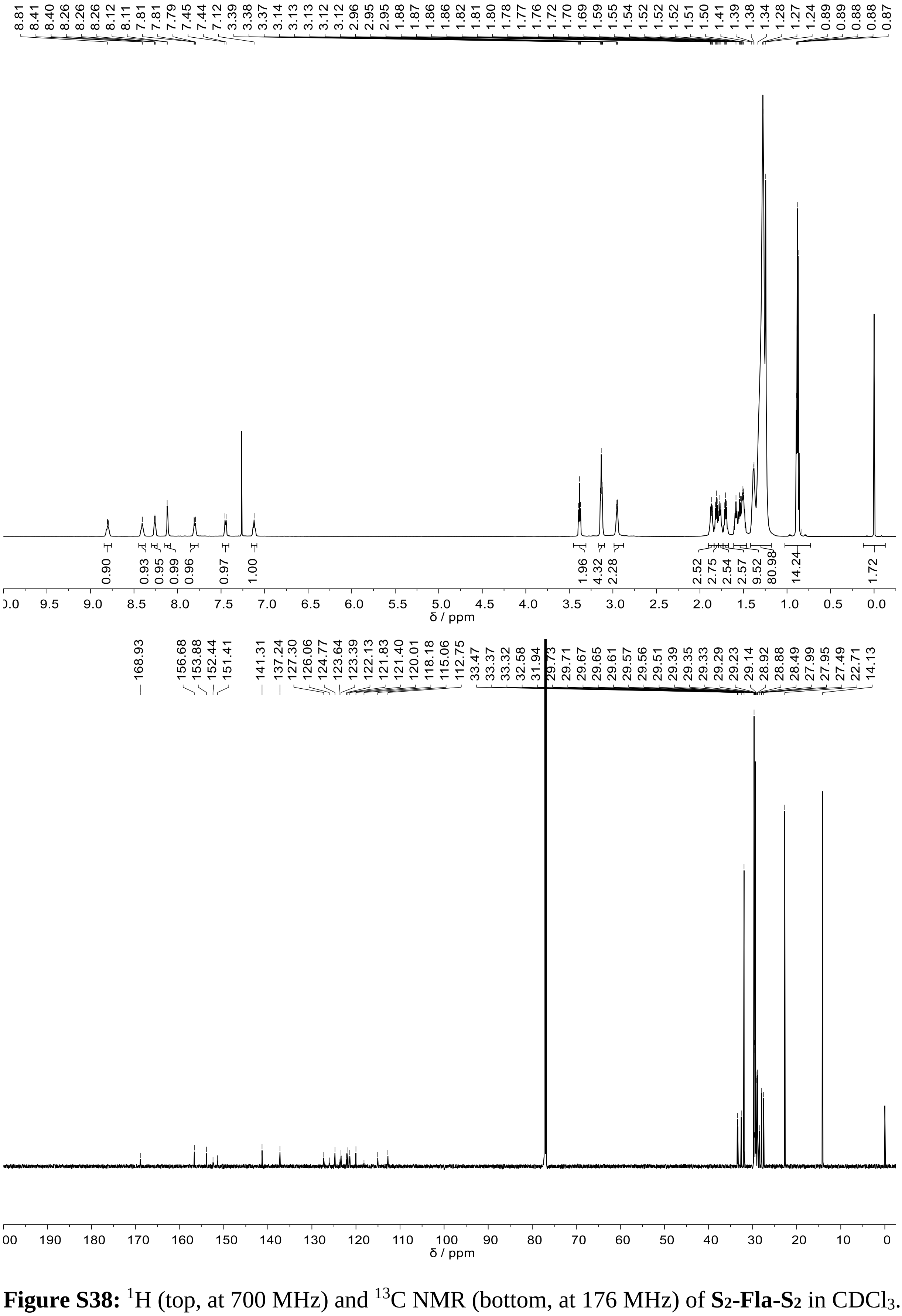

Figure S38: ^{1}H (top, at 700 MHz) and ^{13}C NMR (bottom, at 176 MHz) of **S$_2$-Fla-S$_2$** in $CDCl_3$.

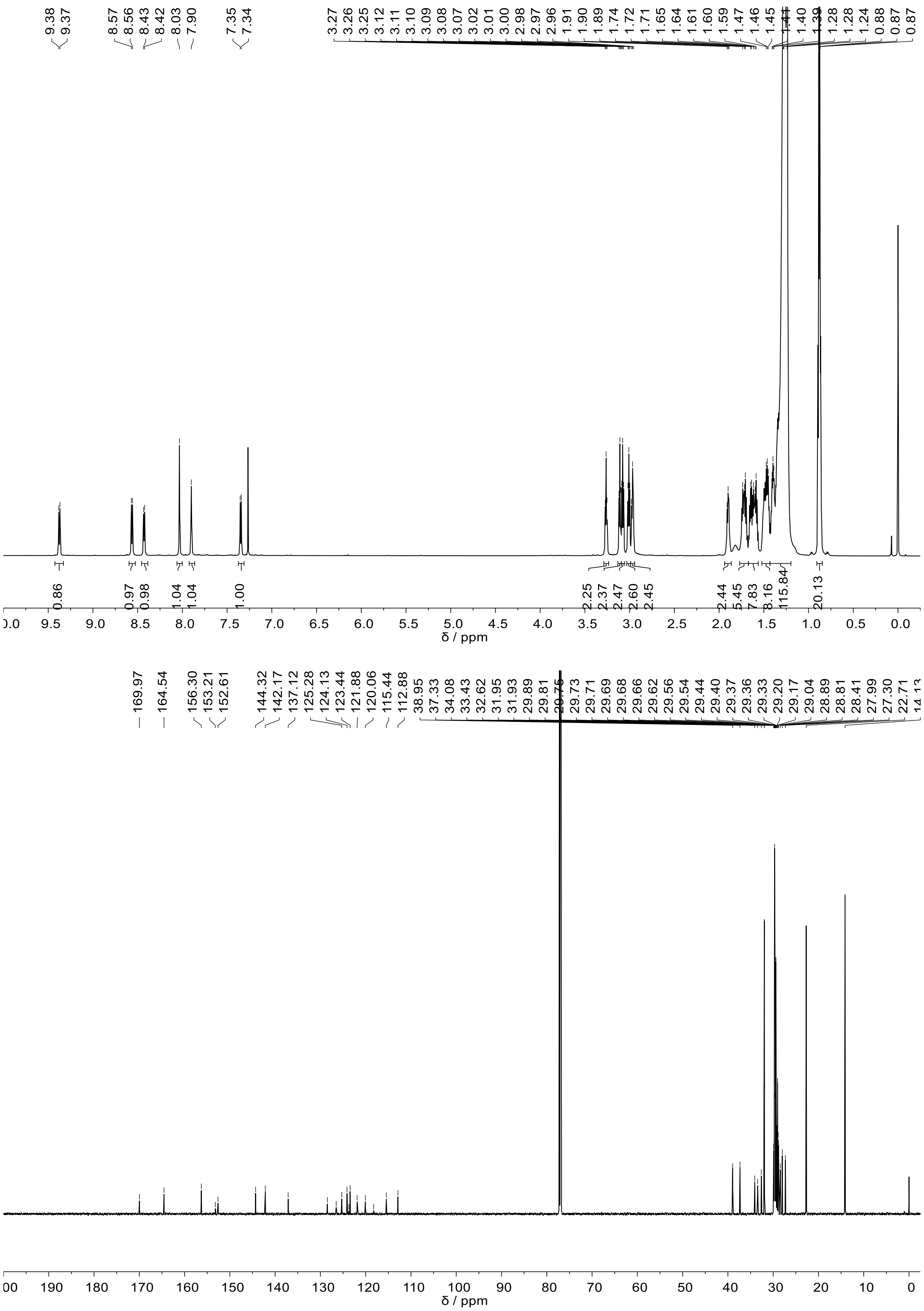

Figure S39: ^{1}H (top, at 500 MHz) and ^{13}C NMR (bottom, at 126 MHz) of **S_3-Fla-S_2** in $CDCl_3$.
